# Brazilian Nutritional Consensus in Hematopoietic Stem Cell Transplantation: children and adolescents

**DOI:** 10.31744/einstein_journal/2021AE5254

**Published:** 2021-11-25

**Authors:** Juliana Moura Nabarrete, Andrea Z Pereira, Adriana Garófolo, Adriana Seber, Angela Mandelli Venancio, Carlos Eduardo Setanni Grecco, Carmem Maria Sales Bonfim, Claudia Harumi Nakamura, Daieni Fernandes, Denise Johnsson Campos, Fernanda Luisa Ceragioli Oliveira, Flávia Krüger Cousseiro, Flávia Feijó Panico Rossi, Jocemara Gurmini, Karina Helena Canton Viani, Luciana Fernandes Guterres, Luiz Fernando Alves Lima Mantovani, Luiz Guilherme Darrigo, Maria Isabel Brandão Pires e Albuquerque, Melina Brumatti, Mirella Aparecida Neves, Natália Duran, Neysimelia Costa Villela, Victor Gottardello Zecchin, Juliana Folloni Fernandes

**Affiliations:** 1 Hospital Israelita Albert Einstein São Paulo SP Brazil Hospital Israelita Albert Einstein, São Paulo, SP, Brazil.; 2 Universidade Federal de São Paulo Instituto de Oncologia Pediátrica São Paulo SP Brazil Instituto de Oncologia Pediátrica, Universidade Federal de São Paulo, São Paulo, SP, Brazil.; 3 Universidade Federal de São Paulo São Paulo SP Brazil Universidade Federal de São Paulo, São Paulo, SP, Brazil.; 4 Universidade de São Paulo Faculdade de Medicina de Ribeirão Preto Hospital das Clínicas Ribeirão Preto SP Brazil Hospital das Clínicas, Faculdade de Medicina de Ribeirão Preto, Universidade de São Paulo, Ribeirão Preto, SP, Brazil.; 5 Universidade Federal do Paraná Hospital de Clínicas Curitiba SP Brazil Hospital de Clínicas, Universidade Federal do Paraná, Curitiba, PR, Brazil.; 6 Santa Casa de Misericórdia de Porto Alegre Porto Alegre RS Brazil Santa Casa de Misericórdia de Porto Alegre, Porto Alegre, RS, Brazil.; 7 Universidade Federal de São Paulo Escola Paulista de Medicina São Paulo SP Brazil Escola Paulista de Medicina, Universidade Federal de São Paulo, São Paulo, SP, Brazil.; 8 Universidade de São Paulo Faculdade de Medicina Hospital das Clínicas São Paulo SP Brazil Instituto da Criança, Hospital das Clínicas, Faculdade de Medicina, Universidade de São Paulo, São Paulo, SP, Brazil.; 9 Instituto Nacional de Câncer José Alencar Gomes da Silva Rio de Janeiro RJ Brazil Instituto Nacional de Câncer José Alencar Gomes da Silva - INCA, Rio de Janeiro, RJ, Brazil.; 10 Hospital de Câncer de Barretos Barretos SP Brazil Hospital de Câncer de Barretos, Barretos, SP, Brazil.

**Keywords:** Nutrition assessment, Nutritional status, Pediatrics, Child, Adolescent, Hematopoietic stem cell transplantation, Nutrition therapy

## Abstract

The Brazilian Nutritional Consensus in Hematopoietic Stem Cell Transplantation: Children and Adolescents was developed by dietitians, physicians, and pediatric hematologists from 10 Brazilian reference centers in hematopoietic stem cell transplantation. The aim was to emphasize the importance of nutritional status and body composition during treatment, as well as the main characteristics related to patient´s nutritional assessment. This consensus is intended to improve and standardize nutrition therapy during hematopoietic stem cell transplantation. The consensus was approved by the Brazilian Society of Bone Marrow Transplantation.

## INTRODUCTION

### Types of childhood hematopoietic stem cell transplantation and their applications

Hematopoietic stem cell transplantation (HSCT) is the infusion of cells involved for blood production to replicate inside the recipient and produce normal blood cells.^(1)^ The term “blood marrow transplantation” (BMT) has been used for many years, since blood marrow was the first source of blood stem cells used for transplantation.^(1)^

Hematopoietic stem cell transplantation are performed successfully since the 1960’s: three infants with congenital immunodeficiency received the marrow of their siblings and recovered from their disease, growing up to be healthy adults.^(1)^ It was a long journey until the milestone of 1 million transplantations worldwide was achieved in 2014.^(2)^ Currently, there are more than 1,500 centers performing transplantations in at least 75 countries. A large part of these centers report their results to the Center for International Blood and Marrow Transplant Research (CIBMTR) or the European Bone Marrow Transplantation (EBMT) and, on their websites, one can find summary statistical data, protocols, treatment guidelines and dozens of educational sessions and meetings.^(3–5)^

In Brazil, the Bone Marrow Transplantation (BMT) service of the *Hospital de Clínicas under the Universidade Federal do Paraná* was the first to initiate its activities in 1979. In 2016, 2,186 HSCTs were reported to the Brazilian Transplantation Registry (RBT) under the Brazilian Association for Organ Transplantation (ABTO). The overall transplantation rate per million inhabitants in our country reported to ABTO is 10.7, while countries such as England, Spain, the United States and Germany perform more than 50 transplantations per million inhabitants.^(2,6,7)^

In solid organ transplants, ABO compatibility (A, B, AB, O) is very important, but, in HSCTs, recipients’ red blood cells will switch to the donor blood group. In this case, the most important factor is human leukocyte antigen (HLA) compatibility.^(7)^

Human leukocyte antigen is fully encoded in chromosome 6, and altogether inherited ‘en bloc’ from each parent including alleles A, B, C (class I), DR, DP and DQ (class II). Depending on the number of alleles typed, matching could be of six antigens (A, B and DR), eight (A, B, C and DR), ten (A, B, C, DR, and DQ) or twelve.^(7)^ Therefore, siblings have a chance of having inherited two identical or distinct HLA chromosomes in 25% of the cases, and half of siblings have a chance of having inherited one equal and one different chromosome, known as haploidentical. In other words, we are always haploidentical to our parents and children.^(7)^

There are several types of HSCT, which are classified based on cell donor, cell source, degree of compatibility between donor and recipient, and type of conditioning, according to table 1.

**Table 1:** Types of childhood hematopoietic stem cell transplantations

Item	Type of transplantation
Hematopoietic cell donor	Autologous or autogenic: the patient themselves
	Syngeneic: the donor is an identical twin
	Allogeneic: the donor is a genetically distinct individual, usually with compatible HLA typing
Kinship between donor and recipient in allogeneic transplants	Related: family donors with blood ties
	Unrelated: adult donors (Registry of Volunteer Bone Marrow Donors) or umbilical cord blood units (umbilical cord/placental blood banks)
Conditioning regimen for transplantation	Myeloablative: using high doses of chemotherapy, either or not associated with radiotherapy. Without the hematopoietic stem cell infusion, this treatment would lead to death due to irreversible damage to the bone marrow
	Reduced toxicity transplantation: myeloablative transplantation, with reduced dosage of one or more drugs, for better tolerability
	Reduced intensity transplantation (not myeloablative, initially called mini-transplant): uses regimens that do not completely destroy the patient’s bone marrow. Based on immunosuppression, to prevent the recipient from rejecting donor cells. There is gradual switching from recipient cells to donor cells. Initially, patient and donor blood marrows produce blood cells together (mixed chimerism). Over a few days, the donor’s marrow goes on to occupy the entire marrow space and become the only responsible for blood production (complete chimerism, *i.e.*, 100% of bone marrow cells come from the donor)
Origin of hematopoietic stem cells in autologous or allogeneic transplants	Bone marrow: most common source in pediatric allogeneic transplants. The marrow is harvested in the operating room directly from iliac crests under general anesthesia
	Peripheral blood: cells are harvested by leukapheresis, after stimulation with growth factors (G-CSF). This process is called “mobilization”. In autologous transplants, before the onset of G-CSF, chemotherapy can also be used, such as high-dose cyclophosphamide. Peripheral blood stem cells are the most common source in autologous transplants
	Umbilical cord/placenta blood: collected and cryopreserved immediately after birth, usually used only in allogeneic transplants, particularly unrelated

HLA: Human Leukocyte Antigen; G-CSF: Granulocyte Colony-Stimulating Factor.

Before the start of conditioning, patients need a semi-implantable central venous catheter (permanent, such as Hickman, Permcath, or temporary), used for serial collection of test samples and for administration of chemotherapy, hydration, blood products and other medications. The days preceding the stem cell infusion are counted as negative and, after infusion, as positive.

The day before transplantation (D-1) is usually a one-day “break” to wait for chemotherapy metabolism and excretion, so that cells are not affected when infused. However, it is important to wait at least two terminal half-lives of the chemotherapy regimen before the infusion, which means less than 2 hours for agents such as melphalan or up to 4 days for carboplatin.^(1)^

The chemotherapy agents most commonly used in conditioning are cyclophosphamide, busulfan, cytarabine, etoposide, melphalan, carmustine, thiotepa, among others. Each chemotherapy agent has a distinct toxicity pattern. The drugs with the highest gastrointestinal toxicity are busulfan, etoposide, melphalan and thiotepa, as well as total body irradiation (TBI).^(1)^

The day of infusion, or the day of transplantation, is defined as day zero (D0).^(1)^

## AUTOLOGOUS HEMATOPOIETIC STEM CELL TRANSPLANTATION

### Cell harvesting procedure

In autologous transplants, the patient’s own stem cells are usually harvested from peripheral blood, cryopreserved and re-infused after the patient receives high-dose chemotherapy (conditioning or preparative regimen). Thus, it is possible to use myeloablative doses of chemotherapy and then provide hematological recovery.

For mobilization, the patient is subjected to a few steps. It starts with chemotherapy (optional, usually 1g/m^2^/day to 2g/m^2^/day cyclophosphamide).

Daily use of granulocyte colony-stimulating factor (G-CSF) in one or two intravenous (IV) or subcutaneous applications is then initiated, until stem cells can be harvested by leukapheresis or are considered to have failed mobilization. Throughout this period, the number of CD34+ stem cells in peripheral blood is monitored by flow cytometry, and when the appropriate level is reached (usually higher than 10 cells/mcL to 20 cells/mcL), that is usually the ideal time for harvesting by means of a dual lumen central venous catheter suitable for hemodialysis – temporary (*e.g.*, Shilley) or permanent (for example: Permcath) –, and the start of leukapheresis.

In leukapheresis, the patient’s blood is continuously processed in a cell separation device. Leukocytes are slowly collected into a specific bag, and red blood cells and plasma are continuously returned through the lumen of the catheter. Leukapheresis takes a few hours and can be repeated the next day if not enough cells are harvested for transplantation – usually 3 to 5 million CD34 cells/kg of body weight per transplant are required. Some pediatric regimens for central nervous system tumors, neuroblastoma, and germ cell tumors require up to three sequential cycles of high-dose chemotherapy, and a large number of cells may be required.

The harvested cells are frozen (cryopreserved) at specialized laboratories using solutions containing dimethyl sulfoxide (DMSO), a cryoprotectant that minimizes damage caused by freezing and thawing. Stem cells can be stored in a freezer at -80°C for several months and in nitrogen tanks for decades until they are needed.

### During transplantation

For transplantations, children need a permanent semi-implantable catheter (Hickman, Permcath or others) or a temporary semi-implantable catheter to receive the cells and the prior chemotherapy.

On negative days (D-6, D-5, D-4, D-3, and D-2), the conditioning or preparative regimen with high-dose chemotherapy is administered to destroy tumor cells resistant to conventional chemotherapy regimens and doses. Then there is a “break” from chemotherapy, usually on D-1.

On D0, autologous cells are thawed and infused through the central venous catheter. The infusion may have several adverse effects due to the presence of the cryoprotectant, the most frequent of which are characteristic smell in the breath due to DMSO excretion (described as corn cream, garlic etc.), bradycardia, pressure changes, nausea, and vomiting.

As of D+1, the day after infusion, patients may be initiated (or not) on daily G-CSF, in addition to drugs for anti-infection prophylaxis (viruses, fungi, and bacteria), which may vary according to institutional protocols. In the days following infusion, patients may also present with substantial chemotherapy-induced gastrointestinal toxicity, with oral mucositis, nausea, vomiting and diarrhea, which may cause intense discomfort and pain, requiring potent analgesics.

Due to bone marrow suppression, patients have anemia and thrombocytopenia, requiring frequent transfusions, as well as neutropenia, with high susceptibility to infections. Patients commonly have fever which, even without a defined focus, requires management with broad-spectrum antibiotics.

The “bone marrow engraftment” is the first of 3 consecutive days after chemotherapy nadir, in which patients with 500 neutrophils/mm^3^ or more in their blood count. The definition of platelet engraftment, in general, is the first day with more than 20 thousand platelets/mm^3^ after 7 or more days without transfusion.

The complexity and complications are lower in autologous transplantations when compared with allogeneic. From the nutritional standpoint, however, some patients have been receiving chemotherapy for several months when they undergo BMT and, therefore, may have significant nutritional deficits. Also, some children need to receive radiation therapy after transplantation. When the radiation field involves the esophagus or abdomen, this may lead to mucosal injury, nausea, vomiting and diarrhea, further delaying nutritional recovery.

Autologous transplantations are the most frequent modality in adults, used in the treatment of multiple myeloma and lymphomas.^(6)^ In pediatrics, there is evidence that autologous BMT can provide greater survival in Hodgkin’s lymphoma, non-Hodgkin’s lymphomas in second or greater remissions, and chemosensitive solid tumors, such as germ cell tumors in second remission or high-risk neuroblastoma.^(6)^ In infants with central nervous system tumors, autologous HSCT may be used to replace or postpone radiation therapy, whose long-term harmful effects on the developing brain would be devastating.^(6)^ Some studies indicate the benefit of BMT in the treatment of Ewing sarcoma, metastatic or in second remission; central nervous system primitive tumors, disseminated or in second remission; extraocular retinoblastoma; and Wilms’ tumor after the second remission.

Autologous transplants can also be used to eradicate self-reactive cells in the treatment of severe autoimmune diseases, and in countries with appropriate infrastructure, autologous stem cells can be used for laboratory-based genetic repair, also known as “gene therapy”.

### Allogeneic transplantation

In allogenic transplants, classical myeloablative conditioning regimens in children are 120mg/kg to 200mg/kg cyclophosphamide with 1,200 cGy TBI for conditioning of acute lymphocytic leukemia, and 200mg/kg cyclophosphamide with busulfan for myeloid leukemias. For non-malignant diseases, busulfan and fludarabine or melphalan are often used.^(5)^

In addition to chemotherapy, the preparative regimen includes drugs to reduce the chance of recipients destroying donor cells (rejection), such as anti-thymocyte globulin, mainly in unrelated transplants, and cyclosporine, and the chance of donor cells attacking the recipient, causing graft *versus* host disease (GVHD).^(3,5)^

Donor stem cells are usually harvested and infused on D0. To treat malignancies, cells can be harvested from peripheral blood, using G-CSF alone, without chemotherapy, if leukapheresis is feasible by peripheral venous access, without the need for a central venous catheter.

Bone marrow is the most common source for allogeneic HSCT.^(3)^ For bone marrow harvesting, the donor is taken to the operating room and, under general anesthesia and/or spinal block (epidural), cells are harvested from posterior iliac crests with needles and syringes. Approximately 15mL of bone marrow/kg of recipient body weight to 20mL/kg of donor body weight is retrieved. Harvesting takes about 1 to 2 hours and, usually, the donor is discharged on the same day or the next day. In unrelated transplantations, it may be necessary to harvest cells up to 2 days before infusion, which generally grants excellent viability.

Other drugs used after transplantation to prevent GVHD are cyclosporine, tacrolimus (calcineurin inhibitors), methotrexate (D+1, D+3, D+6 and sometimes also on D+11) and mycophenolate mofetil. Recently, high-dose cyclophosphamide, infused on D+3 and D+4, has been used, mainly in haploidentical transplants.

Allogeneic transplantations are mainly used in the treatment of leukemias, myelodysplasia and bone marrow failures, among others.^(6)^ Donor cells are infused after administration of the conditioning regimen, which includes high dose-chemotherapy or TBI.

Although donor cells can recognize the recipient as foreign and attack it (GVHD), these same cells can recognize and destroy tumor cells, decreasing the chance of recurrent malignancy (graft *versus* tumor effect or *graft-versus-leukemia* – GVL).

The maximum toxicity associated with HSCT is usually expressed as transplant-related mortality (TRM), *i.e.*, mortality in the first 100 days after infusion of stem cells due to any causes, except recurrence of the underlying disease. Transplant-related mortality increases greatly if the disease is in advanced stages. Results published by the CIBMTR show 2% to 10% of TRM in autologous HSCT, 7% to 22% in related allogeneic, and 10% to 25% in unrelated allogeneic.^(4)^

The toxicities presented can be grouped as acute and frequent, and less frequent and more severe, according to table 2.

**Table 2:** Allogeneic hematopoietic stem cells transplantation-related toxicities

Acute and frequent	Lower frequency and higher severity
Oral mucositis all over the gastrointestinal tract	Hepatic sinusoidal obstruction syndrome (or veno-occlusive disease)
Nausea, vomiting and diarrhea of varying intensity	Hemorrhagic cystitis due to drug toxicity or viral infections
Fever, usually due to bacterial infections, especially during neutropenia, before bone marrow engraftment	Invasive fungal infections
	Viral infections
Mild to moderate renal failure induced by irradiation and drugs	Graft rejection (graft failure)
Transient arterial hypertension due to the use of cyclosporin and/or corticosteroids	Idiopathic or infectious interstitial pneumonitis, mainly by cytomegalovirus and respiratory viruses.
	Other viral infections
Acute GVHD	Cardiac toxicity
	Central nervous system toxicity – seizures and stroke
	Thrombotic microangiopathy

Source: Pasquini M, Wang Z, Horowitz MM, Gale RP. 2013 report from the Center for International Blood and Marrow Transplant Research (CIBMTR): current uses and outcomes of hematopoietic cell transplants for blood and bone marrow disorders. Clin Transpl. 2013:187-97.^(4)^

GVHD: graft *versus* host disease.

Infectious complications usually follow a somewhat constant pattern. In the first 30 days, patients with neutropenia and mucositis are predisposed to infections by the herpes simplex virus, respiratory viruses, *Gram*-positive and negative bacteria, and *Candida* sp. From then on, GVHD becomes the main predisposing factor for infections. Respiratory viruses, adenovirus, cytomegalovirus, and filamentous fungal infections remain the main infectious agents between months 1 and 2.

After engraftment, severe immunodeficiency persists for several months, particularly after allogeneic and autologous transplants with rituximab therapy for lymphomas. Infections by cytomegalovirus, adenovirus, herpes-zoster, *Pneumocystis carinii*, and toxoplasmosis are frequent and, in patients who develop chronic GVHD (cGVHD) or splenectomy patients, there is a risk of fulminant infections by encapsulated bacteria.

Splenectomy patients with prior splenic radiation therapy or who had cGVHD must be extensively educated on the higher risk for fulminant sepsis, as well as receive continuous prophylaxis against encapsulated bacteria and be instructed to seek a referral medical facility in case of fever, to have a blood culture collected and appropriate antibiotics immediately initiated, such as amoxicillin-clavulanate or ceftriaxone.

Due to the short- and long-term risks associated with transplants, their indication is restricted to diseases in which survival with HSCT is higher than survival with conventional treatment (*e.g.*, chemotherapy), or when transplantation can promote a significant improvement in quality of life, such as eliminating the need for hypertransfusion and long-term complications in patients with hemoglobinopathies.

Hematopoietic stem cell transplantation indications are the object of ongoing reevaluation. Ideally, transplant indications should be based on results from randomized clinical studies, but this is not always possible. The only way to make precise indications is knowing the results of conventional treatment and HSCT in our reality.

As therapeutic advances arise, some diseases for which HSCT was once indicated may evolve to more appropriate approaches, such as the use of imatinib mesylate (Glivec^®^) in the treatment of chronic myeloid leukemia. However, diseases for which HSCT was not even considered are now managed with this procedure, such as sickle cell anemia and deposit diseases.

In pediatrics, the most common indications of allogeneic transplantation are acute lymphocytic leukemia in second bone marrow remission, acute myeloid leukemia, myelodysplastic syndromes, bone marrow failure syndromes, such as severe bone marrow aplasia and Fanconi anemia, immunodeficiencies, some cases of hemoglobinopathies and hereditary metabolic diseases.

In Brazil, diseases that can be treated with transplantation under the Unified Health System (SUS - *Sistema Único de Saúde*) are listed in ordinance 940 of December 21, 2006 (Table 3), and have not been revised as of 2020.^(8)^

**Table 3:** Criteria for hematopoietic stem cell transplantation indication in pediatric diseases

Diagnosis		ICD	Authorized transplants	
Chronic acquired pure red cell aplasia		D60.0	Allogeneic related	BM or UCB
			Allogeneic unrelated	BM or UCB
Aplastic anemia		D61.0	Allogeneic related	BM or UCB
		61.3	Allogeneic unrelated	BM or UCB
Immunodeficiencies		D80.0 - 83.2	Allogeneic related	BM or UCB
			Allogeneic unrelated	BM, PB or UCB
Beta thalassemia – *major*		D56.1	Allogeneic related	BM, PB or UCB
Acute lymphocytic leukemia		C91.0	Allogeneic related	BM, PB or UCB
			Allogeneic unrelated	BM, PB or UCB
Lymphoblastic lymphoma		C83.5	Allogeneic related	BM, PB or UCB
			Allogeneic unrelated	BM, PB or UCB
Acute myeloid leukemia		C92.0	Allogeneic related	BM, PB or UCB
			Allogeneic unrelated	BM, PB or UCB
			Autologous	BM or PB
Chronic myeloid leukemia		C92.1	Allogeneic related	BM or PB
			Allogeneic unrelated	BM or PB
Non-Hodgkin lymphoma		C83.2 - 83.7	Autologous	BM or PB
Hodgkin lymphoma		C81.0 - 81.3	Autologous	BM or PB
			Allogeneic related	BM, PB or UCB
Myelodysplastic syndrome		D46.2 - 42.3	Allogeneic related	BM, PB or UCB
Germ cell tumor				
	Mediastinum	C38.1 - 38.3	Autologous	BM or PB
	Retroperitoneum	C48.8		
	Ovary	C56.0		
	Testis	C62.0 - 62.1		

Source: Brasil. Ministério da Saúde. Secretaria de Atenção à Saúde. Portaria SAS nº. 940 de 21 de dezembro de 2006. Altera o atributo NOME dos procedimentos referentes a TCTH. Brasília (DF): Diário Oficial do Brasil; 2006 Dez de 21[cited 2020 Mar 10]. Available at: http://www.normasbrasil.com.br/norma/portaria-940-2006_197770.html.^(8)^

ICD: International Classification of Diseases and Health-related Problems; BM: bone marrow; UCB: Umbilical cord blood; PB: Peripheral blood.

Survival after BMT for cancer treatment depends on several factors, such as underlying disease, prior treatment, duration of disease, age group and number of recurrences. Overall, 50% to 60% of children are cured, depending on the stage of the disease at which the transplant is performed.

Currently, the greatest challenge is to increase the chance of curing children. To do this, the toxicity of transplants must be reduced, and their results improved. In addition, it is essential to know the treatment reality of our patients, with and without transplantation, allowing for better selection of HSCT candidates.

### Importance of nutritional status in hematopoietic stem cell transplantation

It is a common situation for patients to be hospitalized in good nutritional status and present with significant impairment over the course of HSCT.^(9)^ Both the toxicity and potential complications of this therapy lead to a reduction in food acceptance and/or impairment of adequate nutrient absorption. This situation is even more alarming in children and adolescents, who are in the process of growing and developing. Patients undergoing TBI have more height impairment compared with chemotherapy-only conditioning regimens.^(10)^

The relationship between nutritional status and transplant outcomes has been explored in the last decade with publications initially in adult patients reporting, for example, lower overall survival and higher toxicity, risk of acute GVHD, length of hospital stay and time for engraftment of platelets and neutrophils in malnourished patients or those who had severe weight loss during transplantation.^(11–13)^ Although it is not possible to extrapolate all these results to pediatrics, effects of this impaired nutritional status are believed to have an even greater impact on children and adolescents, not only due to sequelae affecting growth and development, but also due to the lower body mass and what the loss of body mass represents in this age group.

The presence of malnutrition in pediatric HSCT is associated with lower overall survival and higher relapse-free mortality, as well as greater risk of malignancy recurrence.^(9,14)^ In addition, there was a greater presence of post-transplant weakness and a higher risk of chronic GVHD.^(9,15)^ Patients in this nutritional condition also seem to take longer to restore the weight and lean mass lost during HSCT.^(16)^

Similarly, weight loss during transplantation is associated with significant risks to pediatric patients, such as higher prevalence of multi-organ GVHD and presence of lower standardized phase angle, measured by bioimpedance.^(16,17)^ This latter parameter is related to higher mortality and increased risk of chronic GVHD.

Although, for the time being, there are few publications, overweight and obese children and adolescents have been recently studied. Similarly to malnourished patients, overweight patients also have a higher prevalence of post-transplant weakness, lower overall survival and a potentially increased risk for cGVHD.^(15,18,19)^

Malnutrition as a whole, including both undernutrition and overweight/obesity, as well as severe weight loss, can cause dramatic consequences to children and adolescents undergoing HSCT. Current evidence suggests that nutritional follow-up of these patients is key to the success of the proposed therapy.

### Nutritional evaluation

Several factors determine HSCT success in the short and long term. They include diagnosis and disease stage, type of transplantation performed, presence of donor incompatibility, conditioning regimen, cell source, age, prior treatments and nutritional status.^(20)^ For the latter, all patients eligible for HSCT are considered to be at nutritional risk. In order to reduce the negative impact of the disease and treatment, adequate nutritional evaluation is recommended both in pre- and post-transplant cases.^(21)^ Therefore, this evaluation must include: nutritional history, with clinical, nutritional and socioeconomic information; description of anthropometric data; complete physical examination; investigation of the eating patterns; and laboratory tests, particularly blood test and biochemistry.

### Anthropometry

Anthropometry has been widely used for assessment of nutritional status in the pediatric population because it is a practical, non-invasive and low-cost method. The commonly used measurements are weight, height and body mass index (BMI).^(22)^

The classification is based on the parameters recommended by the World Health Organization (WHO) in 2006 and 2007. Weight and height data are reviewed according to age and sex, and classified according to the z-score: weight/age (W/A), height or length/age (H/A), weight/height (W/H) and BMI/age (BMI/A). In addition to the individual review of each parameter, according to tables 4 and 5, the final classification is according to the age group: under 2 years of age, W/H z-score and, over 2 years of age, BMI/A. The patient’s evolution must also be followed based on growth charts according to the parameter, sex and age.^(23–26)^

**Table 4:** Anthropometric index determining nutritional status by age group, zero to 10 years

Critical values	Anthropometric indices						
	Children aged 0 to <5 years				Children aged 5 to <10 years		
	W/A	W/H	BMI/A	H/A	W/A	BMI/A	H/A
< Z-score -3	Very low weight for age	Marked thinness	Marked thinness	Very low height for age	Very low weight for age	Marked thinness	Very low height for age
> Z-score -3 and < z-score -2	Low weight for age	Thinness	Thinness	Low height for age	Low weight for age	Thinness	Low height for age
> Z-score -2 and < z-score -1	Adequate weight for age	Eutrophy	Eutrophy	Adequate height for age	Adequate weight for age	Eutrophy	Adequate height for age
> Z-score -1 and < z-score +1							
> Z-score +1 and < z-score +2		Risk of overweight	Risk of overweight			Overweight	
> Z-score + 2 and < z-score +3	High weight for age	Overweight	Overweight		High weight for age	Obesity	
> Z-score +3		Obesity	Obesity			Severe obesity	

Source: Sociedade Brasileira de Pediatria (SBP). Avaliação nutricional da criança e do adolescente: manual de orientação. Departamento de Nutrologia. Rio de Janeiro: SBP; 2009. Capítulo 5. Avaliação da composição corporal. p.46-51;^(23)^ Silva AP, Nascimento AG. Zamberlan P. Manual de dietas e condutas nutricionais em pediatria. São Paulo: Atheneu; 2014. p. 21-6.^(24)^

W/A: weight/age; W/H: weight/height; BMI/A: body mass index/age; H/A: height/age.

**Table 5:** Anthropometric index determining nutritional status by age group, 10 to 19 years

Critical values	Anthropometric indices for adolescents	
	BMI/A	H/A
< Z-score -3	Marked thinness	Very low height for age
> Z-score -3 and < z-score -2	Thinness	Low height for age
> Z-score -2 and > z-score -1	Eutrophy	Adequate height for age
> Z-score -1 and < z-score +1		
> Z-score +1 and < z-score +2	Overweight	
> Z-score +2 and < z-score +3	Obesity	
> Z-score +3	Severe obesity	

Source: Sociedade Brasileira de Pediatria (SBP). Avaliação nutricional da criança e do adolescente: manual de orientação. Departamento de Nutrologia. Rio de Janeiro: SBP; 2009. Capítulo 5. Avaliação da composição corporal. p.46-51;^(23)^ Silva AP, Nascimento AG. Zamberlan P. Manual de dietas e condutas nutricionais em pediatria. São Paulo: Atheneu; 2014. p. 21-6.^(24)^

BMI/A: body mass index/age; H/A: height/age.

### Body composition

Assessment of body composition as one of the tools of nutritional therapy has become increasingly valued in recent years, since calculating weight or BMI for age is not enough to evaluate fat and lean mass in children and adolescents with chronic diseases.^(27–31)^ Furthermore, these indicators are not much sensitive to variations in nutritional status, since body weight is affected by significant water changes caused by HSCT. There are several methods to assess body composition, such as waist circumference and skin folds, magnetic resonance, computed tomography, body densitometry, ultrasound, bioimpedance analysis, total body potassium and air plethysmography.^(29–31)^

In pediatrics, and in association with allogeneic HSCT, many studies use body densitometry for this evaluation, which is not performed in most Brazilian services due to its high cost and/or unavailability.^(31,32)^ Both obesity and undernutrition are risk factors in this procedure, with highlight to lower muscle mass and higher peripheral and visceral fat.^(18,31–35)^

Triceps skinfold (TSF) and subscapular skinfold are the most commonly used in pediatrics because they have population reference values for this age group.^(25,36,37)^ When these values are below the 5th percentile or above the 90th percentile, there are health risks. TSF measurement is a convenient way to indirectly establish body fat mass.^(38,39)^ Arm and arm muscle circumference are primarily used to obtain the amount and variation of skeletal muscle protein. One should remember that, under anasarca, the arm circumference and TSF have limited applicability.

Despite the lack of publications on children during HSCT, some current studies point to the use of TSF, arm circumference and arm muscle area as the best indicators of body composition in pediatric cancer patients, since they are easy to measure, low-cost and non-invasive, having good correlation with other parameters considered gold standard for body composition in this population. Studies have recommended measuring arm anthropometry based on comparison with weight indices and non-referenced malnutrition measurements.^(22,39–43)^

High-dose cranial irradiation used in the TBI can cause hypothalamus abnormalities, including changes in growth hormone, thyroid and gonadal functions, as well as abnormal sensitivity to leptin, ghrelin and insulin.^(31)^ These changes are associated with obesity, particularly post-HSCT, including increased visceral fat and fatty infiltration of the liver.^(31,33)^ Patients who receive TBI for conditioning, when compared with those receiving only chemotherapy or obese controls, have lower lean mass, with a higher prevalence of lean mass loss and more visceral and intramuscular fat.^(33)^

In addition to irradiation, GVHD, one of the most common complications of HSCT, correlates to changes in body composition.^(31)^ In intestinal GVHD, weight loss is greater, with decrease of muscle mass – in most cases, without increase in visceral fat.^(31)^ The use of corticosteroids in the management of GVHD contributes to increasing visceral and peripheral fat, as well as reducing lean mass in these patients.^(18,32)^

In HSCT survivors, the higher propensity to obesity and decreased lean mass is related to a higher risk of cardiovascular disease, metabolic syndrome, growth and bone deficiency, leading to higher morbidity and mortality in these children.^(31,32,34)^

Allogeneic HSCT, TBI, use of corticosteroids and GVHD are important risk factors for body composition changes in survivors, associated with increased fat mass and loss of muscle mass.^(18,31,32,34–36)^ Patients with these factors should have their body composition closely monitored as an early therapeutic measure, to reduce the risk of morbidity and mortality.^(31,32,35)^ The phase angle measured by bioimpedance seems promising as a proxy measure of nutritional status, and may be an option in the follow-up of these patients. In a Brazilian study published in 2013, children with lower phase angle had severe weight loss, higher incidence of GVHD and lower survival.^(9)^

Although in other fields of nutrition the evaluation of body composition is included in institutional protocols, in most Brazilian pediatric HSCT services, there is no consistency. It is advised that this evaluation be carried out at all stages of HSCT, before, during and after, to improve the survival and quality of life of these patients. Each service must choose the method that has the best cost-benefit and convenience among those validated.

### Laboratory parameters

Laboratory parameters are important when evaluating nutritional status, and provide clear measures of abnormalities, with the advantage of enabling follow-up over time and early interventions.^(43)^ In addition, they may assist in nutritional monitoring and decision-making regarding specific nutritional therapies. Thus, it is possible to divide laboratory parameters into two large groups: nutritional status and nutritional therapy monitoring.

#### Nutritional status

It should be borne in mind that, although different biochemical tests can offer important information regarding nutritional status, none of them should be used in isolation, since changes may occur during the clinical evolution of the patient. Among the biochemical variables of interest in nutrition, regarding the nutritional status of patients undergoing HSCT, we highlight:

- Albumin: considered the main protein synthesized by the liver and, consequently, the most widely used to evaluate nutritional status. Serum albumin concentrations lower than 3g/dL are indicative of protein malnutrition. This decrease also correlates with an increased incidence of clinical complications, morbidity and mortality. In severe patients, albumin levels have prognostic value when measured on admission to the hospital. However, serum albumin is not sensitive to changes during acute complications, since its half-life is relatively long (±20 days), and it may take several weeks to respond to variations in dietary-protein intake.^(44–46)^ During HSCT, weekly albumin testing is recommended. Values lower than 2.5mg/dL are potentially associated with intravascular volume overload, systemic capillary extravasation syndrome, diarrhea or liver diseases.^(47)^
- Pre-albumin: in serum, prealbumin carries about 70% of thyroxin, and is also the carrier of retinol-binding protein (RBP), both of which are decreased in energy-protein malnutrition, as well as in infections, cirrhosis, hepatitis, inflammation, stress and chronic diseases. As a marker of metabolic stress, it can help determine the need for nutritional intervention. Both pre-albumin and RBP have short half-lives, and are considered important for evaluating visceral protein status. Retinol binding protein has a half-life of only a few hours (±12 hours) and pre-albumin (PA), of 2 days. Due to their short half-lives, they are considered some of the most sensitive parameters to nutritional changes.^(48)^ Levels <10mg/dL are considered abnormal in children.^(47)^
- Transferrin: beta-globulin synthesized in the liver and responsible for transporting certain nutrients, particularly iron. It has an average life of 4 to 8 days, and thus abnormal levels can be detected much sooner than for albumin, in case of protein depletion, and this makes it an important tool for diagnosing subclinical malnutrition. However, it has low sensitivity and specificity when used in isolation, with its levels increasing in iron deficiency anemia and decreasing in liver diseases, sepsis, malabsorption and inflammatory changes.^(47,48)^ In patients eligible for HSCT, multiple blood transfusions prevent transferrin from being a good evaluation parameter. Values between 200 and 400mg/dL are considered normal. Values below 200mg/dL are considered indicative of mild to moderate protein deficiency, while levels below 100mg/dL suggest severe deficiency. One should avoid using transferrin to evaluate protein depletion in patients with iron deficiency anemia, since in these cases, its levels are increased, reflecting the increase in iron transport.^(49)^

The reference values for hepatic proteins (albumin, prealbumin and transferrin) are shown in table 6.

**Table 6:** Reference values for hepatic proteins

Serum protein	Reference values	Function
Albumin, g/dL	Normal: >3.5	Majorly responsible for colloid osmotic pressure
	Mild depletion: 3.0-3.5	
	Moderate depletion: 2.5-2.9	
	Severe depletion: <2.5	
Prealbumin, mg/dL	Normal: 20	Transporting thyroid hormones (thyroxin)
	Mild depletion: 10-15	
	Moderate depletion: 5-10	
	Severe depletion: <5	
Transferrin, mg/dL	Normal: 200-400	Transporting certain nutrients, particularly iron
	Mild depletion: 150-120	
	Moderate depletion: 100-150	
	Severe depletion: <100	

Source: Pedrón Giner C, Cuervas-Mons Vendrell M, Galera Martínez R, Gómez López L, Gomis Muñoz P, Irastorza Terradillos I, Martínez Costa C, Moreno Villares JM, Pérez-Portabella Maristany C, Pozas Del Río MᵃT, Redecillas Ferreiro SE, Prieto Bozano G; Grupo de Estandarización de la Senpe S. Pediatric parenteral nutrition: clinical practice guidelines from the Spanish Society of Parenteral and Enteral Nutrition (SENPE), the Spanish Society of Pediatric Gastroenterology, Hepatology and Nutrition (SEGHNP) and the Spanish Society of Hospital Pharmacy (SEFH). Nutr Hosp. 2017;34(3):745-58;^(50)^ Bottoni A, Oliveira GP, Ferrini MT, Waitzberg DL. Avaliação nutricional: exames laboratoriais. In: Waitzberg DL. Nutrição oral, enteral e parenteral na prática clínica. 3a ed. São Paulo: Atheneu; 2001. v. 1. p. 279-94.^(51)^

However, it is important to bear in mind that these proteins have a limited role in severe patients, due to hemodilution and liver function changes for production of acute phase proteins decreasing visceral protein levels. As a result of these changes, lower values in these patients reflect more the severity of the disease than the nutritional status.^(52)^ It is important to note that some authors have correlated transferrin and prealbumin changes to GVHD.^(53)^

- Creatinine height index (CHI): an appropriate parameter to evaluate body muscle mass based on the fact that 98% of creatinine is stored in the muscles.^(54)^ It is used to evaluate muscle mass and indirectly assess nitrogen balance. Malnourished patients present with severe skeletal muscle degradation, which can be estimated from urinary creatinine levels, a metabolite of the breakdown of creatinine, which is constantly synthetized. It is, therefore, a marker of muscular catabolism.^(42)^ The CHI is calculated as a percentage of a standard, by dividing urinary creatinine excretion by a standard value for the patient’s height. A normal CHI is around 1. Values lower than 60% of the standard detect patients at increased risk for malnutrition.^(46)^ This assessment has some disadvantages, such as being reliable only in patients with normal kidney function, the need for 24-hour urine sampling, interference of age and dietary protein content, among others.^(55)^
- Nitrogen balance: this is a useful method for evaluating protein status. It allows to determine the level of balance between nitrogen intake and urinary nitrogen excretion. When the intake is sufficient to cover losses, the balance is positive. If, on the contrary, losses exceed gains, the balance is negative. It is good for estimating protein intake and degradation and therefore, an important tool in the follow-up and treatment monitoring of transplanted patients.^(56,57)^ Some authors consider that nitrogen balance should not be used as a malnutrition parameter in early treatment phases, due to the impossibility of a positive balance being achieved at this stage, even in patients with adequate nutritional support.^(44)^
- Cholesterol: hypocholesterolemia (under 160mg/dL) has been associated with malnutrition and, consequently, increased mortality in critical patients. However, low serum cholesterol levels are also observed in kidney and liver failure, as well as malabsorption. Increased cholesterol levels are known to be a risk factor for coronary disease.^(44,57)^

### Nutritional therapy algorithms

Some routine examinations in HSCT patients help in decision-making regarding the best nutritional therapy. Although these parameters can be affected by bone marrow adaptation, they are used as a complement for defining the nutritional approach. Their applicability during HSCT is shown in table 7.^(20,22)^

**Table 7:** Laboratory parameters their applicability in nutritional therapy during hematopoietic stem cell transplantation

Laboratory parameters		Applicability in HSCT
Complete blood count	Hemoglobin	Important in evaluating anemia
		May be abnormal in HSCT
		It is not a good biochemistry parameter for children undergoing HSCT, if used in isolation
		Must be monitored to optimize high-protein food supply
	Leukocytes	Defense cells of the body
		Abnormal until total recovery of the bone marrow
		Indicator for introduction of dietary restrictions for potentially contaminated foods (*e.g.* no raw food) when counts are below 1.500/mm³
	Neutrophils	Primary defense cells, mainly in bacterial infections
		Abnormal until full blood marrow recovery or during drug treatment
		Indicator for introduction of dietary restrictions for potentially contaminated foods (*e.g.*, no raw food) when counts are below 500/mm³
	Platelets	Takes part in the blood coagulation process
		Abnormal until total recovery of the bone marrow
		When below the reference value, caution is advised when indicating urinary catheter due to the risk of bleeding, and when assessing body composition
Liver function	Transaminases	Liver function test parameter
		Usually abnormal during HSCT
		Monitor variations and, if necessary, control consumption of fat-rich foods and preparations
	Bilirubin	Indicator of liver abnormalities
		If increased, it can be indicative of hepatocellular damage
		Monitor variations and, if necessary, control consumption of fat-rich foods and preparations
Kidney function	Creatinine	Indicator of kidney function abnormalities
		May be increased in situations of prolonged hydration deprivation
		Monitor tests and optimize water supply
	Urea	May be an indicator of hydration status
		Increased in cases of dehydration or excessive protein catabolism and decreased in hyper-hydration or malnutrition
		Monitor tests and water supply
	Sodium	If increased, it is suggestive of low water intake or dehydration and, if decreased, it may be suggestive of edema
		Follow up hydration tests and perform anthropometric assessment
	Potassium	Assists in evaluating kidney function
		Abnormal values can require optimization or restriction of potassium-rich food consumption
Lipid profile	Total cholesterol	Can be abnormal due to drugs or changes in dietary intake
		Monitor tests and adapt diet
	Triglycerides	May be abnormal after changes in eating habits or use of drugs. For patients on parenteral therapy, it can be used to evaluate lipid supply and to assess the onset and progression of infusion rates
Blood glucose		May be abnormal if corticosteroids are used, leading to hyperglycemia
		Patients may present with hypoglycemia after long fasting periods
		Perform longitudinal follow-up and limit refined sugar in diet

HSCT: hematopoietic stem cell transplantation.

The assessment of nutritional status using biochemistry parameters allows for early diagnosis of nutritional problems, even before anthropometric changes or onset of clinical signs and symptoms, making them an important tool in the follow-up of these patients, combined with other evaluation parameters.^(52)^

### Physical examination

Physical examination in pediatrics is an indicator of several pediatric diseases. Despite the potential biases due to the HSCT-related toxicity, the physical examination is recommended as part of the nutritional evaluation, in order to identify clinical signs of malnutrition prior to or resulting from treatment.^(23)^

Table 8 shows the main clinical signs that should be observed in the physical examination, and to which diagnosis they may be related in the pediatric age group.^(23)^

**Table 8:** Clinical signs and diagnoses in pediatric patients

Area	Clinical signs	Diagnosis
Hair	Loss of natural shine	Kwashiorkor and, less frequently, marasmus
	Thin and sparse	
	Brittle hair	
	Depigmented	
	Loose	
	Flag sign	
Face	Nasolabial seborrhea (dry skin around nostrils)	Riboflavin deficiency
	Swollen face (moon face)	Kwashiorkor
	Pallor	Iron deficiency
Eyes	Conjunctival pallor	Anemia
	Red membranes	
	Bitot’s spots	Vitamin A deficiency
	Conjunctival xerosis	
	Corneal xerosis	
	Keratomalacia	
	Red and cracked skin around the eyes	Riboflavin and pyridoxine
	Corneal arcus (white arc around eyes)	Hyperlipidemia
	Xanthelasma (bumpy yellow patches around the eyes)	
Lips	Angular stomatitis (pink and white lesions around the mouth)	Riboflavin
	Angular cheilitis	
	Cheilosis (red, swollen lips)	
Tongue	Scarlet, inflamed tongue	Nicotinic acid
	Magenta tongue (purplish red)	Riboflavin
	Swollen tongue	Niacin
	Atrophy and hypertrophy of the filiform papilla	Folic acid and vitamin B12
Teeth	Tooth enamel stains	Fluorine
Gingiva	Spongy: bleeding and oozing	Vitamin C
Glands	Enlarged thyroid	Iodine
	Enlarged parathyroid	Starvation
Skin	Xerosis	Vitamin A
	Follicular hyperkeratosis (sandpaper skin)	
	Petechias	Vitamin C
	Dermatosis, pellagra	
	Easy bruising	Vitamin K
	Desquamative cosmetic dermatosis	Kwashiorkor
	Vulvar and scrotal dermatosis	Riboflavin
	Xanthomas	Hyperlipidemia
Nails	Spoon-shaped, brittle and rough	Iron
	Small white spots	Zinc

Source: Sociedade Brasileira de Pediatria (SBP). Avaliação nutricional da criança e do adolescente: manual de orientação. Departamento de Nutrologia. Rio de Janeiro:SBP; 2009. Capítulo 5. Avaliação da composição corporal. p.46-51.^(23)^

### Food intake

Nutritional assistance in HSCT must take into account increased energy and nutrient requirements, as well as dietary restrictions for a frail patient, who can often go on to having an unhealthy diet, when it comes to preventing chronic diseases.^(58)^

After HSCT, the diet has particular characteristics, and health-related aspects are prioritized at this stage over nutritional aspects.^(59,60)^ The non-consumption of raw foods and other restrictions in food choices lead to dietary deficiencies that must be compensated with dietary supplements and/or drugs. In the post-HSCT phase, it is common to see an increase in intake of low nutritional quality foods, such as unhealthy snacks and fried food. In this sense, the diet does not comply with qualitative recommendations for the prevention of chronic diseases.^(58)^ Since nutritional requirements after transplantation tend to increase, patients often choose foods that are not so healthy but have a role in weight preservation.^(58)^

Food intake is assessed by means of a 24-hour food journal for children over 8 years of age and, for children under 8, this requires support from parents or guardians. It aims to evaluate the qualitative and quantitative intake of calories, micronutrients and macronutrients^(58,61)^ appropriate to the age group, according to tables 9 to 11.

**Table 9:** Feeding schedule for breastfed infants (aged zero to 12 months)

Meal	Age (months)			
	6-7	7-8	8-10	12
Breakfast	Breast milk	Breast milk	Breast milk	Breast milk or infant formula
				Bread or biscuit
Break	Pureed fruit	Pureed fruit	Pureed fruit	Pureed fruit
Lunch	Breast milk	Food puree	Food puree	Food puree or family meal
Snack	Pureed fruit	Pureed fruit	Pureed fruit	Pureed fruit
	Breast milk	Breast milk	Breast milk	Breast milk or age-appropriate formula
				Bread or biscuit
Dinner	Breast milk	Breast milk	Food puree	Food puree or family meal
Night	Breast milk	Breast milk	Breast milk	Breast milk or age-appropriate formula

Source: Sociedade Brasileira de Pediatria (SBP). Avaliação nutricional da criança e do adolescente: manual de orientação. Departamento de Nutrologia. Rio de Janeiro: SBP; 2009. Capítulo 5. Avaliação da composição corporal. p. 46-51;^(23)^ Silva AP, Nascimento AG. Zamberlan P. Manual de dietas e condutas nutricionais em pediatria. São Paulo: Atheneu; 2014. p. 21-6.^(24)^

**Table 10:** Feeding schedule for formula-fed infants (aged 0 to 12 months) (age-appropriate formula/milk)

Meal	Age (months)			
	6-7	7-8	8-10	12
Breakfast	Age-appropriate formula	Age-appropriate formula	Age-appropriate formula	Age-appropriate formula/milk
				Bread or biscuit
Break	Pureed fruit	Pureed fruit	Pureed fruit	Pureed fruit
Lunch	Age-appropriate formula	Food puree	Food puree	Food puree or family meal
Snack	Pureed fruit	Age-appropriate formula	Pureed fruit	Pureed fruit
	Age-appropriate formula		Age-appropriate formula	Age-appropriate formula/milk
				Bread or biscuit
Dinner	Age-appropriate formula	Age-appropriate formula	Food puree	Food puree or family meal
Night	Age-appropriate formula	Age-appropriate formula	Age-appropriate formula	Age-appropriate formula/milk

Source: Sociedade Brasileira de Pediatria (SBP). Avaliação nutricional da criança e do adolescente: manual de orientação. Departamento de Nutrologia. Rio de Janeiro: SBP; 2009. Capítulo 5. Avaliação da composição corporal. p.46-51;^(23)^ Silva AP, Nascimento AG. Zamberlan P. Manual de dietas e condutas nutricionais em pediatria. São Paulo: Atheneu; 2014. p. 21-6.^(24)^

**Table 11:** Feeding schedule for preschool-age children (1 to 6 years), school-age children (7 to 12 years) and adolescents (over 12 years)

Food group	Portion	
	Preschool and school-aged children	Adolescents
Cereals, breads, tubers, root vegetables and pasta (preferably wholegrain)	5	5-9
Leafy vegetables and legumes	3	4-5
Fruits	3	4-5
Milk and dairy products	3	3
Meats or eggs	2	1-2
Pulses (beans, lentils, chickpeas and peas)	1	1
Oils and fats	1	1-2
Sugars and sweets	1	1-2

Source: Sociedade Brasileira de Pediatria (SBP). Avaliação nutricional da criança e do adolescente: manual de orientação. Departamento de Nutrologia. Rio de Janeiro: SBP; 2009. Capítulo 5. Avaliação da composição corporal. p.46-51;^(23)^ Silva AP, Nascimento AG. Zamberlan P. Manual de dietas e condutas nutricionais em pediatria. São Paulo: Atheneu; 2014. p. 21-6.^(24)^

### Nutritional requirements

Energy and nutrient recommendations specific to children change according to the age. Since energy should be directed toward maintaining metabolic needs as well as growth and development, energy and nutrient requirements in children and adolescents are increased, placing them at high nutritional risk.^(62–66)^

During the transplant period, many factors can change nutritional requirements, such as a high-dose chemotherapy conditioning regimen, whether or not combined with TBI, mucositis, lack of appetite, nausea, vomiting, diarrhea or anorexia. These manifestations have negative implications for calorie and protein supply as well as nutrient absorption, in addition to leading to increased energy requirements or protein catabolism.^(63,64,67–72)^ In general, patients undergoing allogeneic HSCT are usually more immunocompromised and at higher risk for general complications when compared with autologous HSCT.^(63)^ These very aggressive changes that cause metabolic and nutritional abnormalities.^(71,73)^

The reduced energy and protein intake may influence immune function during metabolic stress. Therefore, meeting energy and protein requirements is of extreme importance in order to maintain an adequate nitrogen balance.^(74)^ Adequate growth and development in children and adolescents depend on a balanced diet, with satisfactory supply of energy, macro and micronutrients.^(69,74)^

After discharge, patients may have several nutrition-related problems, such as malnutrition due to insufficient oral intake or metabolic disorders. Nutritional support plays an important role in the different types of HSCT.^(75)^

In sum, adequate nutritional support before and after transplantation is a potentially important support measure.^(76)^ The changes in these patients predominantly affect protein, energy and micronutrient metabolism. During HSCT, energy and protein requirements of children and adolescents must be taken into account to ensure that nutritional support is adapted and adequate to the individual needs of children.^(77)^

### Energy requirements

Energy requirements depend on factors such as baseline nutritional status, age, weight and degree of metabolic stress.^(74)^ Although it has been demonstrated that the energy expenditure of patients undergoing HSCT may differ among allogeneic and autologous transplants, there are studies showing that energy requirements of HSCT recipients can reach 130% to 150% of the predicted resting energy expenditure.^(70,73,78,79)^

It is necessary to know the energy requirements of children during HSCT to ensure appropriate nutritional support.^(77)^

Energy recommendations can be determined based on a standard estimation equation and the use of indirect calorimetry, the latter being a more accurate measurement of energy requirements.^(64,65,74)^

Bechard *et al.*,^(80)^ looked at anthropometric data, food intake, caloric requirements based on the Schofield equation, and resting energy expenditure by calorimetry between HSCT D0 and D+30. In this period, resting energy expenditure is reduced, particularly between D0 and D+21, as well as food intake. Despite controversial studies, the authors suggested that, when indirect calorimetry is not available, the basal metabolic rate and a caloric target of 100% to 140% should be used, and excess supply should be avoided.

Energy requirements should be estimated using standard formulas (Table 12) and adjusted based on the clinical evolution.^(65)^

**Table 12:** Summary of energy requirements of pediatric patients undergoing hematopoietic stem cell transplantation.

Pre-HSCT		Conditioning, bone marrow aplasia, bone marrow engraftment (approximately up to D+35)		Post-HSCT, hospital discharge, outpatient follow-up	
1. Holliday and Segar (1957)		1. Scholfield (Murphy, 2004)		1. Holliday and Segar (1957)	
	Children 0-10kg: 100kcal/kg Children 10-20kg: 1,000kcal + 50kcal/kg for every kg over 10kg Children over 20kg: 1,500kcal + 20kcal/kg for every kg over 20kg	Boys			Children 0-10kg: 100kcal/kg Children 10-20kg: 1,000kcal + 50kcal/kg for every kg over 10kg Children over 20kg: 1,500kcal + 20kcal/kg for every kg over 20kg
			<3 years: (0.240 x body weight (kg) – 0.127) x 239 3- 10 years: (0.095 x body weight (kg)+ 2.110) x 239 10- 18 years: (0.074 x body weight (kg)+ 2.7540) x 239 18- 30 years: (0.063 x body weight (kg)+ 2.896) x 239		
		Girls			
			<3 years: (0.244 x body weight (kg) – 0.130) x 239 3- 10 years: (0.085 x body weight (kg)+ 2.033) x 239 10- 18 years: (0.056 x body weight (kg)+ 2.898) x 239 18- 30 years: (0.062 x body weight (kg)+ 2.036) x 239		
2. Aspen (2002)				2. Aspen (2002)	
	0-1 year: 90-120kcal/kg 1-7 years: 75-90kcal/kg 7-12 years: 60-75kcal/kg 12-18 years: 30-60kcal/kg 18-25 years: 25-30kcal/kg				0-1 year: 90-120kcal/kg 1-7 years: 75-90kcal/kg 7-12 years: 60-75kcal/kg 12-18 years: 30-60kcal/kg 18-25 years: 25-30kcal/kg
3. DRIs 2006				3. DRIs 2006	
	0- 3 months: (89 x body weight (kg) -100) + 175 4-6 months: (89 x body weight (kg) -100) + 56 7- 12 months: (89 x body weight (kg) -100) + 22 13-35 months: (89 x body weight (kg) -100) + 20				0- 3 months: (89 x body weight (kg) -100) + 175 4-6 months: (89 x body weight (kg) -100) + 56 7-12 months: (89 x body weight (kg) -100) + 22 13-35 months: (89 x body weight (kg) -100) + 20
Boys				Boys	
	3- 8 years: 88.5 - 61.9 x age + activity factor x (26.7 x body weight (kg) + 903 x height) + 20 9- 18 years: 88.5 – 61.9 x age + activity ator x (26.7 x body weight (kg) + 903 x height) + 25				3- 8 years: 88.5 - 61.9 x age + activity factor x (26.7 x body weight (kg) + 903 x height) + 20 9- 18 years: 88.5 - 61.9 x age + activity factor x (26.7 x body weight (kg) + 903 x height) + 25
Girls				Girls	
	3- 8 years: 135.3 - 30.8 x age + activity factor x (10 x body weight (kg) + 934 x height) + 20 9- 18 years: 135.3 - 30.8 x age + activity factor x (10 x body weight (kg) + 934 x height) + 25				3- 8 years: 135.3 - 30.8 x age + activity factor x (10 x body weight (kg) + 934 x height) + 20 9- 18 years: 135.3 - 30.8 x age + activity factor x (10 x body weight (kg) + 934 x height) + 25
Activity factor				Activity factor	
	1 = activities of daily living Boys = 1.16; Girls = 1.13 – activities of daily living + 30 to 60 minutes of moderate activity Boys = 1.31; Girls = 1.26 – activities of daily living + 60 minutes of moderate activity For children with low weight, use W/H of the 50th percentile or z-score = 0.00 For eutrophic children, use current weight For overweight or obese children, use W/H of the 90th percentile or z-score = +2.00 This adjustment in relation with the current weight should not exceed 20%				1 = activities of daily living Boys = 1.16; Girls = 1.13 – activities of daily living + 30 to 60 minutes of moderate activity Boys = 1.31; Girls = 1.26 – activities of daily living + 60 minutes of moderate activity For children with low weight, use W/H of the 50th percentile or z-score = 0.00 For eutrophic children, use current weight For overweight or obese children, use W/H of the 90th percentile or z-score = +2.00. This adjustment in relation with the current weight should not exceed 20%

Source: Pine M, Wang L, Harrell FE Jr, Calder C, Manes B, Evans M, et al. The effect of obesity on outcome of unrelated cord blood transplant in children with malignant diseases. Bone Marrow Transplant. 2011;46(10):1309-13;^(19)^ White M, Murphy AJ, Hastings Y, Shergold J, Young J, Montgomery C, et al. Nutritional status and energy expenditure in children pre-bone-marrow-transplant. Bone Marrow Transplant. 2005;35(8):775-9;^(77)^ Bechard LJ, Feldmann HA, Venick R, Gura K, Gordon C, Sonis A, et al. Attenuation of restimg energy expenditure following hematopoietic STC in chlidren. Bone Marrow Transplant. 2012;47(10):1301-6.^(80)^

HSCT: hematopoietic stem cell transplantation; DRI: *Dietary Reference Intake;* W/H: weight/ height.

During parenteral nutrition, it is recommended that energy requirements be 80% to 90% of the recommendations for enteral nutrition.^(65)^

### Protein requirements

Protein requirements in pediatric patients are also increased^(59,74)^ and should be qualitatively and quantitatively adequate.^(65)^ They vary according to age and weight.^(57)^ These additional quantities of protein are intended to restore or preserve body lean mass and provide substrate for the hypercatabolic state after HSCT. It is worth noting that, in case of impaired kidney or liver function, these requirements are modified and must be adjusted individually.^(74)^

Reduced protein supply can negatively affect immune function during metabolic stress. Therefore, it is important to adapt protein requirements for a zero nitrogen balance.^(63)^ Nutritional support that is adequate to prevent protein loss, without providing too many calories and promoting fat accumulation, is essential for the survival of patients undergoing transplantation.^(64)^

Protein requirements are estimated (Table 13) in order to provide substrate for tissue repair after cytoreduction and decrease lean mass loss after HSCT. Overall, values range from 1.4g/kg to 3.0g/kg body weight.^(71,73)^

**Table 13:** Summary of energy requirements in pediatric patients undergoing hematopoietic stem cell transplantation

Pre-HSCT, post-HSCT, hospital discharge, outpatient follow-up	Conditioning, bone marrow aplasia, bone marrow engraftment (approximately up to D+35)	
Age-based	Age-based*	
From neonates up to 2 years old: 2.5-3.0g/kg/day		0- 6 years: 2.5-3.0g/kg of current weight
Children aged 2 to 11 years: 2.0g/kg/day		7- 10 years: 2.4g/kg of current weight
Adolescents (over 12 years): 1.5-2.0g/kg/day		11- 14 years: 2.0g/kg of current weight
In cases of weight loss and malnutrition, a 15% to 50% increment in protein recommendations is suggested		15- 18 years: 1.8g/kg of current weight
For children with low weight, use W/H of the 50th percentile or z-score = 0.00		
For eutrophic children, use current weight		
For overweight or obese children, use W/H of the 90th percentile or z-score = +2.00		
This adjustment in relation with the current weight should not exceed 20%		

Source: Brasil. Ministério da Saúde. Instituto Nacional de Câncer José Alencar Gomes da Silva (INCA). Consenso nacional de nutrição oncológica. 2a ed. rev. ampl. atual. Rio de Janeiro: INCA; 2015. p.182 [cited 2020 Jul 4]. Available at: https://www.inca.gov.br/sites/ufu.sti.inca.local/files/media/document/consenso-nacional-de-nutricao-oncologica-2-edicao-2015.pdf;^(20)^ Mendes TG, Benedetti FJ. Fatores nutricionais associados ao câncer em crianças e adolescentes. Disciplinarum Scientia. 2013;14(2):265-72;^(61)^ Sociedade Brasileira de Nutrição Parenteral e Enteral. Associação Brasileira de Cirurgia Pediátrica. Sociedade Brasileira de Clínica Médica. Associação Brasileira de Nutrologia. Projeto Diretrizes. Recomendações nutricionais para crianças em terapia nutricional enteral e parenteral. São Paulo: AMBCFM; 2011. p. 1-16 [cited 2019 Dec 10]. Available at: https://diretrizes.amb.org.br/_BibliotecaAntiga/recomendacoes_nutricionais_para_criancas_em_terapia_nutricional_enteral_e_parenteral.pdf.^(65)^

* In case of impaired kidney or liver function, these requirements are modified and must be adjusted individually.

HSCT: hematopoietic stem cell transplantation; W/H: weight/height.

### Micronutrient requirements

Vitamins are considered important for growth and vary according to age.^(65)^

Levels of vitamins, both water-soluble and fat-soluble, are abnormal in patients undergoing HSCT, as a result of low intake or malabsorption. Other factors also play a role, such as cyclosporine and radiotherapy, which lead to increased vitamin requirements.^(71,75)^

For all patients not receiving parenteral nutrition, it is advised to offer oral multivitamin and mineral supplements, due to their limited intake regular foods, often for a prolonged period.^(81)^

Some micronutrients are particularly relevant in HSCT:

- Vitamin D or cholecalciferol: Vitamin D deficiencies are prevalent in patients with GVHD. This vitamin has an endocrine role as a key regulator of calcium absorption and bone homeostasis and also immune system regulation. It is required for development and maintenance of bone tissue and maintenance of calcium and phosphorus homeostasis. Without vitamin D, only 10% to 15% of the calcium ingested and approximately 60% of the phosphorus would be absorbed.^(71,73)^ Patients undergoing HSCT present with vitamin D insufficiency or deficiency, possibly due to post-transplant decreased food intake and avoiding sun exposure.^(71,73)^
- Vitamin K: low levels in HSCT increase the risk of severe hemorrhage. The etiology of vitamin K deficiency is often multifactorial and often results from drug antagonism, liver dysfunction, malabsorption, anorexia and/or inadequate intake of nutrients.^(82)^
- Zinc: Zinc deficiency is prevalent in patients after allogeneic HSCT, particularly in those with diarrhea. Zinc is necessary for good genetic functioning, immunity, red blood cell formation, organs, muscle and bone function, and cell membrane stability, in addition to cell growth, division and genetic differentiation. It also plays an important role in the context of metabolic response to injury and wound healing.^(83)^ Zinc sulphate is considered a promising agent for prevention of mucositis, which occurs during HSCT, for being an essential cofactor in several cellular processes, such as DNA, RNA polymerase and reverse transcriptase synthesis, and having an effect on wound healing, growth and immunity.^(83)^ Clinical manifestations of zinc deficiency, such as anorexia, rash, diarrhea and infections, are also common.^(69,71)^
- Ferritin: an intracellular iron storage and transport protein, directly related with proper intake of this micronutrient, and found in virtually all cells of the body, mainly in hepatocytes and body fluids. In plasma, it is present in small concentrations and correlates with iron stocks in the body. Its primary function is to accumulate intracellular iron, which, in its biologically available form, is vital for cellular processes, protecting proteins, lipids and DNA, in addition to playing an important role in inflammatory conditions.^(71)^

Micronutrient needs for HSCT patients are not fully established, and usually, the Dietary Reference Intake (DRI) recommendations for healthy populations should be followed, but some items are worth highlighting, as shown in table 14.

**Table 14:** Recommended differential intake of some micronutrients for children during hematopoietic stem cell transplantation

Group	Vitamin D		Vitamin K	Zinc			Iron		
	Proper intake	Maximum tolerated intake	Proper intake	Estimated average requirement	Proper intake	Maximum tolerated intake	Estimated average requirement	Proper intake	Maximum tolerated intake
0-6 months	5	25	2.0		2	4	ND	0.27	40
7-12 months	5	25	2.5	2.2	3	5	6.9	11	40
1- 3 years	5	50	30	2.2	3	7	3	7	40
4- 8 years	5	50	55	4.0	5	12	4.1	10	40
9- 13 years	5	50	60	7.0	8	23	5.9	8	40
14- 18 years	5	50	75	8.5	11	34	7.7	11	45
19- 30 years	5	50	120				6	8	45

Source: Sociedade Brasileira de Nutrição Parenteral e Enteral. Associação Brasileira de Cirurgia Pediátrica. Sociedade Brasileira de Clínica Médica. Associação Brasileira de Nutrologia. Projeto Diretrizes. Recomendações nutricionais para crianças em terapia nutricional enteral e parenteral. São Paulo; AMBCFM; 2011. p. 1-16 [cited 2019 Dez 10]. Available at: https://diretrizes.amb.org.br/_BibliotecaAntiga/recomendacoes_nutricionais_para_criancas_em_terapia_nutricional_enteral_e_parenteral.pdf;^(65)^ Padovani RM, Amaya-Farfán J, Colugnati FA, Domene SM. Dietary reference intakes: application of tables in nutritional studies. Rev Nutr. 2006;19(6):741-60.^(84)^

ND: not determined.

### Frequent nutritional complications

The immunosuppressive therapy used is aggressive and has high toxicity, causing clinical signs and symptoms such as nausea, vomiting, abdominal pain, altered sense of taste, oropharyngeal mucositis, odynophagia, xerostomia, esophagitis, diarrhea, infections, bleeding and anemia.^(85–89)^ These changes may persist for weeks after transplantation, with negative consequences for the patient, mainly in caloric, protein and nutrient supply, leading to progressive impairment of nutritional status.^(20)^

In addition to increased energy requirements in these patients, aiming to maintain adequate development in this age group, professionals should be mindful of the catabolic effects of the underlying disease, as well as signs and symptoms related to treatments, which can decrease patients’ caloric intake.^(90)^

The daily routine of these patients is deeply affected by these symptoms, warranting the need for effective management by their healthcare provider.^(91)^

### Nausea and vomiting

The diagnosis of nausea is clinical and based on the history reported by patients and their family, due to its subjective nature. It is important to question the patient about their desire to eat, feeling of weakness, mechanical difficulty to swallow, presence of thirst and/or hunger, reaction to certain foods and odors, and symptom-triggering factors. Assessment of the patient’s nutritional status and signs of dehydration can contribute to the diagnosis of chronic and/or intense nausea. A diagnosis of vomiting is also based on clinical history, and it is important to quantify episodes and the volume in each episode.^(92)^

During HSCT, nausea and vomiting may be associated with the conditioning protocol (chemotherapy or radiotherapy), as well as other causes, such as medications (antimicrobials and opioids); systemic infections; metabolic abnormalities, such as hyperkalemia and hyponatremia; adrenocortical insufficiency; increased intracranial pressure; gastric irritations and/or ulcerations; constipation; intestinal obstruction, such as gastroesophageal reflux and gastric stasis; and psychological alterations, such as anxiety and emotional stress. Many patients suffer with nausea and vomiting, which may cause severe discomfort, such as pain, dehydration, hiccups, heartburn and anorexia.^(92)^ Proper care and management of these symptoms are key to providing comfort and quality of life to children or adolescents and their families during all treatment phases.^(92)^

For successful management and control of nausea and vomiting in the pediatric population, a complete evaluation is necessary, considering possible causes, such self-reports by children or adolescents and their caregivers. A detailed past history of nausea and vomiting, the frequency of these symptoms, periods in which they most present, their description and intensity, such as quantity and characteristic of the content expelled, times of the episodes, and associated and/or predisposing factors are extremely important for the efficacy of the treatment of choice.^(93)^

Dietary measures must be adequate to each patient’s individual needs, preferences and eating habits,^(94)^ and, when used together with antiemetic agents, can potentially help reduce their frequency and dose.^(93)^ Some simple measures may help in the control of nausea and vomiting, such as diet fractionation in small meals at shorter intervals, eating meals in quiet and well-ventilated environments, observance of established hours for meals, and the supply of small quantities of carbohydrates and foods preferred by the patient.^(91,92)^ In addition, it is important that the patient’s head be kept elevated for up to 1 to 2 hours after eating; avoid extreme temperatures, favoring foods at room temperature or cold; keep patients away from the kitchen when means are being prepared, since the smell of foods during cooking can aggravate the nausea; and avoid deep-fried, greasy, spicy, savory, acidic, sugary foods, as well as those with strong odors.^(91)^

### Graft *versus* host disease in children

GVHD is one of the main post-procedural complications of BMT. It can be classified as acute (aGVHD) and chronic (cGVHD). Acute GVHD classically occurs in the first 100 days after BMT and affects mostly the skin, liver and gastrointestinal tract (GIT). Chronic GVHD classically starts more than 100 days after BMT and may affect one or more organs. The main sites involved in GVHD are skin, liver, mouth, eyes, musculoskeletal system, lungs and genitals. It can last from several months to years and is similar to an autoimmune disease, characterized by the presence of inflammation and, later, fibrosis of the affected tissues and organs.^(95,96)^

In the GIT, the main clinical manifestations of GVHD, both acute and chronic, are nausea, vomiting, anorexia, diarrhea, abdominal pain and dysphagia. Depending on the intensity of symptoms, patients may develop mild, moderate and severe forms. The condition may also be aggravated by immunosuppressive treatment, which consists, in most cases, of high-dose corticosteroids associated or not with calcineurin inhibitors (cyclosporine and tacrolimus). These medications can potentially exacerbate symptoms, either by direct effect or by facilitating infections. As a result, patients develop severe protein-caloric malnutrition, usually multifactorial, due to reduced food intake, malabsorption and increased energy expenditure. Also, as a result of GVHD and its management, the metabolism of carbohydrates, lipids and proteins is disrupted. In this context, nutritional therapy is extremely important as treatment support, to fight the harmful effects of GVHD and to circumvent the adverse effects of medications.^(76,83,95,96)^

Patients with GVHD have difficulty eating foods for various reasons, depending on the organ involved. Often, they require dietary changes, oral supplements, and nutritional support therapy (NST) to prevent or manage malnutrition.^(95)^ According to Bassim *et al.*,^(97)^ the main indications for the onset of NST are uncontrolled nausea and vomiting, voluminous diarrhea, oral mucosal and esophageal pain, dysphagia, dysgeusia, xerostomia, anorexia, early satiety and weight loss. In particular, aGVHD in the GIT and oral, gastrointestinal and pulmonary cGVHD lead to severe malnutrition and impairment of patients’ functional capacity and quality of life – hence the need for early onset of NST.

According to the Oncology Nutrition Consensus of the National Cancer Institute José de Alencar,^(20)^ some nutritional interventions may be oriented to improving and controlling gastrointestinal symptoms.

Regardless of the type and grade of GVHD, when patients’ food intake is less than 70% of energy requirements for the last 3 days, and symptoms impair adequate nutrition, it is important to intervene with hypercaloric and hyperproteic nutritional supplements.^(20)^

If food intake is less than 60% of energy requirements in the last 3 days, or oral route is contraindicated, enteral nutrition therapy (ENT) may be prescribed.^(20)^ The enteral route, if tolerable and clinically feasible, can be selected because it maintains digestive function and mucous barrier integrity, preventing bacterial translocation in the digestive tract.^(83)^

According to a systematic review by Baumgartner et al.,^(76)^ several studies have compared ENT with parenteral nutrition therapy (PNT), showing superior results for the enteral route, and moderate to high tolerance of the tube, whereas PNT is recommended only in cases of gastrointestinal insufficiency. ENT is contraindicated in the presence of hemodynamic instability and/or worsening of abdominal pain, abdominal distension, mucositis, diarrhea, incoercible vomiting, paralytic ileus and intestinal bleeding.^(20)^

However, particularly among patients with GVHD in the GIT, most often there is no tolerance to oral or enteral nutrition. In the presence of severe diarrhea (>1L a day), oral fasting for days to weeks is essential to alleviate gastrointestinal complaints. Nutritional support consists of PNT.^(82)^ Studies have shown that patients with grade III to IV GVHD receive more PNT than patients with grade zero to II GVHD, and are not free from clinical complications related to the progression of days on PNT. ^(74)^

As the volume of diarrhea decreases during intestinal GVHD (<500mL per day), oral feeding can be resumed, but certain foods may be better tolerated than others.^(83)^ Many centers use nutritional therapy based on a phased diet, which, according to symptoms and patient tolerance, may progress or regress. According to the general guidelines of the Seattle Cancer Care Alliance,^(98)^ progression of specific diets should include limited contents of fat, fibers, lactose, acid foods and gastric irritants, to be gradually reintroduced based on patient tolerance, as shown in table 15.^(99)^

**Table 15:** Progression of nutritional therapy for graft *versus* host disease in the gastrointestinal tract based on the Seattle Cancer Care Alliance protocol

Phase	Symptoms	Nutritional therapy
First phase: bowel rest	Large-volume aqueous diarrhea; intestinal colic; serum albumin depletion; decreased intestinal transit; intestinal obstruction; nausea and vomiting	Parenteral nutrition only
Second phase: introduction of oral feeding	Diarrhea volume less than 500mL/day; decreased intestinal colic; improved intestinal transit time; decreased nausea and vomiting	Parenteral nutrition + isosmotic, low-residue, lactose-free, low-acid, low-fat oral liquid diet
Third phase: introduction of solid foods	Absent or decreased colic and more consistent stools	Oral diet: introduction of low-residue, lactose-free, low-fat, low-acid, non-irritating solid foods
Fourth phase: diet expansion	Absent or decreased colic and more consistent stools	Low-fiber, lactose-free, low-acid, non-irritating, low-fat foods, according to patient tolerance
Fifth phase: introduction of regular diet	No colic and normal stool consistency	Gradual introduction, according to patient tolerance, of acid, gastric irritant, fiber-containing, lactose-containing and high-fat foods

Source: Flowers ME, McDonald G, Carpenter P, Boeckh M, Sanders J, Deeg J, et al. Long-term follow-up after hematopoietic stem cell transplant general guidelines for referring physicians. Seattle (WA): Fred Hutchinson Cancer Research Center/ Seattle Cancer Care Alliance; 2019. p.88 [cited 2019 Nov 26]. Available from: https://www.fredhutch.org/content/dam/public/Treatment-Suport/Long-Term-Follow-Up/physician.pdf;^(98)^ Viani K, Leao AC, Mantovani LF. Transplante de células tronco-hematopoieticas. In: Viani K, Oliveira V, Nabarrete J, Silva AP, Feferbaum R. Nutrição e câncer infanto-juvenil. São Paulo: Manole; 2017. p. 188-201.^(99)^

Immunosuppression with corticosteroids is the foundation of first-line therapy in aGVHD and cGVHD.^(100)^ The use of calcineurin inhibitors is also common for prevention and management of these two types of GVHD.^(83)^

During treatment with high-dose glucocorticoids and/or calcineurin inhibitors, proper patient guidance is important. Frequent and smaller meals are recommended, as well as soluble and insoluble fiber-rich foods, high-protein diet, with low-simple, high-glycemic carbs, low sodium, adequate water intake, and adequate intake of foods that are sources of vitamin D, calcium, magnesium and zinc and, if necessary, supplementation of these elements.^(99,101)^

Monitoring vitamin and mineral levels is of paramount importance in the GIT GVHD, due to the presence of diarrhea and poor nutrient absorption in these patients.^(102)^ Vitamin B_12_ levels should be observed more carefully due to intrinsic factor insufficiency in patients with gastric GVHD. Similarly, zinc levels are relevant for their role in maintaining intestinal mucosal integrity and taste acuity, and improving dysgeusia.^(83)^

Supplementation of other nutrients, such as omega 3, glutamine, arginine and nucleotides, seems beneficial in patients with GVHD, however studies are scarce and with small populations, and more scientific evidence is needed for safe prescription.^(83)^

aGVHD and cGVHD, complications that evolve with severe nutritional changes, make patients susceptible to malnutrition. If not managed properly, they reduce patient survival and quality of life, and follow-up is required for those affected, as well as nutritional intervention.

### Mucositis

Oral mucositis is an inflammatory condition^(103–105)^ and one of the main complications associated with HSCT, affecting 60% to 80% of cases undergoing myeloablative conditioning regimens.^(106,107)^

Although it can occur throughout the digestive tract, oral mucositis is considered one of the most uncomfortable and painful experiences during treatment,^(103,108)^ and is often the main cause of discomfort in early stages of treatment. In this phase, it is common to see reduced food intake, nutritional impairment, weight loss, infectious complications, need for parenteral nutrition, use of opioids and, sometimes, prolonged hospital stay.^(106,109)^

The most commonly used criteria for mucositis classification are those defined by the WHO and the National Cancer Institute (NCI),^(110)^ which take into account signs and symptoms such as pain, erythema, ulcerations, function and feeding capacity (Table 16).

**Table 16:** Grading scale classification of oral mucositis

Grading scale	Grade					
	0	1	2	3	4	5
NCI CTCAE	Absence	Asymptomatic or mild symptoms; no indication for interventions	Moderate pain; oral intake tolerable	Severe pain; oral intake not possible	Life-threatening lesions; need for urgent interventions	Death
WHO	Absence	Pain and erythema	Ulcerations allowing intake of solid foods	Ulcerations allowing intake of liquids only	Oral intake not possible	

Source: Chaudhry HM, Bruce AJ, Wolf RC, Litzow MR, Hogan WJ, Patnaik MS, et al. The incidence and severity of oral mucositis among allogeneic hematopoietic stem cell transplantation patients: a systematic review. Biol Blood Marrow Transplant. 2016;22(4):605-16. Review.^(109)^

NCI: *National Cancer Institute*; CTCAE: *Common Terminology Criteria for Adverse Events*; WHO: World Health Organization.

Mucositis is clinically characterized by pain, edema, erythema, ulcerations and formation of pseudomembranes. These changes are associated with limitation of oral cavity, speech and swallowing functions, impacting the quality of life of patients.^(103–105,111)^

The etiology of pain in oral mucositis is of both nociceptive and neuropathic origin. Mechanical nociception results in oral dysfunction of patients with oral mucositis. Neuropathic pain is caused by neuronal sensitization resulting from the action of chemotherapeutic agents and inflammatory mediators (glutamate, neuropeptides and proinflammatory cytokines, such as interleukin – IL – 1, IL-6 and tumor necrosis factor alpha – TNF-α), which aggravates neuronal sensitization.^(103)^

The clinical course of mucositis is usually predictable, with the first signs appearing between 3 and 4 days after onset of conditioning and ulcerations appearing soon afterwards, and more intensely between days 7 and 14, usually with spontaneous resolution in the following week or concomitant to grafting.^(106,111)^

The incidence and severity of mucositis cases are associated with treatment-related factors,^(112)^ such as myeloablative conditioning regimens,^(113)^ particularly those containing TBI, high-dose melphalan and busulfan, and use of methotrexate for GVHD prophylaxis.^(105,109,114)^

**Figure 1:**
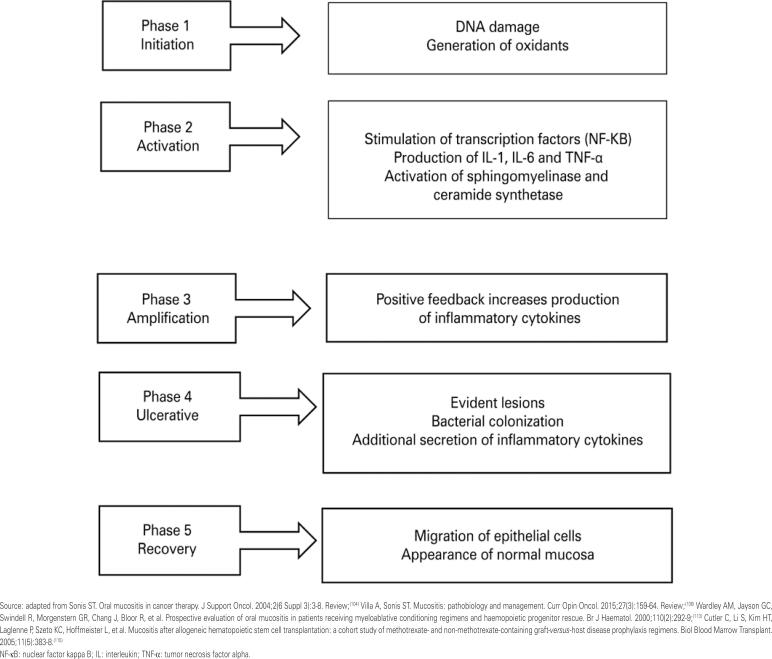
Development phases of mucositis

The pathogenesis of mucositis is multifactorial, and current models include different biological events during the course of mucositis, with a chronological process of five different, independent phases, which involve, in chronological order, cell initiation/death; generation of oxidative agents/activation of inflammatory cytokines; amplification and signaling; ulceration and recovery/cure, as shown in figure 1.^(104,106,113,115)^

Management of mucositis involves management of symptoms and prevention of complications, including pain control, nutritional support, prophylaxis and treatment of secondary infections, as shown in table 17.^(105,116)^

**Table 17:** General care for mucositis prophylaxis

Area of care	Actions
Oral hygiene and basic oral care recommendations	Brush teeth 2 to 3 times a day with soft or extra-soft bristle brushes and fluorinated toothpaste (in case of burning, use kids’ toothpaste minimally flavored)
	Use dental floss after each meal
	Rinse mouth with gentle solutions 4 to 6 times per day (sterile water or sodium bicarbonate) and consider using mouth moisturizer
	Avoid mouth rinse solutions with alcohol or peroxide and toothpastes with sodium lauryl sulphate
Oral evaluation by expert	To be conducted before the onset of chemotherapy or radiotherapy, with periodontal and preoperative evaluation, and management as appropriate
	Potentially traumatic factors (dental or prosthetic) must be removed, and regular follow-up maintained during and after treatment
Therapeutic proposals	Growth factors and cytokines
	Coating agents and analgesics
	Laser therapy
	Cryotherapy

Source: Bensinger W, Schubert M, Ang KK, Brizel D, Brown E, Eilers JG, et al. NCCN task force report. prevention and management of mucositis in cancer care. J Natl Comp Canc Netw. 2008;6 (Suppl 1):S1-21; quiz S22-4. Review;^(111)^ Cutler C, Li S, Kim HT, Laglenne P, Szeto KC, Hoffmeister L, et al. Mucositis after allogeneic hematopoietic stem cell transplantation: a cohort study of methotrexate- and non-methotrexate-containing graft-*versus*-host disease prophylaxis regimens. Biol Blood Marrow Transplant. 2005;11(5):383-8;^(115)^ Lalla RV, Sonis ST, Peterson DE. Management of oral mucositis in patients who have cancer. Dent Clin North Am. 2008;52(1):61-77, viii. Review;^(116)^ Vigarios E, Epstein JB, Sibaud V. Oral mucosal changes induced by anticancer targeted therapies and immune checkpoint inhibitors. Support Care Cancer. 2017;25(5):1713-39. Review;^(117)^ Lopes NN, Plapler H, Lalla RV, Chavantes MC, Yoshimura EM, da Silva MA, et al. Effects of low-level laser therapy on collagen expression and neutrophil infiltrate in 5-fluorouracil-induced oral mucositis in hamsters. Lasers Surg Med. 2010;42(6):546-52;^(118)^ Oberoi S, Zamperlini-Netto G, Beyene J, Treister NS, Sung L. Effect of prophylactic low level laser therapy on oral mucositis: a systematic review and meta-analysis. PLoS One. 2014;9(9):e107418. Review;^(119)^ Saadeh CE. Chemotherapy- and radiotherapy-induced oral mucositis: review of preventive strategies and treatment. Pharmacotherapy. 2005;25(4):540-54. Review.^(120)^

After oral mucositis is present, management should be palliative only. Glutamine (L-glutamine or L-alanyl-L-glutamine) is used at high concentrations by rapidly dividing cells, so its use in prevention and management of oral mucositis has been studied, although its relevance has not yet been proven.^(99)^

Cryotherapy is widely used in the prevention of mucositis caused by chemotherapy agent with a short half-life, such as bolus 5-fluorouracil or high-dose melphalan in HSCT conditioning regimes. It is usually performed with ice water or ice chips. In pediatrics, it is possible to encourage this practice by offering ice cream and popsicles or using frozen pacifiers or bottle nipples by dipping them in drinking water and then chilling them in a freezer, or even freeze breast milk or formula in the form of popsicles and offer to the infant 10 minutes before the start until the end of the drug infusion.^(99)^

After oral mucositis is present, oral nutritional therapy consists of dietary adaptations, according to table 18, and/or parenteral nutrition. If the patient already has a nasoenteral tube, enteral diet can be used. Otherwise, passing a nasoenteral tube not indicated in patients with established severe oral mucositis.

**Table 18:** Dietary changes in oral diet for patients with oral mucositis

Dietary changes for oral mucositis
Food and liquids at room temperature, warm or cold
Preparations in pasty and/or soft consistency
Avoid acid foods and alcoholic and/or carbonated beverages
Avoid strong and spicy seasonings and excess salt
Choose foods with sauces

### Anorexia and cachexia

Anorexia (loss of appetite) is a common concomitant symptom in people with cancer. Cancer anorexia has many causes, but the primary cause is often an increase in proinflammatory cytokines or lactate. These two factors, therefore, modulate the neurotransmitter cascades of the central nervous system.^(121)^

Cancer cachexia is a multifactorial syndrome covering a spectrum from initial weight loss (pre-cachexia) to a state of severe disability incompatible with life. The main defining characteristics of cachexia in humans are weight loss, reduced food intake and systemic inflammation. Nutritional support in cachexia can stabilize and improve nutritional status, function and quality of life.^(121)^

There is a large prospective study in which post-BMT patients were followed up for up to 6 years and had a lean mass index lower than that of healthy controls. Those with cGVHD and on corticosteroids were the most affected.^(122)^

Factors such as persistent vomiting and nausea, constipation, diarrhea (induced by chemotherapy, infection and GVHD), mucositis (induced by chemotherapy, infection and GVHD), altered or lost sense of taste, as well altered sense of smell and metabolic disorders, may cause patients undergoing HSCT to reduce their food intake and lose weight.^(123)^

More than any other, anorexia is seen as the main symptom of onset of clinical and metabolic signs that precede cachexia, potentially causing significant and involuntary weight loss.^(124)^

In patients with CTT, socioeconomic, medical and physical factors may lead to anorexia-cachexia syndrome, even after hospital discharge.^(121)^ therefore, signs and symptoms, such as anorexia, weight loss, changes in biochemical tests and changes in body composition, should be frequently followed up, so that nutritional intervention occurs as early as possible, avoiding interference in the performance status and quality of life of the patient, As well as with the increased risk of complications, such as GVHD.^(125–127)^

Therefore, it is necessary to think about all the symptoms present and how to manage them. The work of the nutrition team is to show the patient and their family the importance of eating, however, with no pressure and without ever forcing the patient to eat. The goal is to increase the amount of food ingested, in addition to normalizing appetite and food acceptance, to meet or get as close as possible to nutritional requirements.^(124)^

Some nutritional recommendations may be made before these symptoms appear, such as^(124)^ increasing meal fractionation; reducing meal volume, eating smaller portions (*e.g*. finger food); replacing meals with complete snacks; modifying food consistency, if necessary; avoiding greasy or rich sauce preparations; improving the presentation of meals, using different utensils, such as casseroles and colored plates; eating meals in pleasant surroundings; allowing to choose meals according to acceptance, at different treatment stages, as a strategy to increase intake; avoiding excessive pressure to eat; avoiding drinking too much liquids, especially during meals; increasing caloric density of preparations, using nutritional supplements, if necessary; and using enteral and/or parenteral diet, if necessary, according to the patient’s nutritional and clinical condition.

### Drug-related nephrotoxicity

Renal complications are very frequent and contribute to procedure-related morbidity and mortality. The causes may be conditioning chemotherapy, TBI, nephrotoxic agents (*e.g*., calcineurin inhibitors), infections, liver SOS (formerly known as veno-occlusive disease - VOD), transplant-associated thrombotic microangiopathy, and GVHD.^(128)^

We know that the incidence of acute kidney injury (AKI) is less frequent in autologous transplantation and more prevalent mainly in myeloablative allogeneic transplantation. The onset (within the first 30 days after transplantation) and severity of kidney function worsening are associated with a gradual increase in the risk of death decrease in overall survival. In patients requiring dialysis, mortality may vary between 55% and 100% of the cases.^(128)^ The incidence of chronic kidney disease (CKD) varies greatly, from 7% to 48% of cases, and it can occur from 6 months to 10 years after transplantation.^(129)^

In the initial evaluation of patients undergoing HSCT who present with signs of kidney impairment, one should attempt to establish the cause of this deterioration. It is important to obtain a complete urine examination, the urinary albumin-urine-creatinine ratio, a complete blood count with blood smear evaluation, and serum lactate dehydrogenase, haptoglobin and calcineurin inhibitors levels. Viral screening, particularly BK and adenovirus, should also be carried out. In individualized cases, kidney biopsy can be considered, to potentially identify GVHD, endothelial lesion and chronic inflammation.^(128,129)^

Nephrotoxic agents frequently used in transplantation can be divided into three major classes: chemotherapeutic agents (*e.g.*, cyclophosphamide, carboplatin and fludarabine); antimicrobials, antivirals and antifungal agents (*e.g.*, vancomycin, acyclovir and amphotericin) and immunosuppressants (*e.g.* calcineurin inhibitors such as cyclosporin and tacrolimus).^(128)^

Up to 70% of children and adults undergoing HSCT have hypertension for the first 2 years after the procedure. Systemic arterial hypertension in children and adolescents is defined as systolic or diastolic BP above the 95th percentile for gender, age and height, measured in at least three different occasions. Predisposing factors include treatment with cyclosporine, acute kidney injury, TBI, obesity and diabetes. Hypertension has been associated with a higher probability of CKD. Its management must include dietary and lifestyle modifications, in addition to drug therapy, when necessary.^(129,130)^

When AKI is established, not only water, electrolytic and acid-basic metabolism is affected, but there is also interference in the metabolism of all macronutrients, leading to pro-inflammatory, pro-oxidative and hypercatabolic situations. In addition to consequences related to AKI itself, there are also contributions of the underlying disease, HSCT and its complications, which may worsen the patient’s nutritional depletion, leading to the so-called protein-energy wasting syndrome (PEW).^(131)^

The therapeutic approaches targeted at delaying progression of kidney injury include reduction of protein intake to control the glomerular filtration rate (GFR). Progressive loss of renal function leads to CKD, classified in stages 1 to 5 based on GFR changes, which determine when dialysis should be initiated.^(132)^

Recommended protein amounts follow the recommendations of the National Kidney Foundation and Kidney Disease Outcomes Quality Initiative (NFK KDOQUI)™, as shown in table 19.^(132)^

When dialysis is needed, be it hemodialysis or peritoneal dialysis, protein supply should be maintained at 100% of DRI for ideal weight plus the amount corresponding to proteins and amino acids lost in the process (Table 20).^(132)^

**Table 19:** Protein recommendation in stage 3 to 5 chronic kidney disease

Age	DRI (g/kg/day)	Stage 3 CKD recommendation (g/kg/day) 100%-140% DRI	Stage 4 to 5 CKD recommendation (g/kg/day) 100%-120% DRI
0-6 months	1.5	1.5-2.1	1.5-1.8
7-12 months	1.2	1.2-1.7	1.2-1.5
1-3 years	1.05	1.05-1.5	1.05-1.25
4-13 years	0.95	0.95-1.35	0.95-1.15
14-18 years	0.85	0.85-1.2	0.85-1.05

Source: Takakura CY, Murakami DK. Nefropatias. In: da Silva AP, Nascimento AG, Zamberlan P. Manual de dietas e condutas nutricionais em pediatria. São Paulo: Atheneu; 2014. p. 383-92.^(132)^

DRI: dietary reference intakes; CKD: chronic kidney disease.

**Table 20:** Protein recommendations in dialysis

Age	Hemodialysis (g/kg/day) DRI + 0.1g/kg/day	Peritoneal dialysis (g/kg/day) DRI + 0.15 to 0.3g/kg/day
0-6 months	1.6	1.8
7-12 months	1.3	1.5
1- 3 years	1.15	1.3
4- 13 years	1.05	1.1
14- 18 years	0.95	1.0

Source: Takakura CY, Murakami DK. Nefropatias. In: da Silva AP, Nascimento AG, Zamberlan P. Manual de dietas e condutas nutricionais em pediatria. São Paulo: Atheneu; 2014. p. 383-92.^(132)^

DRI: Dietary reference intakes.

Carbohydrates, lipids, sodium, potassium, calcium, phosphorus and liquids vary according to the level of kidney function impairment and comply with the recommendations in tables 21 and 22.^(132)^

**Table 21:** Recommendations for macronutrients

Macronutrient	1-3 years % of total energy value	4-18 years % of total energy value
Carbohydrate	45-65	45-65
Lipid	30-40	25-35
Protein	5-20	10-30

Source: Dalle JH, Giralt SA. Hepatic veno-occlusive disease after hematopoietic stem cell transplantation: risk factors and stratification, prophylaxis, and treatment. Biol Blood Marrow Transplant. 2016;22(3):400-9. Review.^(133)^

**Table 22:** Recommendations for micronutrients and liquids

Age	Sodium (mg/day)	Potassium (mg/day)	Calcium (mg/day)	Phosphorus (mg/day)	Liquids (mL/day)	Vitamins and other minerals
0-6 months	120	40-120	100%-200% DRI (daily intake + medication)	Hyperphosphatemia 80% of DRI	Insensible loss according to age + residual kidney function + other losses	100% DRI if proven deficiency, specific supplementation
7-12 months	370					
1-3 years	1,000					
4-13 years	1,200 - 1,500					
14-18 years	1,500					

Source: Takakura CY, Murakami DK. Nefropatias. In: da Silva AP, Nascimento AG, Zamberlan P. Manual de dietas e condutas nutricionais em pediatria. São Paulo: Atheneu; 2014. p. 383-92.^(132)^

DRI: dietary reference intakes.

### Sinusoidal obstruction syndrome

SOS, formerly called VOD, can be a serious complication after HSCT. It was initially described in patients who consumed tea containing pyrrolizidine alkaloids. Although it may occur in many other settings, such as after liver irradiation, exposure to hepatotoxic chemotherapy agents, use of azathioprine, or, more recently, gemtuzumab ozogamycin, SOS is most commonly seen in the context of high-dose chemotherapy with HSCT, and was first described in 1979.^(133)^

It usually develops within the first 30 days after HSCT, although it may occur later. Historically, its incidence varies from approximately 5% to 60% in adults, and this variation is not only related to the intensity of the conditioning regimen, the type of transplantation and the presence of risk factors, but also to the clinical criteria used in diagnosis. In general, the frequency and severity of SOS have decreased in recent years as a result of changes in preparative regimens. Severity varies greatly, ranging from mild forms, which resolve within a few weeks, to a severe syndrome, defined by the presence of multiple organ failure and associated with high mortality.^(134)^

The incidence of SOS in pediatric patients undergoing HSCT ranges from 11% to 31%, with an associated mortality rate of up to 50%.^(135)^

SOS occurs due to damage caused by the conditioning regimen to liver sinusoidal endothelial cells and hepatocytes, with fibrinogen deposition, factor VIII, and erythrocyte congestion, resulting in sinusoidal enlargement and occlusion, which can progress to abnormal liver architecture and centrilobular necrosis. In late stages of the disease, there is fibrosis and occlusion of terminal venules, leading to liver failure and possibly death. In parallel to physical damage, there is a procoagulant state with increased levels of plasminogen activator inhibitor 1 and low levels of antithrombin, protein C and factor VII.^(136)^

Understanding the risk factors associated with SOS development is important for early treatment or prophylaxis. Overall, risk factors can be divided into two categories: pre-transplant patient characteristics and transplant-related factors. The main transplant-related risk factors related for SOS are^(134)^ allo HSCT, unrelated donor, HLA-incompatible donor, myeloablative conditioning regimen, busulfan- or TBI-based conditioning regimen, non-T-cell-depleted grafts, and second HSCT. Patient-related factors are: older patients (in the adult population); women on norethisterone; Karnofsky score under 90%; genetic polymorphism (GSTM1, GSMTT1 and heparanase); advanced diseases (after second remission or recurrence); metabolic syndromes; antithrombin III deficiency, and plasminogen activator factor; activated protein C resistance and thalassemia. Liver-related factors are transaminases > 2.5 times the upper limit of normality; serum bilirubin > 1.5 time the upper limit of normality; cirrhosis; liver fibrosis; active viral hepatitis; liver irradiation; previous use of gemtuzumab ozogamycin; use of hepatotoxic drugs; and iron overload. Specific factors of the pediatric population include: hemophagocytic lymphohistiocytosis, adrenoleukodystrophy and osteopetrosis; autologous HSCT with high-dose chemotherapy in neuroblastoma; young age (under 1 to 2 years); low weight and juvenile myelomonocytic leukemia.

SOS is clinically characterized by fluid retention and ascites, jaundice, weight gain and tender hepatomegaly, in the absence of other identifiable causes of liver disease. There are two classic clinical criteria for a diagnosis of SOS. One of them is the Baltimore classification, according to which, in the first 21 days after HSCT, patients must present with bilirubin ≥2mg/dL and two or more of the following findings: tender hepatomegaly, ascites and weight gain (≥5% of baseline weight). There is also the Seattle criteria, establishing, within the first 20 days after HSCT, two or more of the following findings: bilirubin ≥2mg/dL, hepatomegaly or pain in the upper right quadrant of the abdomen and weight gain (≥2% of the baseline weight).

The triad formed by weight gain, tender hepatomegaly, and high bilirubin varies in less severe cases, and can be incomplete or delayed in pediatric patients compared with adults. These clinical criteria can be complemented by noninvasive examinations, such as ultrasound, to identify ascites, hepatomegaly, thickening of the gallbladder wall, and attenuation or reversal of hepatic venous flow. The use of invasive tests (*e.g.*, percutaneous or transjugular liver biopsy) should be weighed against the risk of bleeding associated with these procedures. In addition to hyperbilirubinemia, other SOS-related laboratory findings include increased transaminases, prolonged prothrombin time, and signs of decreased synthetic function (*e.g.*, low albumin).^(133)^

Although jaundice is usually present in adults, it may be absent in SOS developing late after HSCT, and is often absent in children. It should not be a prerequisite for SOS diagnosis.^(137,138)^

SOS has been retrospectively classified as mild, moderate, or severe, based on disease severity, including the degree of liver dysfunction and the need for therapy. However, these criteria are loosely defined and cannot be used to predict risk or guide treatment.^(133)^

Considering the high mortality rate associated with severe SOS, strict daily monitoring to for early detection of signs and symptoms of the syndrome must be present from the onset of conditioning. Weight gain, fluid retention, overt edema and ascites, hepatomegaly and jaundice must be monitored on a daily basis. Patients with one of these risk factors require greater attention.

Other findings may be present, such as symptoms related to fluid retention (pleural effusion, pulmonary infiltrate, and hypoxia). Early onset of transfusion-refractory thrombocytopenia, not explained by concomitant conditions, such as sepsis, may be an initial sign of SOS, reflecting the endothelial pathophysiology of the syndrome. The presence of kidney or pulmonary dysfunction (or, less frequently, central nervous system dysfunction) defines multiple organ failure and severe SOS.^(134)^

The adoption of preventive measures to potentially reduce the incidence and/or severity of SOS is imperative, especially since there are no 100% effective therapeutic measures for this disease. Preventive measures combine two approaches: reversal of risk factors and pharmacological prevention. The use of heparin is still quite controversial. Data on the usefulness of ursodesoxicolic acid are not conclusive: some randomized trials suggest that it reduces the incidence of SOS, whereas others did not find said advantage. However, patients who received this prophylaxis had lower liver toxicity, less a GVHD and better survival, which strongly suggests a beneficial effect. In addition, the use of ursodesoxicolic acid has been associated with decreased non-recurrence-related mortality.^(134)^

Specifically considering the pediatric population, several strategies for SOS prevention have been investigated, including lipoprostaglandin E1, prophylaxis with danaparoid, and a prophylactic regimen combining heparin, glutamine and ursodiol. A retrospective Korean study with 374 children undergoing HSCT showed that prophylaxis with lipoprostaglandin E1 may have a protective effect against SOS.^(136)^ A retrospective review of 188 children who received a prophylactic regimen combining intravenous heparin, oral glutamine and ursodiol prior to HSCT demonstrated low incidence of SOS using this approach (one case in 188 patients).^(139)^ Muscaritoli *et al.*,^(73)^ reported that glutamine administered during HSCT potentially has a protective effect in the liver against SOS. However, prospective studies are needed to better assess the impact of these strategies. Finally, a prospective, randomized, phase III study recently demonstrated a lower incidence of SOS in pediatric patients undergoing HSCT who received defibrotide prophylaxis.^(140)^ Some other studies investigating the use of defibrotide prophylaxis in children also reported decreased incidence of SOS, as well as a favorable toxicity profile.^(141)^

The first step in managing SOS is symptomatic treatment. Considering that this is a potentially fatal disease, therapy should be initiated as soon as possible. Management of salt and water balance and careful use of diuretics should be introduced at the first suspicion, when SOS is still only probable. The purpose of support care is to minimize extracellular fluid overload, without worsening kidney function. Symptomatic measures can be used to reduce the discomfort caused by massive ascites or pleural effusions. Particularly in infants, when massive ascites affects breathing due to pulmonary restriction, early paracentesis can be extremely useful to prevent mechanical ventilation-associated complications. When fluid buildup and kidney failure cannot be controlled, hemodialysis/hemofiltration is required. The treatment of the severe SOS requires transfer to the intensive care unit.^(133,134)^

Several clinical studies have shown the efficacy of defibrotide for managing SOS. In Europe, it is indicated for management of severe SOS in adults, adolescents, and children over 1 month of age. In the United States, defibrotide was recently approved by the US Food and Drug Administration for the treatment of adult and pediatric patients with SOS and kidney or pulmonary dysfunction after HSCT.^(141,142)^

When SOS is present, nutritional status changes occur, characterized by reduced anabolism. The main consequence is weight gain from kidney retention of sodium and water. In this case, nutritional therapy aims to favor reversal of intravascular fluids and electrolyte loss.^(143,144)^ Bear in mind that in the period of highest incidence for SOS, weight gain must be cautiously monitored, as one of the initial signs for SOS diagnosis.

There is no specific nutritional recommendation for SOS. Some authors cited restriction of dietary branched-chain amino acids to prevent progression to hepatic encephalopathy.^(73,145)^ However, no studies were found testing this restriction in these patients and supporting its indication.

In 2009, the European Society for Clinical Nutrition and metabolism (ESPEN)^(146)^ recommended restriction of dietary branched-chain amino acids only in cases of severe encephalopathy, grades 3 and 4. In the latest publication of the American Society, this measure was recommended only in cases of refractory encephalopathy in critical patients.^(147)^

Thus, it is recommended that nutritional therapy be performed according to general HSCT treatment recommendations.

### Endocrine and metabolic complications

Endocrine and metabolic complications may occur in children, even in those undergoing reduced intensity conditioning and no radiotherapy. This is because the endocrine system is extremely susceptible to damage by the conditioning regimen. The most common endocrine complications are hypothyroidism, gonadal failure, reduced bone mineral density and short stature due to growth hormone deficiency or hypopituitarism. This is an issue that has been increasingly explored in the scientific literature and is an important concern, particularly in pre-pubertal children and adolescents, who are still growing and developing.^(148)^

Growth deficits in children undergoing HSCT occur due to a myriad of factors.^(149)^ A study carried out with 181 patients subjected to HSCT during childhood, who had reached their final height, showed that 80% of them had a height within normal parameters. Greater impairment was observed in boys than in girls, in children transplanted at a younger age or who received TBI.^(10)^ Total body irradiation also leads to thyroid function abnormalities. Hypothyroidism can be an immediate and also late post-HSCT complication, identified as late as years after the procedure. Approximately 15% of patients develop primary hypothyroidism, which should be properly treated to prevent greater clinical repercussions.^(10)^

Reductions in bone mineral density are common both a few months after HSCT and several years after (more than 10), with a prevalence of osteopenia and osteoporosis in this population between 24% and 57%. Presence of cGVHD, use of corticosteroids and low serum vitamin D levels are risk factors for reduced bone mineral density in post-HSCT children.^(17)^ Several studies have demonstrated the high prevalence of vitamin D deficiency, alerting to the association between low levels of this vitamin and presence or progression of GVHD, since vitamin D may be involved in immune response control, inhibiting T-cell proliferation and cytokine production; vitamin D testing and supplementation are recommended before and after HSCT,^(16,150)^ based on the DRI. It is important to evaluate dietary calcium intake, particularly in patients with GVHD or receiving corticosteroids, where the recommended intake of this mineral is higher. Supplementation should be prescribed to complement food intake when the latter does not meet the Recommended Dietary Allowance (RDA) (Table 23). Calcium intake beyond these recommendations can be harmful due to interference in absorption of other nutrients.

**Table 23:** Recommendations for daily calcium and vitamin D

Age (years)	Recommended daily intake		Recommended daily intake during corticosteroid use	
	Vitamin D (IU)	Calcium (mg)	Vitamin D (IU)	Calcium (mg)
1-3	600	700	600-1,000	800
4-8	600	1,000	600-1,000	1,200
9-13	600	1,300	600-1,000	1,500
14-18	600	1,300	600-1,000	1,500

Source: Campos DJ, Boguszewski CL, Funke VA, Bonfim CM, Kulak CA, Pasquini R, et al. Bone mineral density, vitamin D, and nutritional status of children submitted to hematopoietic stem cell transplantation. Nutrition. 2014;30(6):654-9;^(16)^ Shalitin S, Phillip M, Stein J, Goshen Y, Carmi D, Yaniv I. Endocrine dysfunction and parameters of the metabolic syndrome after bone marrow transplantation during childhood and adolescence. Bone Marrow Transplant. 2006;37(12):1109-17.^(151)^

Type 2 diabetes is another complication that can occur after HSCT, but at a lower prevalence than other endocrine complications, with risk factors including family history of diabetes, non-Caucasian race and leukemia diagnosis.^(149,151)^ The use of corticosteroids can also induce diabetes during use of these drugs.^(151)^ Children undergoing HSCT are also at risk for development of insulin resistance and abdominal fat accumulation.^(152)^ Metabolic syndrome, characterized by hypertension, dyslipidemia, obesity and impaired glucose metabolism, leads to increased risk of early cardiovascular disease and type 2 diabetes, and occurs in 7% to 32% of children undergoing HSCT.^(58,153,154)^

For monitoring and prevention of endocrine and metabolic changes, the international consensus on late effects after HSCT in pediatric patients recommends^(131)^ to assess: thyroid function annually; growth rate annually, adding determination of bone age in those with non-ideal growth; bone mineral density by body densitometry prior to HSCT, repeated after 1 and 5 years, except for patients with reduced bone mineral density, who should be evaluated annually; and lipid profile and fasting blood glucose at least every 5 years, and annually when abnormal. Serum levels of calcium, magnesium, and vitamin D (25OHD) after HSCT should also be monitored, especially in patients with abnormal bone mineral density. Patients should be instructed to follow a healthy diet, with adequate calcium and vitamin D consumption. They should also be alerted to the negative effects of smoking, alcohol, and caffeine consumption, and encouraged to take on physical activities.

In the post-HSCT setting, patients are recommended to follow a healthy diet, aiming at prevention not only of endocrine and metabolic complications, but also of other chronic diseases and cancers. The entire family of the transplanted patient should be encouraged to follow an adequate eating pattern, to help optimize patients’ understanding and adherence, promoting health education and disease prevention throughout the family. Two of the main points to be improved are promoting adequate consumption of fruits, vegetables, and legumes, and restricting consumption of processed foods. The nutritionist should base these guidelines on materials from the Ministry of Health.^(155,156)^

### Dysgeusia

Dysgeusia is defined as persistent distortion or decrease in the sense of taste.^(157,158)^ Several mechanisms may be involved in the cause of taste and smell disorders frequently observed in patients with oncohematological diseases, such as abnormal neuronal activity (abnormal sensitization of some branches of the facial nerve), imbalance in oxidant and antioxidant levels (due to peroxidation of epithelial cells in the oral cavity), and direct destruction of olfactory and gustatory receptor cells.^(158)^

Chemotherapy protocols use drugs that interfere with mitotic activity, with the aim of destroying highly proliferative cells (cancer cells). Since taste bud cells are highly proliferative, the renewal of these cells is also interrupted during the treatment period.^(159)^ Damage may also occur to the epithelium of the oral mucosa, caused by direct action of radiation on taste buds in case of patients undergoing radiotherapy in the head and neck.^(160,161)^ It is estimated that these changes range from 40 to 75%.^(157,158,162–167)^

Dysgeusia is often associated with olfactory alterations, since the two senses are closely involved in generating the sensation of taste.^(168)^ Several studies have shown association between dysgeusia and reduced oral intake, increased parenteral nutrition time, significant weight loss and reduced functional capacity in patients during treatment,^(169–171)^ with a negative impact on patient quality of life, and being one of the main causes of refusal to eat in children.^(161,172,173)^

In the context of pediatric HSCT, some types of conditioning, such as those using high-dose melphalan, and the presence of oral mucositis are independent risk factors for dysgeusia, whereas the use of cryotherapy seems to be an independent protective factor.^(172)^ Oral cryotherapy leads to vasoconstriction and decreased blood flow in the oral cavity, reducing exposure of the oral mucosa to chemotherapy and reducing the incidence of oral mucositis.^(174–176)^ The procedure consists of sucking small ice cubes 30 minutes before, during the 60 minutes of chemotherapy infusion (*e.g.* melphalan), and 30 minutes after infusion.^(177)^

Several drug treatments have been described, but still without proven efficacy:^(157,178,179)^

- Zinc supplementation (alkaline phosphatase cofactor): has shown efficacy in improving gustatory function and overall quality of life scores in patients with idiopathic dysgeusia.^(157,168)^ Studies carried out later in patients with head and neck cancer undergoing radiotherapy and/or chemotherapy showed slight improvement in dysgeusia symptoms in patients undergoing chemotherapy and/or radiotherapy.^(157,179,180)^
- Amifostine (cytoprotective agent): despite the proven effect in decreasing the incidence and severity of chemotherapy and/or radiotherapy-induced toxicity due to its antioxidant effect, studies have not demonstrated any benefits in prevention and/or management of dysgeusia.^(158,181,182)^
- Individualized nutritional advice: through direct verbal guidance, leaflets and/or audio lessons, it seems to have a beneficial effect in decreasing symptoms, with a better effect on long-term than early-onset dysgeusia. The main guidelines are described in table 24.^(157,183,184)^

**Table 24:** Summary of nutritional guidance to decrease symptoms of dysgeusia

Nutritional guidelines for dysgeusia
Educate patients of the need to eat despite symptoms of dysgeusia and/or dysosmia
Encourage consumption of more pleasant foods
Modify food consistency based on acceptance
Increase diet fractionation (6 to 8 meals a day)
Consider association of hypercaloric/hyperproteic oral supplementation (2 to 3 times a day)
Tell the patient to prepare visually pleasing dishes, giving preference to foods with stronger flavor, use drops of lime juice on salads and beverages, herbs, and spices in preparations to enhance taste and recall the taste of foods before ingesting them

Source: Brasil. Ministério da Saúde. Instituto Nacional de Câncer José de Alencar Gomes da Silva INCA; Consenso nacional de nutrição oncológica. 2a. ed. rev. ampl. atual. Rio de Janeiro: INCA, 2015. p.182 [cited 2020 Jul 4]. Available at: https://www.inca.gov.br/sites/ufu.sti.inca.local/files/media/document/consenso-nacional-de-nutricao-oncologica-2-edicao-2015.pdf.^(20)^

The use of lime juice, the intake of candy and sugary juices before meals, the use of plastic utensils and straws, and water rinsing with salt or mouth wash, despite the limited scientific evidence, were also cited as adjuvants in symptom management.^(185)^ Some studies suggest that the use of seasonings such as salt, sodium or potassium glutamate, and sugar may be beneficial in managing dysgeusia.^(186)^ Acupuncture has not shown any benefits.^(187)^

Other empirical therapies have already been suggested, but without proven benefit in trials, such as the use of corticosteroids, vitamin A, *Ginkgo biloba*, glutamine and gabapentin.^(188,189)^ New perspectives include the use of Cannabis (dronabinol) and alpha lipoic acid.^(190–193)^

Some studies, most of which carried out in adults, showed that dysgeusia could last from 1 to 3 years after the end of treatment. A recent study carried out in pediatric patients undergoing HSCT showed resolution of dysgeusia at an average of 6 months after grafting. Discrepancy in the results can be explained by the higher rate of receptor regeneration in children than in adults.^(194)^

### Diarrhea

Diarrheal disease is one of the most frequent gastrointestinal complications of allogenic HSCT and may occur in other types of transplants.^(195)^ Diarrheal disease usually occurs between days 6 and 11 after transplantation, but may be present in all phases of HSCT. Energy intake was decreased in these periods due to nausea, vomiting, diarrhea and loss of appetite.^(196)^ Severe diarrheal disease can be defined as more than six evacuations – according to the Bristol scale, six or seven.^(197)^ Another definition is severe fecal loss with a volume greater than 30mL/kg, corresponding to fecal loss caused by cholera.^(198)^

The causes of diarrheal disease in HSCT can be classified into four large groups, according to table 25.

**Table 25:** Causes of diarrheal disease in pediatric hematopoietic stem cell transplantation

Cause	Mechanism
Treatment/ conditioning	Antibiotics: azathioprine
	Calcineurin inhibitors: cyclosporine, methotrexate, tacrolimus, sirolimus and thiotepa
	Alkaline agents: cyclophosphamide, melphalan and busulfan
	Total body irradiation
Infectious – viruses, bacteria, fungi, and parasites	Bacteria: *Clostridioides difficile, Campylobacter jejuni/coli*, *Listeria monocytogenes*, *Escherichia coli* 0157:H7, *Salmonella species*, *Shigella species*, *Vibrio species* or *Yersinia species* and nocardioses
	Virus: adenovirus, rotavirus, cytomegalovirus and noravirus
	Parasites: giardiasis, *Fusarium* infection, *Microsporidium*, histoplasmosis, and *Candida species*
Umbilical cord colitis syndrome	No association was found with infections or graft *versus* host disease; characterized by granulomatous disease that responds to antibiotic therapy (ciprofloxacin and metronidazole)
Graft *versus* host disease	One of the causes of diarrheal disease in allogeneic transplantation, accompanied by jaundice, skin rash and other gastrointestinal symptoms (abdominal pain and presence of paralytic ileus). It may occur after day 15 of the transplant and is a very likely diagnosis after week 3 of the transplant. One should always rule out other causes of diarrheal diseases before initiating immunosuppressants

Source: Robak K, Zambonelli J, Bilinski J, Basak GW. Diarrhea after allogeneic stem cell transplantation: beyond graft-*versus*-host disease. Eur J Gastroenterol Hepatol. 2017;29(5):495-502. Review;^(199)^ Morishita T, Tsushita N, Imai K, Sakai T, Miyao K, Sakemura R, et al. The efficacy of an oral elemental diet in patients undergoing hematopoietic stem cell transplantation. Intern Med. 2016;55(24):3561-9;^(200)^ Goldberg RJ, Nakagawa T, Johnson RJ, Thurman JM. The role of endothelial cell injury in thrombotic microangiopathy. Am J Kidney Dis. 2010;56(6):1168-74;^(201)^ Shah NN, McClellan W, Flowers CR, Lonial S, Khoury H, Waller EK, et al. Evaluating risk factors for clostridium difficile infection in stem cell transplant recipients: a national study. Infect Control Hosp Epidemiol. 2017;38(6):651-7;^(202)^ Webb BJ, Brunner A, Ford CD, Gazdik MA, Petersen FB, Hoda D. Fecal microbiota transplantation for recurrent Clostridium difficile infection in hematopoietic stem cell transplant recipients. Transpl Infect Dis. 2016;18(4):628-33;^(203)^ Kwak KS, Zhou X, Solomon V, Baracos VE, Davis J, Bannon AW, et al. Regulation of protein catabolism by muscle-specific and cytokine-inducible ubiquitin ligase E3alpha-II during cancer cachexia. Cancer Res. 2004;64(22):8193-8;^(204)^ Lee JL, Leong LP, Lim SL. Nutrition intervention approaches to reduce malnutrition in oncology patients: a systematic review. Support Care Cancer. 2016;24(1):469-80. Review;^(205)^ Watson T, MacDonald D, Song X, Bromwich K, Campos J, Sande J, et al. Risk factors for molecular detection of adenovirus in pediatric hematopoietic stem cell transplantation recipients. Biol Blood Marrow Transplant. 2012;18(8):1227-34;^(206)^ Srinivasan A, Klepper C, Sunkara A, Kang G, Carr J, Gu Z, et al. Impact of adenoviral stool load on adenoviremia in pediatric hematopoietic stem cell transplant recipients. Pediatr Infect Dis J. 2015;34(6):562-5;^(207)^ César Pereira Santos H, Nunes Vieira Almeida T, Souza Fiaccadori F, das Dôres de Paula Cardoso D, de Moraes Arantes A, Delleon da Silva H, et al. Adenovirus infection among allogeneic stem cell transplant recipients. J Med Virol. 2017;89(2):298-303;^(208)^ Williams D. Treatment of rotavirus-associated diarrhea using enteral immunoglobulins for pediatric stem cell transplant patients. J Oncol Pharm Pract. 2015;21(3):238-40;^(209)^ Patel NC, Hertel PM, Hanson IC, Krance RA, Crawford SE, Estes M, et al. Chronic rotavirus infection in an infant with severe combined immunodeficiency: successful treatment by hematopoietic stem cell transplantation. Clin Immunol. 2012;142(3):399-401;^(210)^ Liu A, Meyer E, Johnston L, Brown J, Gerson LB. Prevalence of graft *versus* host disease and cytomegalovirus infection in patients post-haematopoietic cell transplantation presenting with gastrointestinal symptoms. Aliment Pharmacol Ther. 2013;38(8):955-66;^(211)^ Ye X, Van JN, Munoz FM, Revell PA, Kozinetz CA, Krance RA, et al. Noroviruses as a cause of diarrhea in immunocompromised pediatric hematopoietic stem cell and solid organ transplant recipients. Am J Transplant. 2015;15(7):1874-81;^(212)^ Ueda R, Fuji S, Mori S, Hiramoto N, Hashimoto H, Tanaka T, et al. Characteristics and outcomes of patients diagnosed with norovirus gastroenteritis after allogeneic hematopoietic stem cell transplantation based on immunochromatography. Int J Hematol. 2015;102(1):121-8;^(213)^ Troeger H, Loddenkemper C, Schneider T, Schreier E, Epple HJ, Zeitz M, et al. Structural and functional changes of the duodenum in human norovirus infection. Gut. 2009;58(8):1070-7;^(214)^ Saif MA, Bonney DK, Bigger B, Forsythe L, Williams N, Page J, et al. Chronic norovirus infection in pediatric hematopoietic stem cell transplant recipients: a cause of prolonged intestinal failure requiring intensive nutritional support. Pediatr Transplant. 2011;15(5):505-9;^(215)^ Ajumobi AB, Daniels JA, Sostre CF, Trevino HH. Giardiasis in a hematopoietic stem cell transplant patient. Transpl Infect Dis. 2014;16(6):984-7;^(216)^ Bouanani N, Lamchahab M, Quachouh M, Soussi M, Quessar A, Benchekroun S. [Disseminated fusariosis during autologous stem cells transplant]. J Mycol Med. 2013;23(2):119-22. French;^(217)^ Krones E, Högenauer C. Diarrhea in the immunocompromised patient. Gastroenterol Clin North Am. 2012;41(3):677-701. Review;^(218)^ Milano F, Shulman HM, Guthrie KA, Riffkin I, McDonald GB, Delaney C. Late-onset colitis after cord blood transplantation is consistent with graft-*versus*-host disease: results of a blinded histopathological review. Biol Blood Marrow Transplant. 2014;20(7):1008-13;^(219)^ Gupta NK, Masia R. Cord colitis syndrome: a cause of granulomatous inflammation in the upper and lower gastrointestinal tract. Am J Surg Pathol. 2013;37(7):1199-13.^(220)^ Ball LM, Egeler RM; EBMT Paediatric Working Party. Acute GvHD: pathogenesis and classification. Bone Marrow Transplant. 2008;41(Suppl 2):S58-64. Review.^(221)^Kambham N, Higgins JP, Sundram U, Troxell ML. Hematopoietic stem cell transplantation: graft *versus* host disease and pathology of gastrointestinal tract, liver, and lung. Adv Anat Pathol. 2014;21(5):301-20. Review. ^(222)^

The impairment of intestinal structures, dysfunction of organs responsible for nutrient digestion, and the use of intestinal microbiota-modifying drugs contribute to the genesis of diarrheal disease. Villus injury favors the impairment of lactase activity, leading to different degrees of lactose intolerance. Moreover, rupture of the intestinal barrier facilitates the exposure of macroproteins, which can cause multiple allergies, such as cow’s milk, soy and gluten.^(199)^

Infectious etiology of diarrheal disease should always be ruled out since it is very frequent in immunosuppressed patients and in HSCT.

An algorithm to evaluate the causes of diarrheal disease in allogeneic HSCT was proposed by Robak et al.,^(199)^ Infectious causes should be ruled out first; then, GVHD should be ruled out and, finally, other causes.

In patients with acceptance of oral diet reaching 80% of energy and micronutrient requirements, it is recommended to monitor acceptance and advise on dietary supplementation.^(223)^ The diet offered should be low residue. In addition, meal food fractionation should be advised, with six to eight portions per day, ensuring adequate hydration for the age group. The acceptance of some gastric irritant foods, such as deep-fried foods or sweets, and specific nutrients such as lactose, sucrose, gluten and caffeine, should be assessed individually, and if necessary, intake can be restricted if diarrhea occurs.^(124)^

Enteral nutrition using nasogastric or post-pyloric tubes is required in patients with nutritional impairment whose oral intake does not reach 70% to 80% of energy and micronutrient requirements, and diet administration and the type of protein used (polymeric or extensively hydrolyzed diet) should be monitored. The tube should be passed at the onset of conditioning until the first week after transplantation, when oral intake is compromised. Enteral diet infusion should always be performed with an infusion pump, aiming to control infused volume.^(224)^ If the enteral nutrition volume progression does not reach 70% of the basal metabolic rate, or after 3 days of fasting, parenteral nutrition is considered.

### Nutritional intervention

According to the guidelines for nutritional therapy during HSCT, all patients undergoing this procedure with myeloablative conditioning regimes are at nutritional risk.^(76,95,225)^

Nutritional follow-up is important at all treatment phases, and it is possible to adequately identify nutritional risk and implement nutritional therapy as early as possible, according to the conditions and needs of children and adolescents, meeting criteria that ensure the best decision, increasing the benefits of this support and avoiding the risks of inadequate indication.^(76,95,225)^

The general objective of nutritional therapy is to improve treatment response, reduce the risk of complications and optimize survival and quality of life.

The goals of nutritional therapy can be separated by treatment phase, according to table 26.

**Table 26:** Nutritional goals according to treatment phases

Treatment phase	Nutritional goal
Pre-HSCT	Correct or maintain adequate nutritional status by correcting macro and micronutrient reserves to improve treatment tolerance
	Reduce risk of infection
	Improve immunity and response to inflammation during treatment
Hospitalization for HSCT (conditioning, cell infusion and bone marrow engraftment)	Minimize nutritional aggravation
	Control gastrointestinal symptoms
	Improve treatment response
	Decrease general complications: special attention to weight gain that may indicate complications, such as SOS or nephrotoxicity
	Minimize growth and development deficit
	Control pro-inflammatory response
	Shorten the length of hospital stay
Post-HSCT	Maintain an appropriate growth and development curve
	Correct nutritional status
	Control nutritional and metabolic repercussions

Source: Garófolo A, Lopez FA, Petrilli AS. High prevalence of malnutrition among patients with solid non-hematological tumors as found by using skinfold and circumference measurements. Sao Paulo Med J. 2005;123(6):277-81;^(42)^ Papadopoulou A, Williams MD, Darbyshire PJ, Booth IW. Nutritional support in children undergoing bone marrow transplantation. Clin Nutr. 1998;17(2):57-63;^(69)^ Christensen ML, Hancock ML, Gattuso J, Hurwitz CA, Smith C, McCormick J, et al. Parenteral nutrition associated with increased infection rate in children with cancer. Cancer. 1993;72(9):2732-8;^(226)^ Dioguardi J, Bryson E, Ahmed-Winston S, Vaughn G, Slater S, Driscoll J, et al. A multi-institutional retrospective study suggests that optimal enteral nutrition (EN) influences outcomes after hematopoietic stem cell transplantation in children and adults. Biol Blood Marrow Transplant. 2015;21(2):S248-9;^(227)^ Bechard LJ, Guinan EC, Feldman HA, Tang V, Duggan C. Prognostic factors in the resumption of oral dietary intake after allogeneic hematopoietic stem cell transplantation (HSCT) in children. JPEN J Parenter Enteral Nutr. 2007;31(4):295-301;^(228)^ Carr SE, Halliday V. Investigating the use of the neutropenic diet: a survey of U.K. dietitians. J Hum Nutr Diet. 2015;28(5):510-5.^(229)^

HSCT: hematopoietic stem cell transplantation; SOS: sinusoidal obstruction syndrome.

### Nutritional therapy

Food acceptance may be affected by the numerous side effects of HSCT. The diet offered should meet the needs of the current moment of the patient. In this context, two topics related to oral nutritional therapy are worth highlighting: neutropenic diet and oral supplementation.

### Neutropenic diet

After HSCT conditioning, immunity is impaired, and there is a period of neutropenia and high susceptibility to infections. Historically, dietary restrictions known as “clean”, “low bacteria,” “microbial” or “neutropenic” diets have been prescribed to reduce the risk of foodborne infection.^(229)^

Neutropenia is defined as a total neutrophil count under 500/mm³ or expected to decrease below that level in the next 48 hours.^(230)^ The duration of neutropenia varies according to the type of HSCT and the presence or absence of complications, such as GVHD and bone marrow engraftment failure.^(231)^

After the preparative regimen, during neutropenia, the patient may have mucosal damage throughout the GIT, caused by conditioning, which may be an inlet for Gram-negative pathogens, such as *Pseudomonas aeruginosa*, *Escherichia coli*, *Klebsiella* and *Proteus*, in different foods. When foods are cooked, baked, fried or heated, the number of microorganisms can be reduced considerably, and there is a strong recommendation for diets with cooked foods during the period of bone marrow aplasia.^(232)^

The HSCT diet is based on food safety, and aims to prevent foodborne infections and bacterial translocation, during aplasia and bone marrow fragility. Nutritional advice should be focused on good hygiene and food preparation practices, for both training of staff handling patient meals during hospitalization and guiding children’s caregivers after discharge. In addition, some foods must be excluded from the diet because of their increased risk of containing pathogens.^(99)^

The foods with the highest risk for patients are raw eggs, non-pasteurized dairy products, fruits, vegetables and legumes without proper washing, among others (Table 27).^(99)^

**Table 27:** Dietary guidelines for hematopoietic stem cell transplantation

Restrict consumption
Water and ice of doubtful origin, unfiltered or unboiled
Raw or undercooked meats (beef, pork, chicken, and lamb)
Raw or uncooked eggs or preparations containing them
Unpasteurized fresh milk and dairy products (cheese, butter, and yogurt)
Probiotic-containing foods
Raw vegetables without adequate washing
Raw cereals
Raw homemade honey exempt from federal inspection stamp
Homemade preserves exempt from federal inspection stamp (*e.g.*, canned hearts of palm, preserved olives, etc.)
Raw tofu
Sugarcane juice
Acai berry, natural fruit
Unprocessed foods ready for consumption, purchased in restaurants, burger joints, bakeries etc.

Consume according to preparation and hygiene guidelines
Raw, thin-skinned and/or hard-to-wash fruits (*e.g.*, blackberry, strawberry, grapes, cherry, jabuticaba and blueberry)
Raw, non-intact or unwashed medium-to-thick-skinned fruits (*e.g.*, avocado, apple, mango, papaya, persimmon, watermelon, pineapple, melon, banana, tangerine, orange, passion fruit, lime, pear, guava and plum)
Raw dried fruits and oil seeds
Uncooked cheese
Uncooked sausages and deli meats (*e.g.*, hot dogs, salami, ham etc.)
Uncooked dried herbs and spices

A survey of different professionals who used this diet found that there is a range of restricted foods, and contradictions are frequent. The most frequently restricted foods were seafood and fish.^(229)^

The HSCT diet or neutropenic diet starts at conditioning and continues until the withdrawal of immunosuppressants.^(99)^ Some Brazilian authors recommend that the neutropenic diet be extended up to 100 days after allogeneic HSCT or 60 days for autologous. However, other authors suggest that, for allogeneic HSCT, the diet should continue until withdrawal of all immunosuppressants and, in autologous HSCT, up to 1 month after discontinuation of corticosteroids or 3 months after complete resolution of gastrointestinal lesions.^(233)^ The timing of diet suspension is not yet a consensus among reference centers.

Differences in practice can be attributed to the lack of robust, high-quality evidence.^(229)^ Perhaps preparation, storage and quality of raw materials are more important than the food type restricted, considering the reality of the hospital and population served.

Thus, the role of the neutropenic diet in the incidence of infections in HSCT is still controversial.^(234)^ Studies are limited, either in pediatric or adult populations. Another bias is that there are few studies portraying the reality of our population. Further studies are required, not only high-quality, but also studies that truly portrait the reality of our population.

### Oral supplements (homemade or commercial)

The indication of oral nutritional supplements should be assessed individually and occur when food intake is <70% to 80% of nutritional recommendations for 3 to 5 consecutive days, considering the level of nutritional risk; the expected time of improvement in food intake and the predicted grafting time; GIT and conditioning.^(235–237)^ Therefore, it is recommended that food intake calculations be performed on a daily basis.

Supplements may be commercial or homemade.

The decision to initiate oral supplementation must take some criteria into account: reduced food intake, regardless of other indicators; risk of malnutrition, meaning any weight loss or deceleration in the growth curve; reduced fat reserves or muscle mass; gastrointestinal abnormalities, regardless of other indicators; and patient submitted to HSCT, regardless of other conditions.

When choosing which supplement to use, some criteria should also be considered, such as age group; taste, form, and quantity; nutritional and catabolic status (normo or hypercaloric and/or protein supplements may be necessary); GIT (hydrolyzed or otherwise modified supplements may be required); metabolic condition (changes in serum glucose or lipids; systemic inflammation); comorbidities (liver, kidney, pancreatic abnormalities, among others) and socioeconomic situation.

Commercial supplements can help with nutritional therapy, but their high price makes them difficult to buy, and limit their widespread use. Furthermore, there are not many oral supplement options for the pediatric age group, which warrants the use of homemade alternatives using attractive preparations, with ingredients that can provide higher energy-protein density and micronutrients.

Thus, homemade supplements obtained by homemade modulation of dietary ingredients can be a less expensive option than commercial products. Nevertheless, there are some disadvantages, when comparing homemade and commercial supplements, such as poorer microbiological control and greater manipulation, more complex preparation, and difficulty ensuring equal nutritional value.^(238,239)^

Maia et al.^(240)^ showed that oral supplementation, whether commercial or homemade, can prevent further deterioration of nutritional status, particularly in patients with good compliance with nutritional appointments and following guidance. However, commercial supplements seem to elicit a favorable response in a higher percentage of patients.

Usually, the presence of more severe malnutrition requires other nutritional therapy measures, since patients in these circumstances have more difficulty meeting requirements by oral feeding.^(238,239)^

Thus, the use of oral supplements can be a viable alternative to prevent nutritional loss, as well as more severe thinness and sarcopenia. Commercial supplements ensure a homogeneous supply of nutrients and are more practical for routine preparation. However, their taste can be unappealing to children. The combination of homemade and commercial supplements is an important strategy for preventing malnutrition. When resources are too limited for commercial supplements, homemade supplements should be recommended.

Often, protein intake can be impaired, which tends to intensify catabolism and sarcopenia. Protein requirements in these patients are high, and the use of commercial protein supplements or homemade supplementation strategies are required to achieve the necessary supply.

The initial prescription of this therapy should account for 45% to 50% of the patients’ nutritional requirements for effective nutritional contribution. Programmed weaning from oral supplements can be carried out when oral intake rises to ≥70% to 80% of the calculated nutritional requirements for 2 or 3 days, until it reaches 100% of requirements without oral supplements.^(240)^

### Enteral nutrition

For ENT, the functional capacity of the GIT tract must be evaluated. Situations that modify digestion and absorption systems, such as mucositis, infections, GVHD, among others, may compromise the proper uptake of nutrients and be inefficient, if poorly indicated.^(241,242)^

Based on the concept that prolonged fasting causes atrophy of the intestinal mucosa, breaking the immunological integrity of the GIT and increasing the risk of bacterial translocation, food is an important stimulus to maintain the function and structure of the intestinal mucosa, leading to the release of pancreatic secretions, bile and hormonal factors.^(241,242)^

This route of nutritional support must be prioritized in patients with a functioning or partially functioning GIT, before an indication for parenteral nutrition, since it preserves the tropism of the intestinal mucosa.^(243)^

Langdana *et al.*,^(244)^ observed that nutritional therapy was feasible through an intensive enteral nutrition program in the pediatric population undergoing HSCT, including patients receiving TBI conditioning.

Another Brazilian study observed that the use of tube nutrition in children and adolescents with cancer during HSCT is feasible, and there were no severe complications associated with therapy. Minor complications occurred in 55% of patients, namely: more intense episodes of vomiting or diarrhea as diet volume increased (16%), displaced tube (19%), fungal infection in the oral cavity (9.7%) and obstructed tube (6.5%).^(242)^

Some trials have considered enteral nutrition as effective as parenteral nutrition, but with lower complication rates. In addition, enteral nutrition was associated with better survival, lower incidence of aGVDH and cGVDH, and faster neutrophil recovery rate associated with lower risk of infection.^(227,243,245,246)^

Data from another study suggest that nutritional therapy in both pre- and during HSCT correlated with better nutritional recovery after HSCT.^(227)^

ENT has been widely recommended for pediatric patients undergoing HSCT, and enteral tube feeding is the preferential route, in the absence of severe GIT toxicity.^(244,247,248)^

ENT through feeding tube or stoma is indicated when oral feeding is not possible (grade 1 and 2 mucositis), when food intake is insufficient (oral intake <70-80% of requirements) for 3 to 5 consecutive days, with insufficient food and supplements (below 100% of basal energy requirements), associated with undernutrition or weight loss, considering a high-risk nutritional status, expected improvement time and estimated time for grafting, severe undernutrition or impossibility of oral feeding.^(41,240)^ For this purpose, it is suggested that daily dietary intake calculations be performed to determine whether ENT is indicated.

Delayed indication of nutritional therapy may make it difficult to use tube feeding and predispose to a higher risk of complications. Thus, a greater number of patients will benefit from early indication of tube feeding, which reduces need for parenteral nutrition or at least the duration and risks of this type of therapy.

The treatment phase, presence of gastrointestinal toxicity, clinical condition and current nutritional status must be considered for enteral nutrition weaning. Programmed weaning from tube feeding can be initiated when oral intake reaches ≥70% to 80% of nutritional requirements calculated for 2 or 3 days.

### Parenteral nutrition

Historically, total parenteral nutrition was the most commonly used method to provide nutrients during HSCT. The importance of nutrition, especially parenteral nutrition, was clearly shown in the results of the randomized study by Weisdorf *et al.*,^(249)^ demonstrating that the administration of prophylactic parenteral nutrition during the course of HSCT increased survival in the treated group, after 3 years of follow-up.

However, the use of parenteral nutrition is also associated with an increased risk of complications, especially infectious and metabolic complications, particularly among patients with severe immunosuppression, such as patients undergoing HSCT.

Despite evidence of positive nutritional results with total parenteral nutrition in children and adolescents during HSCT,^(250)^ there are few studies in this context, and information on the effects of total parenteral nutrition in this population is limited. The recommendations are based on results of the studies already discussed here, including data on adults, which also support the principles of nutritional therapy in children and adolescents with cancer.

In pediatric oncology, some diagnoses, and neoplastic agents, such as chemotherapy with thiotepa, melphalan and cisplatin, and total body irradiation, will make patients much more likely to require parenteral nutrition.

Some important aspects related to parenteral nutrition include monitoring and metabolic control of supply and the type of catheter used. Since there is a great risk of metabolic changes due to the inflammatory process and infections due to catheter manipulation, special attention should be given while managing this therapy.

The main indications for parenteral nutrition include total or partial impossibility of using the GIT: severe thrombocytopenia not resolved after platelet infusion in patients on enteral therapy, and difficulty achieving nutritional requirements by full enteral nutrition within 5 days, considering the nutritional status and estimated time until grafting.

Finally, the routine use of parenteral nutrition is not recommended unless GI toxicity or serious GI complications prevent full enteral feeding.

### Nutritional therapy algorithms

Defining criteria for nutritional therapy decision-making, improving processes, and ensuring that adequate therapy yields more benefits than complications is fundamental for the therapeutic planning of patients.

**Figure 2:**
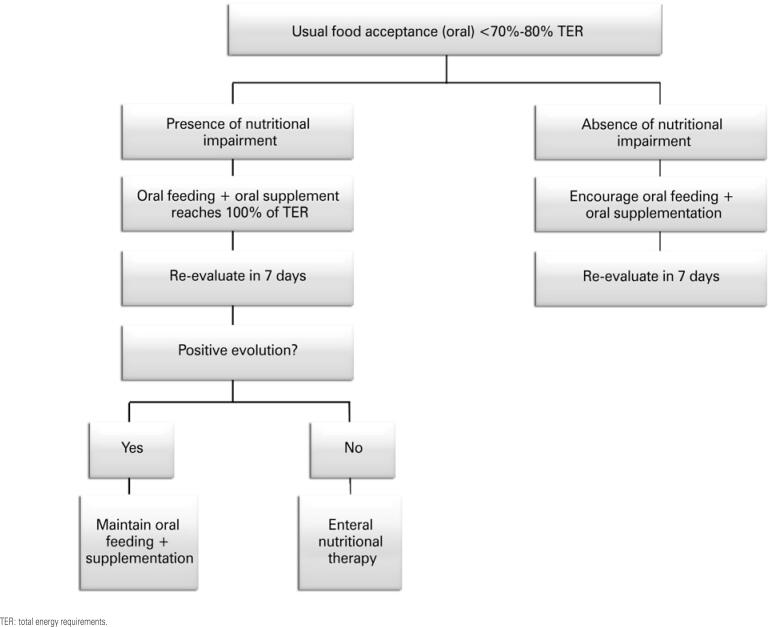
Nutritional therapy algorithm in pediatric patients pre-hematopoietic stem cell transplantation

**Figure 3:**
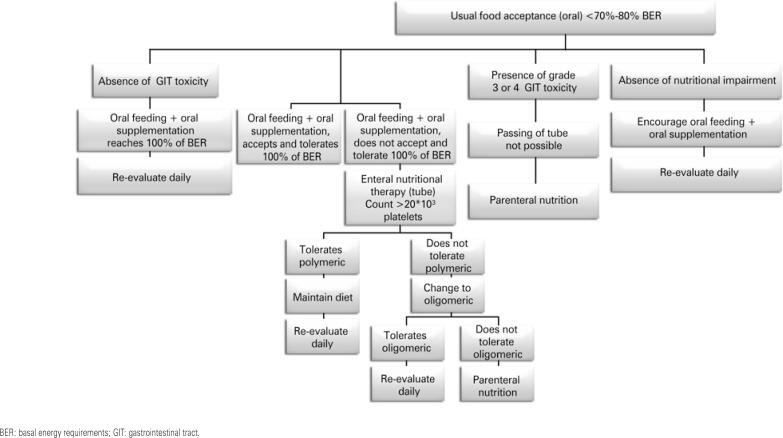
Nutritional therapy algorithm in pediatric patients undergoing hematopoietic stem cell transplantation

**Figure 4:**
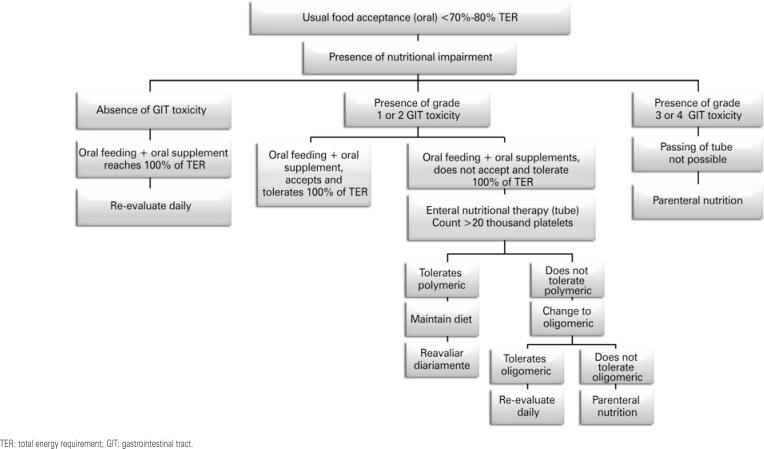
Nutritional therapy algorithm in pediatric patients post-hematopoietic stem cell transplantation

According to a survey on nutritional therapy routes, the following algorithms are suggested, separated by HSCT phase: nutritional therapy algorithm in pediatric patients pre-HSCT (Figure 2), nutritional therapy algorithm in pediatric patients undergoing HSCT (Figure 3), and nutritional therapy algorithm in pediatric patients post-HSCT (Figure 4).

### Use of probiotics

Recently, the intestinal microbiota trajectory has been studied in children during HSCT, as well as its role in aGVHD, and it has been demonstrated that intestinal microbial diversity can predict survival in these patients. HSCT causes temporary structural and functional changes to the intestinal microbiota ecosystem, which shows signs of recovery at 100 days after transplantation and aggravates in case of acute GVHD.^(251–253)^ Italian authors reported the association of acute GVHD with specific bacteria, both during restoration of the intestinal flora immediately after HSCT, curiously, even before the transplantation. In a study published in 2015, pre-transplant samples of patients without acute GVHD showed greater presence of a type of propionate-producing bacterium, which persisted even after disruption of the ecosystem by the transplant. These results suggest the possibility of manipulating the pre-HSCT intestinal microbiota configuration to favor the success of the procedure.^(252)^

The use of probiotics during and immediately after HSCT is still challenged in clinical practice, due to very few studies demonstrating its safety, especially in pediatric patients, and to the supposedly high risk of bacterial translocation in connection with structural and immunological weakening of the intestinal barrier.^(254–256)^ An American study published in 2016^(257)^ demonstrated that the use of *Lactobacillus plantarum* is safe and feasible during neutropenia in children and adolescents undergoing myeloablative HSCT, and was the first clinical trial to use probiotics in this population, which points to promising results with the aforementioned bacterial strain in this population.

Currently, and until there are further studies demonstrating the safety and benefits of the use of probiotics in children and adolescents undergoing HSCT, this consensus contraindicates this approach in this situation.

### Use of glutamine

Glutamine is important for physiological functions, serving as a precursor in the synthesis of other amino acids, such as arginine, and acting as an important antioxidant and fuel for the rapid cellular proliferation of the GIT, immune system, reticulocytes and fibroblasts, mainly in the formation and maintenance of the intestinal mucosal barrier.^(258)^

It is naturally found in dietary protein and considered non-essential; however, in stress situations, it becomes a conditionally essential nutrient, *i.e.*, in situations of increased metabolic demand, such as catabolism, serious disease and prematurity, the body cannot produce enough.^(259)^

Administration in the form of supplements can be either orally or intravenously.

As for intravenous glutamine, to date, there is not enough evidence to recommend its use; on the contrary, it is not recommended due to toxicity reports.^(260)^ Since the release of last two position papers about this subject, two new randomized, controlled studies have been published, however with a small number of patients.^(261,262)^ They showed positive effects of intravenous glutamine on intestinal permeability and chemotherapy-induced mucosal injury. Further evaluations on the subject are expected before any future changes in the recommendations.^(263)^

Few studies have assessed the use of oral/enteral glutamine in children. Ward *et al.*,^(264)^ followed 76 pediatric patients on chemotherapy divided into two groups, one of them receiving once-daily 0.65g/kg oral or enteral glutamine diluted in water, starting on day 1 of chemotherapy. The study concluded that glutamine did not reduce the incidence and severity of oral mucositis but showed a reduction in the number of patients on parenteral nutrition.^(264)^ A systematic review in 2016,^(265)^ aiming to investigate the role of oral glutamine in preventing mucositis in adult patients undergoing radiotherapy and/or chemotherapy, observed a significant reduction in the severity of lesions, with faster resolution and less weight loss in those who received the amino acid. Maximum doses of 30g per day were used, with no difference in side effects compared with the control group with no glutamine. For greater efficacy, oral glutamine should be started zero to 7 days before radiation/chemotherapy, divided in three administrations. The authors drew attention to some limitations regarding the methodology of the studies, such as the small number of patients, non-homogeneous sample, and retrospective design. This made it difficult to identify which patients could benefit from oral glutamine and at which stage of the treatment.^(263,266)^ Further studies will help define whether oral glutamine could be effective for all types of cancer and as an adjuvant to anti-cancer drugs.

There is no consensus on the dose and route of administration for this amino acid. The doses ranged from 7.5g to 30g per day before treatment or fractionated into three times a day. The timing also varied in different studies, either before, during or after the chemotherapy cycle.^(267)^

According to the bibliographic reviews performed, table 28 shows the indications for glutamine in cases of mucositis and/or intestinal tropism.

**Table 28:** Summary of glutamine indications and routes of administration

Glutamine	Mucositis	Intestinal tropism
Intravenous	It is recommended not to use intravenous glutamine to prevent oral mucositis in patients on high-dose chemotherapy, with or without TBI, for HSCT	However conflicting, recent studies show promising results. Further evaluations are needed. Currently not recommended
Oral/enteral	Could be a viable and low-toxicity option. The lack of consensus regarding the dose, route and timing makes it difficult to standardize its use	No evidence

Source: Arends J, Bachmann P, Baracos V, Barthelemy N, Bertz H, Bozzetti F, et al. ESPEN guidelines on nutrition in cancer patients. Clin Nutr. 2017;36(1):11-48;^(225)^ Lalla RV, Bowen J, Barasch A, Elting L, Epstein J, Keefe DM, McGuire DB, Migliorati C, Nicolatou-Galitis O, Peterson DE, Raber-Durlacher JE, Sonis ST, Elad S; Mucositis Guidelines Leadership Group of the Multinational Association of Supportive Care in Cancer and International Society of Oral Oncology (MASCC/ISOO). MASCC/ISOO clinical practice guidelines for the management of mucositis secondary to cancer therapy. Cancer. 2014;120(10):1453-61;^(260)^ Ward E, Smith M, Henderson M, Reid U, Lewis I, Kinsey S, et al. The effect of high-dose enteral glutamine on the incidence and severity of mucositis in pediatric oncology patients. Eur J Clin Nutr. 2009;63(1):134-40^(264)^ Storey B. The role of oral glutamine in pediatric bone marrow transplant. J Pediatr Oncol Nurs. 2007;24(1):41-5;^(268)^

TBI: total body irradiation; HSCT: hematopoietic stem cell transplantation.

### Micronutrient supplementation

The optimum use of nutrients requires other substances called micronutrients. These substances are required in minimal quantities, proportionally low when compared to their great influence on metabolism and health. Micronutrients are of two types: trace-elements and vitamins. The former are inorganic elements, while vitamins are complex organic molecules. Trace elements are an essential and integral part of enzymes, of which seven are essential for human health: iron, zinc, copper, chrome, selenium, iodine and cobalt.^(269)^ Vitamins are usually converted in the body into complex molecules that function as coenzymes with various roles in metabolism, which cannot be synthesized and need to be provided by the diet.^(269)^

Vitamin and mineral supplementation is important during all phases of HSCT, since patients have long-term feeding difficulties, aggravated by dietary restrictions imposed by the neutropenic diet, and the requirements for some vitamins and minerals are increased.^(58)^ In addition, children’s eating habits should be considered, which, if inadequate in the long term, may lead to micronutrient deficiency. In the last decades, a higher consumption of processed foods with high caloric density and low essential nutrients has been observed in the Brazilian population, replacing the consumption of more nutritious foods, such as fruits, vegetables, and legumes. As a result of changes in the eating pattern, vitamin supplements and/or enriched foods serve as practical vitamin carriers.^(223)^

There are few studies evaluating serum concentrations of vitamins and minerals in children undergoing HSCT. Recent studies in adults found vitamin B1 deficiency in the pre-HSCT phase, in addition to vitamin C and zinc deficiency in the post-HSCT phase.^(270)^ Some nutrients with antioxidant properties, such as vitamins C and E and beta-carotene, are depleted after HSCT, as well as vitamin B1 and zinc,^(59,270)^ and patients presented oxidative stress during conditioning, particularly on the cyclophosphamide + TBI regimen.^(271)^

Patients undergoing allogeneic HSCT are also at increased risk of thiamine (vitamin B1) deficiency, leading to metabolic changes, such as lactic acidosis,^(272)^ and neurological changes, such as Wernicke encephalopathy, and should be supplemented in doses higher than the RDA during the immediate post-HSCT period. Absorption of vitamin B12 is also reduced due to the effects of conditioning or as a result of GVHD.^(82,273,274)^ A Brazilian study involving children and adolescents undergoing allogeneic HSCT found a high prevalence of vitamin D deficiency as early as pre-HSCT, with reduction in serum levels after 6 months.^(152,275)^ Studies in adults have suggested that the immunomodulatory properties of this vitamin could play an important role in the prevention and treatment of GVHD.^(276)^

Lipid oxidation can be inhibited with supplementation of vitamin C associated with vitamin E (800IU to 1,000IU) and carotenoid (45mg).^(59)^ Another advantage of vitamin E supplementation would be prevention of hepatic VOD.^(59)^ Vitamin C should also be supplemented to promote tissue recovery through post-conditioning collagen biosynthesis, with a daily recommendation of 250mg for patients below 31kg and 500mg for patients over 31kg, with contraindication when serum ferritin is greater than 1,000mg/L.^(277)^ Vitamin K supplementation is also necessary; during the HSCT process, this deficiency is related to the use of some medications. During the conditioning period, many children receive phenytoin as prophylaxis for seizures, and this medication is a vitamin K antagonist.^(277)^ Furthermore, some antibiotics, such as cephalosporins, can directly inhibit the hepatic epoxide-reductase enzyme, also antagonizing this vitamin.^(278)^

The recommendation for patients undergoing HSCT is to use supplements containing all vitamins and minerals, such as calcium, zinc, selenium, and iron-free, respecting the DRIs and for, at least 1 year after HSCT.^(276)^ Iron supplementation is generally not recommended because most patients have high iron concentrations due to frequent blood transfusions.^(277)^ There are few options for pediatric-specific multivitamin supplements on the Brazilian market, and, alternatively, individualized, compounded formulas may be used.

In some special situations, supplemental micronutrients should be reviewed and individualized. Patients with grade 3 and 4 skin aGVHD should receive extra doses of some vitamins and minerals to promote tissue healing and recovery,^(277)^ and the nutrient requirements can be compared to those of major burn patients.

In cases of voluminous diarrhea, additional zinc supplementation is needed to restore increased losses, at a dose of 10mg to 12mg per liter of feces.^(223,269,277)^ We utilize vitamins and trace elements formulations that are not prepared specifically for patients receiving HSCT. We should remember that zinc is an essential trace-element, important for growth, wound healing, immune system maintenance and other vital functions, and should be added to all total parenteral nutrition at a dose of 100mcg/kg for children and 3mg to 4mg for adolescents.^(269)^ In patients with liver dysfunction (with bilirubin >10mg/dL), caution is advised when adding copper and magnesium to total parenteral nutrition.^(278,279)^

## CONCLUSION

Pediatric patients undergoing hematopoietic stem cell transplantation may develop numerous complications during and after the procedure. This document aims to empower support teams in the decision-making process for appropriate nutritional therapy to patients, considering basic needs of the age group, as well as current specific needs.

The performance of a thorough nutritional assessment (food history, anthropometric evaluation, laboratory tests and physical examination) before (identifying nutritional deficiencies and programming initial nutritional therapy), during (monitoring complications and programming strategies for nutritional intervention) and after hospital discharge, and during outpatient follow-up (monitoring of dietary intake, symptoms and ensuring age-appropriate diet) creates a surveillance network for potential nutritional complications.
