# Supplementary material for: Brazilian Nutritional Consensus in Hematopoietic Stem Cell Transplantation: children and adolescents
Source: Einstein (Sao Paulo). 2021 Nov 25;19:eAE5254. doi: 10.31744/einstein_journal/2021AE5254 (PMC8664291; doi:10.31744/einstein_journal/2021AE5254)
Supplement: Translation

# Translation: Consenso Brasileiro de Nutrição em Transplante de Células-Tronco Hematopoiéticas: crianças e adolescentes

**Authors:** Juliana Moura Nabarrete, Andrea Z Pereira, Adriana Garófolo, Adriana Seber, Angela Mandelli Venancio, Carlos Eduardo Setanni Grecco, Carmem Maria Sales Bonfim, Claudia Harumi Nakamura, Daieni Fernandes, Denise Johnsson Campos, Fernanda Luisa Ceragioli Oliveira, Flávia Krüger Cousseiro, Flávia Feijó Panico Rossi, Jocemara Gurmini, Karina Helena Canton Viani, Luciana Fernandes Guterres, Luiz Fernando Alves Lima Mantovani, Luiz Guilherme Darrigo, Maria Isabel Brandão Pires e Albuquerque, Melina Brumatti, Mirella Aparecida Neves, Natália Duran, Neysimelia Costa Villela, Victor Gottardello Zecchin, Juliana Folloni Fernandes

**Affiliations:** 1 São Paulo SP Brasil Hospital Israelita Albert Einstein, São Paulo, SP, Brasil.; 2 São Paulo SP Brasil Instituto de Oncologia Pediátrica, Universidade Federal de São Paulo, São Paulo, SP, Brasil.; 3 São Paulo SP Brasil Universidade Federal de São Paulo, São Paulo, SP, Brasil.; 4 Ribeirão Preto SP Brasil Hospital das Clínicas, Faculdade de Medicina de Ribeirão Preto, Universidade de São Paulo, Ribeirão Preto, SP, Brasil.; 5 Curitiba PR Brasil Hospital de Clínicas, Universidade Federal do Paraná, Curitiba, PR, Brasil.; 6 Porto Alegre RS Brasil Santa Casa de Misericórdia de Porto Alegre, Porto Alegre, RS, Brasil.; 7 São Paulo SP Brasil Escola Paulista de Medicina, Universidade Federal de São Paulo, São Paulo, SP, Brasil.; 8 São Paulo SP Brasil Instituto da Criança, Hospital das Clínicas, Faculdade de Medicina, Universidade de São Paulo, São Paulo, SP, Brasil.; 9 Rio de Janeiro RJ Brasil Instituto Nacional de Câncer José Alencar Gomes da Silva - INCA, Rio de Janeiro, RJ, Brasil.; 10 Barretos SP Brasil Hospital de Câncer de Barretos, Barretos, SP, Brasil.

**Keywords:** Avaliação nutricional, Estado nutricional, Pediatria, Criança, Adolescente, Transplante de células-tronco hematopoéticas, Terapia nutricional

## Abstract

O Consenso Brasileiro de Nutrição em Transplante de Células-Tronco Hematopoiéticas: crianças e adolescentes foi elaborado com a participação de nutricionistas, médicos nutrólogos e médicos hematologistas pediátricos de 10 centros brasileiros que são referência em transplante de células-tronco hematopoiéticas. O objetivo foi salientar a importância do estado nutricional e da composição corporal durante o tratamento, bem como as principais características relacionadas à avaliação nutricional do paciente. As intenções, ao se estabelecer o consenso, foram aprimorar e padronizar a terapia nutricional durante o transplante de células-tronco hematopoiéticas. O consenso foi aprovado pela Sociedade Brasileira de Transplante de Médula Óssea.

## INTRODUÇÃO

### Tipos de transplante de células-tronco hematopoiéticas na infância e sua aplicação

O transplante de células-tronco hematopoiéticas (TCTH) é a infusão de células responsáveis pela produção sanguínea para que se repliquem no receptor e produzam células sanguíneas normais.^(1)^ O termo “transplante de medula óssea” (TMO) vem sendo usado há muitos anos, pois a medula foi a primeira fonte de células-tronco sanguíneas utilizada para o transplante.^(1)^

Os TCTH são realizados com sucesso desde a década de 1960: três bebês com imunodeficiência congênita receberam a medula de seus irmãos e corrigiram sua doença, tornando-se adultos saudáveis.^(1)^ Foi uma longa jornada até atingir o marco de 1 milhão de transplantes realizados em todo o mundo no ano de 2014.^(2)^ Atualmente, existem mais de 1.500 centros que realizam TCTH em pelo menos 75 países. Grande parte desses centros relata seus resultados ao *Center for International Blood and Marrow Transplant Research* (CIBMTR) ou à *European Bone Marrow Transplantation* (EBMT), e, em seus *sites*, é possível encontrar um resumo de dados estatísticos, protocolos, orientações de tratamento e dezenas de aulas dos cursos e congressos.^(3–5)^

No Brasil, o Serviço de Transplante de Medula Óssea (STMO) do Hospital de Clínicas da Universidade Federal do Paraná foi o primeiro a iniciar suas atividades, em 1979. Em 2016, 2.186 TCTH foram relatados ao Registro Brasileiro de Transplante (RBT) da Associação Brasileira de Transplante de Órgãos (ABTO). A taxa global de transplantes por milhão de habitantes em nosso país relatada à ABTO é de 10,7, enquanto países como Inglaterra, Espanha, Estados Unidos e Alemanha realizam mais de 50 transplantes por milhão de habitantes.^(2,6,7)^

Nos transplantes de órgãos sólidos, é muito importante a compatibilidade da tipagem sanguínea (A, B, AB, O), mas, nos TCTHs, o receptor passa a produzir hemácias com a tipagem de seu doador. Nesse caso, o fator mais importante é a compatibilidade da tipagem do sistema de antígenos leucocitários humanos (HLA - *human leukocyte antigen*).^(7)^

O HLA é totalmente codificado no cromossoma 6, e todos juntos são herdados dos pais, em bloco, incluindo os alelos A, B, C (classe I), DR, DP e DQ (classe II). Dependendo do número de alelos tipados, a compatibilidade é de seis antígenos (A, B e DR), oito (A, B, C e DR), dez (A, B, C, DR e DQ) ou 12.^(7)^ Assim, irmãos têm chance de terem herdado tipagem HLA idêntica ou distinta em 25% dos casos, e metade dos irmãos tem chance de ter herdado um cromossoma igual e um diferente, sendo chamados de haploidênticos. Assim, somos sempre haploidênticos a nossos pais e filhos.^(7)^

Existem vários tipos de TCTH, que são classificados de acordo com quem doa as células, com a fonte das células, o grau de compatibilidade entre doador e receptor e o tipo de condicionamento, conforme tabela 1.

**Tabela 1:** Tipos de transplante de células-tronco hematopoiéticas

Item de classificação	Tipo de transplante
Doador das células hematopoiéticas	Autólogo ou autogênico: do próprio paciente
	Singênico: o doador é um irmão gêmeo univitelino
	Alogênico: o doador é um indivíduo geneticamente distinto, em geral com tipagem HLA compatível
Grau de parentesco entre doador e receptor nos transplantes alogênicos	Aparentado: doadores familiares com laços de sangue
	Não aparentado (ou não relacionado): doadores adultos (Registro de Doadores Voluntários de Medula Óssea) ou unidades de sangue de cordão umbilical (bancos de sangue de cordão umbilical/placentário)
Regime de condicionamento para o transplante	Mieloablativo: utiliza altas doses de quimioterapia, associadas ou não à radioterapia. Sem a infusão de células-tronco hematopoiéticas, esse tratamento levaria à morte, por dano irreversível à medula óssea
	Transplante de toxicidade reduzida: transplante mieloablativo, mas com redução na dosagem de um ou mais medicamentos, para que possam ser mais bem tolerados
	Transplante de intensidade reduzida (não mieloablativo, inicialmente chamado de minitransplante): utiliza esquemas que não destroem totalmente a medula do paciente. Baseia-se na imunossupressão, para impedir que o receptor rejeite as células do doador. Há troca gradual das células do receptor para as do doador. Inicialmente, a medula do paciente e a do doador produzem juntas as células sanguíneas (quimeirismo misto). Com o passar dos dias, a medula do doador pode conseguir ocupar todo o espaço medular e passar a ser a única responsável pela produção do sangue (quimeirismo completo, ou seja, 100% das células da medula óssea são provenientes do doador)
Origem das células-tronco hematopoiéticas em transplantes autólogos ou alogênicos	Medula óssea: fonte mais comum em transplantes pediátricos alogênicos. A medula é coletada no centro cirúrgico diretamente das cristas ilíacas, sob anestesia geral
	Sangue periférico: as células são coletadas pelo procedimento de leucoaférese, após estímulo com fatores de crescimento (G-CSF). Esse processo chama-se “mobilização”. Em transplantes autólogos, antes do início do G-CSF, pode ser também ser utilizada quimioterapia, como altas doses de ciclofosfamida. As células-tronco do sangue periférico são a fonte mais comum em transplantes autólogos
	Sangue de cordão umbilical/placenta: coletado e criopreservado logo após o nascimento da criança, geralmente utilizado apenas em transplantes alogênicos, principalmente não aparentados

HLA: antígeno leucocitário humano; G-CSF: fator estimulador de colônias granulocitárias.

Antes do início do condicionamento, os pacientes precisam de um cateter venoso central parcialmente implantável (permanente, tipo Hickman, Permcath ou temporário), usado para coleta seriada de exames e para que sejam administrados quimioterapia, hidratação, hemoderivados e outras medicações. Os dias que antecedem a infusão das células-tronco são contados como negativos e, após a infusão, como positivos.

O dia anterior ao transplante (D-1), em geral, é o dia de “descanso”, quando se aguardam a metabolização e a excreção da quimioterapia, para que as células não sejam afetadas no momento de sua infusão. Entretanto, é importante aguardar, no mínimo, duas meias-vidas terminais do regime quimioterápico até a infusão, o que significa menos de 2 horas para medicamentos como melfalano ou até 4 dias com a carboplatina.^(1)^

Os quimioterápicos mais comumente utilizados no condicionamento são ciclofosfamida, bussulfano, citarabina, etoposido, melfalano, carmustina, tiotepa, entre outros. Cada quimioterápico tem um padrão de toxicidade distinto. As medicações cuja toxicidade gastrintestinal é maior são bussulfano, etoposido, melfalano e tiotepa, além da irradiação corporal total (TBI - *total body irradiation*).^(1)^

O dia da infusão, ou o dia do transplante, é definido como dia zero (D0).^(1)^

## TRANSPLANTE DE CÉLULAS-TRONCO HEMATOPOIÉTICAS AUTÓLOGO

### Procedimento de coleta de células

Nos transplantes autólogos, as células-tronco do próprio indivíduo são geralmente coletadas do sangue periférico, criopreservadas e reinfundidas depois de o paciente receber altas doses de quimioterapia (condicionamento ou regime preparatório). Assim, é possível lançar mão de doses mieloablativas de quimioterapia e proporcionar a recuperação hematológica.

Para a mobilização, o paciente é submetido a algumas etapas. Inicia por uma quimioterapia (opcional, geralmente com 1g/m^2^/dia a 2g/m^2^/dia de ciclofosfamida).

Em seguida é iniciado o uso diário de fator estimulador de colônias granulocitárias (G-CSF), em uma ou duas aplicações intravenosa (IV) ou subcutânea, até que as células-tronco tenham sido coletadas por leucoaférese ou que se considere que houve falha na mobilização. Durante todo o período, monitora-se o número de células-tronco CD34+ no sangue periférico por citometria de fluxo, e, quando este atinge o nível adequado (geralmente superior a 10 células/mcL a 20 células/mcL), geralmente é o momento ideal para a coleta, realizada por meio do cateter venoso central de duplo lúmen próprio para hemodiálise – temporário (por exemplo: Shilley) ou permanente (por exemplo: Permcath) –, e o início das leucoaféreses.

Na leucoaférese, o sangue do paciente é continuamente processado em um equipamento de separação celular. Os leucócitos são lentamente coletados em uma bolsa própria, e hemácias e plasma são continuamente devolvidos pela outra via do cateter. A leucoaférese dura algumas horas e pode ser repetido no dia seguinte, caso não sejam coletadas células suficientes para o transplante – em geral 3 a 5 milhões de células CD34/kg de peso por transplante. Alguns esquemas pediátricos de tratamento de tumores do sistema nervoso central, neuroblastoma e tumores de células germinativas preveem até três ciclos sequenciais de altas doses de quimioterapia, podendo ser necessário um grande número de células.

As células coletadas são congeladas (criopreservadas) em laboratórios especializados utilizando soluções que contêm dimetilsulfóxido (DMSO), um crioprotetor que minimiza o dano causado pelos processos de congelamento e degelo. As células-tronco podem permanecer em *freezer* em -80°C por vários meses e em tanques de nitrogênio por décadas, até que seu uso seja necessário.

### Durante o transplante

Para os transplantes, as crianças necessitam de um acesso parcialmente implantável (tipo Hickman, Permcath ou outros cateteres) permanente ou de um cateter parcialmente implantável temporário, para o recebimento das células e da quimioterapia, que antecede a infusão.

Nos dias negativos (D-6, D-5, D-4, D-3 e D-2), é administrado o regime de condicionamento, ou regime preparatório, com altas doses de quimioterapia, com o intuito de destruir as células tumorais resistentes aos esquemas e às doses habituais de quimioterapia. Existe, a seguir, um “descanso” da quimioterapia, geralmente no D-1.

No D0, as células autólogas são descongeladas e infundidas, por meio do cateter venoso central. A infusão pode ter vários efeitos adversos, devido à presença do crioprotetor, sendo os mais frequentes exalação de odor peculiar pela excreção respiratória do DMSO (várias descrições como creme de milho, alho etc.), bradicardia, alterações pressóricas, náuseas e vômitos.

A partir do D+1, o dia seguinte à infusão, os pacientes podem iniciar (ou não) G-CSF diário, além das medicações para profilaxia anti-infecciosa (vírus, fungos e bactérias), que podem variar conforme protocolos institucionais. Nos dias que seguem a infusão, os pacientes podem ainda manifestar muita toxicidade gastrintestinal da quimioterapia, com mucosite oral, náuseas, vômitos e diarreia, que podem causar muito desconforto e dor grave, demandando analgésicos potentes.

Devido à supressão medular, pacientes têm anemia e plaquetopenia, que necessitam de transfusões frequentes e neutropenia, com grande suscetibilidade às infecções. É comum o surgimento de febre que, mesmo sem foco definido, precisa ser tratada com antibióticos de amplo espectro.

A “pega da medula” é o primeiro de 3 dias consecutivos, após o nadir da quimioterapia, em que o paciente apresenta 500 neutrófilos/mm^3^ ou mais no hemograma. A definição de pega de plaquetas, em geral, é o primeiro dia com mais de 20 mil plaquetas/mm^3^ após 7 ou mais dias sem transfusão.

A complexidade e as complicações dos transplantes são menores no do tipo autólogo, quando comparado ao alogênico. Do ponto de vista nutricional, entretanto, alguns pacientes já vêm recebendo quimioterapia há vários meses quando chegam ao TMO e, assim, podem ter *deficit* nutricionais importantes. Além disso, algumas crianças precisam receber radioterapia após o transplante. Quando o campo da radioterapia envolve a região do esôfago ou abdome, ela pode levar a lesão da mucosa, náuseas, vômito e diarreia, retardando ainda mais a recuperação nutricional.

Os transplantes autólogos são a modalidade de transplante mais frequente em adultos, sendo utilizados no tratamento do mieloma múltiplo e linfomas.^(6)^ Na pediatria, há evidências de que TMO autólogo possa proporcionar maior sobrevida no tratamento de linfoma de Hodgkin, linfomas não Hodgkin a partir da segunda remissão e tumores sólidos quimiossensíveis, como tumores de células germinativas em segunda remissão ou neuroblastoma de alto risco.^(6)^ Nos bebês com tumores do sistema nervoso central, o TCTH autólogo pode ser utilizado para substituir ou postergar a radioterapia, já que seus efeitos deletérios em longo prazo sobre o cérebro em desenvolvimento seriam devastadores.^(6)^ Alguns estudos indicam o benefício do TMO no tratamento de sarcoma de Ewing metastático ou em segunda remissão, tumores primitivos do sistema nervoso central disseminados ou em segunda remissão, retinoblastoma extraocular e tumor de Wilms após a segunda remissão.

Os transplantes autólogos podem ser usados ainda para erradicar células autorreativas, no tratamento de doenças autoimunes graves, e, em países que dispõem de infraestrutura apropriada, as células-tronco autólogas podem ser usadas para correção genética em laboratório, na chamada “terapia gênica”.

### Transplante alogênico

Nos transplantes alogênicos, os esquemas clássicos mieloablativos de condicionamento em pediatria são ciclofosfamida 120mg/kg a 200mg/kg, com TBI de 1.200 cGy para condicionamento de leucemia linfoide aguda, e ciclofosfamida 200mg/kg, com bussulfano para leucemias mieloides. Para doenças não malignas, são frequentemente utilizados bussulfano e fludarabina ou melfalano.^(5)^

Além da quimioterapia, o regime preparatório inclui medicações utilizadas para diminuir a chance de que o receptor destrua as células do doador (rejeição), como a globulina antitimocítica, principalmente nos transplantes não aparentados, e a ciclosporina, e a chance de as células do doador atacarem o receptor, ocasionando a doença do enxerto contra o hospedeiro (DECH).^(3,5)^

As células-tronco do doador são geralmente coletadas e infundidas no próprio D0. No tratamento de doenças malignas, podem ser utilizadas células coletadas do sangue periférico, com uso exclusivo de G-CSF, sem quimioterapia, se for factível a leucoaférese por acesso venoso periférico, sem a necessidade de inserção de cateter venoso central.

A medula óssea é a fonte mais comum para o TCTH alogênico.^(3)^ Para coleta de medula óssea, o doador é levado ao centro cirúrgico e, sob anestesia geral e/ou bloqueio espinal (peridural), as células são coletadas das cristas ilíacas posteriores com agulhas e seringas. São removidos ao redor de 15mL de medula/kg de peso do receptor e no máximo 20mL/kg de peso do doador. A coleta demora em torno de 1 a 2 horas e, em geral, o doador tem alta no mesmo dia ou no dia seguinte. Em transplantes não aparentados, pode ser necessário realizar a coleta das células até 2 dias antes da infusão, o que geralmente mantém excelente viabilidade.

Outras medicações usadas depois do transplante para prevenir a DECH são a própria ciclosporina, o tacrolimus (inibidores de calcineurina), o metotrexato (D+1, D+3, D+6 e, às vezes, também no D+11) e o micofenolato mofetil. Recentemente, têm sido usadas altas doses de ciclofosfamida, infundida no D+3 e no D+4, principalmente nos transplantes haploidênticos.

Os transplantes alogênicos são utilizados principalmente no tratamento de leucemias, mielodisplasia e falências medulares, entre outros.^(6)^ As células do doador são infundidas após se administrar o regime de condicionamento, que inclui altas doses de quimioterapia ou TBI.

Embora as células do doador possam reconhecer o receptor como estranho e atacá-lo (DECH), essas mesmas células podem reconhecer e destruir células tumorais e diminuir a chance de recidiva da doença maligna (efeito do enxerto contra o tumor ou *graft-versus-leukemia* – GVL).

A toxicidade máxima associada ao TCTH é geralmente expressa como a mortalidade relacionada ao transplante (MRT), ou seja, mortalidade nos primeiros 100 dias após a infusão das células-tronco por quaisquer causas, exceto recidiva da doença de base. A MRT aumenta muito se a doença estiver em fases avançadas. Resultados publicados pelo CIBMTR demonstram 2% a 10% de MRT nos TCTH autólogos, 7% a 22% nos alogênicos aparentados e 10% a 25% nos alogênicos não aparentados.^(4)^

As toxicidades apresentadas podem ser agrupadas como agudas e frequentes e de menor frequência e maior gravidade, conforme a tabela 2.

**Tabela 2:** Toxicidades relacionadas ao transplante de células-tronco hematopoiéticas alogênico

Agudas e frequentes	Menor frequência e maior gravidade
Mucosite oral e em todo trato gastrintestinal	Síndrome de obstrução sinusoidal (ou doença venoclusiva) do fígado
Náuseas, vômitos e diarreia de intensidade variável	Cistite hemorrágica por toxicidade das drogas ou por infecções virais
Febre, em geral por infecções bacterianas, principalmente durante a neutropenia, antes da pega da medula	Infecções fúngicas invasivas
	Infecções virais
Insuficiência renal leve a moderada devido à irradiação e a medicações	Rejeição do enxerto (falha de pega)
Hipertensão arterial transitória devido ao uso de ciclosporina e/ou corticoides	Pneumonite intersticial idiopática ou infecciosa, principalmente por citomegalovírus e vírus respiratórios.
DECH aguda	Outras infecções virais
	Toxicidade cardíaca
	Toxicidade ao sistema nervoso central – convulsões e acidente vascular cerebral
	Microangiopatia trombótica

Fonte: Pasquini M, Wang Z, Horowitz MM, Gale RP. 2013 report from the Center for International Blood and Marrow Transplant Research (CIBMTR): current uses and outcomes of hematopoietic cell transplants for blood and bone marrow disorders. Clin Transpl. 2013:187-97.^(4)^

DECH: doença do enxerto contra o hospedeiro.

As complicações infecciosas costumam seguir um padrão mais ou menos constante. Nos primeiros 30 dias, o paciente com neutropenia e mucosite está predisposto a infecções pelo vírus do herpes simples, vírus respiratórios, bactérias *Gram*-positivas e negativas e *Candida sp.* A partir daí, a DECH passa ser o principal fator predisponente às infecções. Entre o primeiro e segundo mês, permanecem como principais fatores os vírus respiratórios, o adenovírus, citomegalovírus, além de infecções por fungos filamentosos.

Após a pega, persiste a imunodeficiência grave pelo período de vários meses, principalmente após transplantes alogênicos e autólogos com uso de rituximabe no tratamento de linfomas. São frequentes infecções por citomegalovírus, adenovírus, herpes-zóster, *Pneumocystis carinii*, toxoplasmose e, em pacientes que desenvolvem DECH crônica (DECHc) ou nos esplenectomizados, há risco de infecções fulminantes por bactérias encapsuladas.

Pacientes esplenectomizados, com radioterapia esplênica, ou que tiveram DECHc devem ser extensivamente educados para o alto risco de sepse fulminante, bem como receber profilaxia contínua contra bactérias encapsuladas e estar orientados a procurar o serviço de referência em caso de febre, pois precisam coletar hemocultura e iniciar imediatamente o uso de antibióticos apropriados, como amoxacilina-clavulonato ou ceftriaxona.

Em razão dos riscos em curto e longo prazos associados aos transplantes, sua indicação se restringe ao tratamento de doenças nas quais a sobrevida com o TCTH é superior à sobrevida com o tratamento convencional (por exemplo, a quimioterapia), ou quando o transplante pode promover uma melhora significativa da qualidade de vida, como eliminar a necessidade de hipertransfusão e as complicações no longo prazo em pacientes com hemoglobinopatias.

As indicações de TCTH são objeto de contínua reavaliação. Idealmente, as indicações de transplantes deveriam partir de resultados de estudos clínicos randomizados, mas isso nem sempre é possível. Apenas conhecendo os resultados com tratamento convencional e com TCTH no nosso meio seria possível fazer indicações precisas.

À medida que ocorrem avanços terapêuticos, algumas doenças para as quais o TCTH era indicado podem passar a ter outra abordagem mais adequada, como ocorreu com o uso do mesilato de imatinib (Glivec^®^) no tratamento da leucemia mieloide crônica. Entretanto, doenças para as quais não se concebia a utilização do TCTH passaram a ser tratadas com esse procedimento, como anemia falciforme e doenças de depósito.

Em pediatria, as indicações mais comuns de transplante alogênico são leucemia linfoide aguda em segunda remissão medular, leucemia mieloide aguda, síndromes mielodisplásicas, síndromes de falência medular, como aplasia grave de medula óssea e anemia de Fanconi, imunodeficiências, alguns casos de hemoglobinopatias e doenças metabólicas hereditárias.

No Brasil, as doenças que podem ser tratadas com transplante pelo Sistema Único de Saúde (SUS) são listadas na portaria 940, de 21 de dezembro de 2006 (Tabela 3), não modificadas até a presente data em 2020.^(8)^

**Tabela 3:** Critérios de indicação de transplante de células-tronco hematopoéticas em doenças pediátricas

Diagnóstico		CID	Transplantes autorizados	
Aplasia pura adquirida crônica da série vermelha		D60.0	Alogênico aparentado	MO ou SCU
			Alogênico não aparentado	MO ou SCU
Anemia aplástica		D61.0	Alogênico aparentado	MO ou SCU
		61.3	Alogênico não aparentado	MO ou SCU
Imunodeficiências		D80.0 - 83.2	Alogênico aparentado	MO ou SCU
			Alogênico não aparentado	MO, SP ou SCU
Talassemia beta – *major*		D56.1	Alogênico aparentado	MO, SP ou SCU
Leucemia linfoide aguda		C91.0	Alogênico aparentado	MO, SP ou SCU
			Alogênico não aparentado	MO, SP ou SCU
Linfoma linfoblástico		C83.5	Alogênico aparentado	MO, SP ou SCU
			Alogênico não aparentado	MO, SP ou SCU
Leucemia mieloide aguda		C92.0	Alogênico aparentado	MO, SP ou SCU
			Alogênico não aparentado	MO, SP ou SCU
			Autólogo	MO ou SP
Leucemia mieloide crônica		C92.1	Alogênico aparentado	MO ou SP
			Alogênico não aparentado	MO ou SP
Linfoma não Hodgkin		C83.2 - 83.7	Autólogo	MO ou SP
Linfoma de Hodgkin		C81.0 - 81.3	Autólogo	MO ou SP
			Alogênico aparentado	MO, SP ou SCU
Síndrome mielodisplásica		D46.2 - 42.3	Alogênico aparentado	MO, SP ou SCU
Tumor células germinativas				
	Mediastino	C38.1 - 38.3	Autólogo	MO ou SP
	Retroperitôneo	C48.8		
	Ovário	C56.0		
	Testículo	C62.0 - 62.1		

Fonte: Brasil. Ministério da Saúde. Secretaria de Atenção à Saúde. Portaria SAS nº. 940 de 21 de dezembro de 2006. Altera o atributo NOME dos procedimentos referentes a TCTH. Brasília (DF): Diário Oficial do Brasil; 2006 Dez de 21 [citado 2020 Mar 10]. Disponível em: http://www.normasbrasil.com.br/norma/portaria-940-2006_197770.html.^(8)^

CID: Classificação Internacional de Doenças e Problemas Relacionados à Saúde; MO: medula óssea; SCU: sangue de cordão umbilical; SP: sangue periférico.

A sobrevida após o TMO para o tratamento de câncer depende de diversos fatores, como doença de base, tratamento prévio, tempo de evolução, faixa etária e número de recidivas. Em geral, 50% a 60% das crianças são curadas, dependendo da fase da doença em que o transplante é realizado.

Atualmente, o maior desafio é aumentar a chance de cura das crianças. Para isso, é necessário reduzir a toxicidade dos transplantes e melhorar seus resultados. Além disso, é fundamental conhecer a realidade do tratamento de nossos pacientes com e sem transplante, permitindo uma melhor seleção dos candidatos ao TCTH.

### Importância do estado nutricional no transplante de células-tronco hematopoiéticas

É uma situação comum os pacientes serem internados em bom estado nutricional e apresentarem comprometimento importante no decorrer do TCTH.^(9)^ Tanto a toxicidade do tratamento quanto suas potenciais complicações levam à redução da aceitação alimentar e/ou ao comprometimento da absorção adequada de nutrientes. Tal situação é ainda mais alarmante na criança e no adolescente, uma vez que estão em processo de crescimento e desenvolvimento. Pacientes que fizeram uso de TBI têm maior comprometimento estatural comparado com regimes de condicionamento baseados somente em quimioterapia.^(10)^

A relação entre o estado nutricional e os desfechos do transplante tem sido explorada na última década com publicações inicialmente em pacientes adultos relatando, por exemplo, menor sobrevida global e maior toxicidade, risco de DECH agudo, tempo de internação e tempo para enxertia plaquetária e neutrofílica em pacientes desnutridos ou que tiveram perda de peso grave durante o transplante.^(11–13)^ Embora não seja possível transpor todos esses resultados para a pediatria, imagina-se que os efeitos do comprometimento do estado nutricional tenham ainda maior impacto em crianças e adolescentes, não só pelas potenciais sequelas ao crescimento e ao desenvolvimento adequados, mas também pela menor massa corporal e o que sua perda representa nessa faixa etária.

A presença de desnutrição no TCTH pediátrico está associada a uma menor sobrevida global e à maior mortalidade sem recaída, bem como a maior ao risco de recaída da doença oncológica.^(9,14)^ Além disso, foram observados maior presença de fraqueza pós-transplante e maior risco de DECH crônico.^(9,15)^ Pacientes nessa condição nutricional parecem também demorar mais a recuperar o peso e a massa magra perdidos durante o TCTH.^(16)^

Da mesma maneira, a perda de peso durante o transplante está associada a riscos importantes ao paciente pediátrico, como maior prevalência de DECH em múltiplos órgãos e presença de menor ângulo de fase padronizado, medido por bioimpedância.^(16,17)^ Esse último parâmetro está relacionado a uma maior mortalidade e ao risco aumentado de desenvolvimento de DECH em sua forma crônica.

Embora por ora exista menor número de publicações, recentemente crianças e adolescentes com sobrepeso e obesidade vêm sendo estudados. Da mesma maneira que os desnutridos, pacientes acima do peso também apresentam maior prevalência de fraqueza pós-transplante, menor sobrevida global e potencialmente um risco aumentado para DECHc.^(15,18,19)^

A má nutrição como um todo, representando tanto a desnutrição quanto o sobrepeso/obesidade e, ainda, a perda severa de peso, pode gerar consequências dramáticas à criança e ao adolescente que passa por TCTH. As atuais evidências publicadas levam a crer que o seguimento nutricional desses pacientes é essencial ao sucesso da terapia proposta.

### Avaliação nutricional

Vários são os fatores que determinam o sucesso do TCTH, a curto e longo prazo. Dentre eles, destacam-se o diagnóstico e o estágio da doença, o tipo de transplante realizado, a presença de incompatibilidade com o doador, o regime de condicionamento, a fonte de células, a idade, os tratamentos prévios e o estado nutricional.^(20)^ Em relação a esse último, consideram-se em risco nutricional todos os pacientes candidatos ao TCTH. Com o objetivo de diminuir o impacto negativo da doença e do tratamento utilizado, recomenda-se adequada avaliação nutricional tanto no pré como no pós-transplante.^(21)^ Assim, fazem parte dessa avaliação: a anamnese nutricional, com informações referentes à história clínica, nutricional e socioeconômica; a descrição dos dados antropométricos; um completo exame físico; a investigação referente ao padrão da ingestão alimentar e a realização de exames laboratoriais, com informações referentes, principalmente, ao hemograma e à bioquímica.

### Antropometria

A antropometria tem sido largamente utilizada para a avaliação do estado nutricional na população pediátrica, por ser um método prático, não invasivo e de baixo custo. As medidas comumente avaliadas são peso, estatura e índice de massa corporal (IMC).^(22)^

A classificação utilizada são os parâmetros recomendados pela Organização Mundial da Saúde (OMS) de 2006 e 2007. Os dados de peso e estatura são analisados conforme idade e sexo e classificados pelos índices do escore-z: peso/idade (P/I), estatura ou comprimento/idade (E/I), peso/estatura (P/E) e IMC/idade (IMC/I). Além do acompanhamento individual de cada parâmetro, conforme tabelas 4 e 5, a classificação final acontece de acordo com a faixa etária: menores de 2 anos, escore-z P/E e, para maiores de 2 anos, IMC/I. O paciente deve ter sua evolução acompanhada também pelos gráficos de crescimento de acordo com parâmetro, sexo e idade.^(23–26)^

**Tabela 4:** Índice antropométrico determinante do estado nutricional por faixa etária de zero a 10 anos

Valores críticos	Índices antropométricos						
	Crianças de 0 a 5 anos incompletos				Crianças de 5 a 10 anos incompletos		
	P/I	P/E	IMC/I	E/I	P/I	IMC/I	E/I
< Escore-z -3	Muito baixo peso para a idade	Magreza acentuada	Magreza acentuada	Muito baixa estatura para a idade	Muito baixo peso para a idade	Magreza acentuada	Muito baixa estatura para a idade
> Escore-z -3 e < escore-z -2	Baixo peso para a idade	Magreza	Magreza	Baixa estatura para idade	Baixo peso para a idade	Magreza	Baixa estatura para idade
> Escore-z -2 e < escore-z -1	Peso adequado para idade	Eutrofia	Eutrofia	Estatura adequada para idade	Peso adequado para idade	Eutrofia	Estatura adequada para idade
> Escore-z -1 e < escore-z +1							
> Escore-z +1 e < escore-z +2		Risco de sobrepeso	Risco de sobrepeso			Sobrepeso	
> Escore-z + 2 e < escore-z +3	Peso elevado para idade	Sobrepeso	Sobrepeso		Peso elevado para idade	Obesidade	
> Escore-z +3		Obesidade	Obesidade			Obesidade grave	

Fonte: Sociedade Brasileira de Pediatria (SBP). Avaliação nutricional da criança e do adolescente: manual de orientação. Departamento de Nutrologia. Rio de Janeiro: SBP; 2009. Capítulo 5. Avaliação da composição corporal. p.46-51;^(23)^ Silva AP, Nascimento AG. Zamberlan P. Manual de dietas e condutas nutricionais em pediatria. São Paulo: Atheneu; 2014. p. 21-6.^(24)^

P/I: peso/idade; P/E: peso/estatura; IMC/I: índice de massa corporal/idade; E/I: estatura/idade.

**Tabela 5:** Índice antropométrico determinante do estado nutricional por faixa etária de 10 a 19 anos

Valores críticos	Índices antropométricos para adolescentes	
	IMC/I	E/I
< Escore-z -3	Magreza acentuada	Muito baixa estatura para a idade
> Escore-z -3 e <escore-z -2	Magreza	Baixa estatura para idade
> Escore-z -2 e > escore-z -1	Eutrofia	Estatura adequada para idade
> Escore-z -1 e < escore-z +1		
> Escore-z +1 e < escore-z +2	Sobrepeso	
> Escore-z +2 e < escore-z +3	Obesidade	
> Escore-z +3	Obesidade grave	

Fonte: Sociedade Brasileira de Pediatria (SBP). Avaliação nutricional da criança e do adolescente: manual de orientação. Departamento de Nutrologia. Rio de Janeiro: SBP; 2009. Capítulo 5. Avaliação da composição corporal. p.46-51;^(23)^ Silva AP, Nascimento AG. Zamberlan P. Manual de dietas e condutas nutricionais em pediatria. São Paulo: Atheneu; 2014. p. 21-6.^(24)^

IMC/I: índice de massa corporal /idade; E/I: estatura ou comprimento.

### Composição corporal

A avaliação da composição corporal como uma das ferramentas da terapia nutricional tem sido cada vez mais valorizada nos últimos anos, uma vez que apenas o cálculo do peso ou do IMC para a idade não são suficientes para avaliar as massas gorda e magra em crianças e adolescentes com doenças crônicas.^(27–31)^ Além disso, esses indicadores são pouco sensíveis à variação do estado nutricional, uma vez que o peso corpóreo é enviesado por alterações hídricas importantes decorrentes do TCTH. Existem vários métodos para a avaliação de composição corporal, como circunferências e dobras cutâneas, ressonância magnética, tomografia computadorizada, densitometria corporal, ultrassonografia, bioimpedanciometria, potássio corporal total e pletismografia a ar.^(29–31)^

Na pediatria, associado ao TCTH alogênico, muitos trabalhos utilizam a densitometria corporal para essa avaliação, que não é realizada na maioria dos serviços brasileiros, pelo alto custo e/ou ausência do método.^(31,32)^ Tanto a obesidade quanto a desnutrição são fatores de risco nesse procedimento, destacando a menor quantidade de massa muscular e a maior de gordura periférica e visceral.^(18,31–35)^

As dobras cutâneas tricipital (DCT) e subescapular são as mais utilizadas em pediatria por possuírem valores de referência populacionais para essa faixa etária.^(25,36,37)^ Quando tais valores encontram-se abaixo do percentil 5 ou acima do 90, há riscos à saúde. A medida de DCT constitui um meio conveniente para estabelecer indiretamente a massa de gordura corporal.^(38,39)^ As medidas circunferência de braço e área muscular do braço são usadas primariamente para se obter uma mensuração de quantidade e da taxa de variação de proteína muscular esquelética. Deve-se lembrar que, em condições de anasarca, a circunferência de braço e a DCT têm aplicabilidade limitada.

Apesar da escassez de publicações em crianças durante TCTH, atualmente alguns estudos apontam o uso das mensurações da DCT, da circunferência de braço e da área muscular do braço como os melhores indicadores da composição corporal de pacientes oncológicos pediátricos, devido à sua facilidade de aferição, ao baixo custo e por serem procedimentos não invasivos, além de terem boa correlação com outros parâmetros considerados padrão-ouro para composição corporal nessa população. Estudos têm recomendado a mensuração da antropometria do braço baseada na comparação com os índices de peso e medidas não referenciadas de desnutrição.^(22,39–43)^

A irradiação em altas doses no crânio usada na TBI pode causar alterações no hipotálamo, incluindo mudanças nas funções do hormônio do crescimento, tireoide e gonadal, além de alterações na sensibilidade a leptina, grelina e insulina.^(31)^ Tais variações estão associadas à obesidade, principalmente no pós-TCTH, incluindo aumento da gordura visceral e de infiltração gordurosa hepática.^(31,33)^ Pacientes que recebem TBI no condicionamento, quando comparados àqueles que recebem apenas quimioterapia ou a controles obesos, apresentam menos massa magra, com maior prevalência de perda desta e mais gordura visceral e intramuscular.^(33)^

Além da irradiação, a DECH, uma das mais comuns complicações do TCTH, correlaciona-se a alterações da composição corporal.^(31)^ Na DECH intestinal, é maior a perda de peso, com redução de massa muscular – na maioria dos casos sem aumento da gordura visceral.^(31)^ Já o uso de corticoide no tratamento da DECH contribui para o aumento da gordura visceral e periférica, além da redução da massa magra desses pacientes.^(18,32)^

Nos sobreviventes do TCTH, a maior propensão à obesidade e à redução da massa magra relaciona-se a um maior risco de doença cardiovascular, síndrome metabólica, *deficit* de crescimento e ósseo, levando a maior morbidade e mortalidade nessas crianças.^(31,32,34)^

O TCTH alogênico, a TBI, o uso de corticoide e o DECH são importantes fatores de risco para alterações de composição corporal nos sobreviventes, estando associados ao aumento de massa gorda e à perda de massa muscular.^(18,31,32,34–36)^ Pacientes submetidos a esses fatores devem ter sua composição corporal mais bem monitorada como medida terapêutica precoce, reduzindo o risco de morbimortalidade.^(31,32,35)^ O ângulo de fase medido pela bioimpedanciometria parece promissor como medida *proxy* de estado nutricional, podendo ser uma opção de acompanhamento desses pacientes. Em estudo brasileiro publicado em 2013, crianças que tinham menor ângulo de fase apresentaram perda de peso severa, maior incidência de DECH e menor sobrevida.^(9)^

Embora em outras áreas da nutrição a avaliação da composição corporal esteja nos protocolos institucionais, na maioria dos serviços brasileiros de TCTH pediátrico não há uniformidade. Recomenda-se que essa avaliação seja realizada em todas as etapas do TCTH, antes, durante e depois, a fim de melhorar a sobrevida e a qualidade de vida desses pacientes. Cada serviço deve escolher o método que tenha o melhor custo-benefício e praticidade dentre os vários validados.

### Parâmetros laboratoriais

Os parâmetros laboratoriais são importantes na avaliação do estado nutricional, fornecendo medidas claras de suas alterações e tendo como vantagem possibilitar o seguimento ao longo do tempo e permitir intervenções precoces.^(43)^ Além disso, podem auxiliar no monitoramento nutricional e na tomada de decisão de alguma terapia nutricional específica. Assim, é possível dividir os parâmetros laboratoriais em dois grandes grupos: estado nutricional e monitoramento da terapia nutricional.

#### Estado nutricional

Deve-se ter em mente que, embora os diferentes exames bioquímicos ofereçam informações importantes referentes ao estado nutricional, nenhum deles deve ser utilizado isoladamente, visto que podem ocorrer alterações durante a evolução clínica do paciente. Dentre as variáveis bioquímicas de interesse na nutrição, relacionadas ao estado nutricional do paciente submetido ao TCTH destacamos:

- Albumina: é considerada a principal proteína sintetizada pelo fígado e, consequentemente, a mais utilizada na avaliação do estado nutricional. Concentrações séricas de albumina menores do que 3g/dL sugerem desnutrição proteica. Essa diminuição correlaciona-se também com aumento da incidência de complicações clínicas, da morbidade e da mortalidade. No paciente grave, os valores de albumina possuem valor prognóstico, quando dosados no momento da internação. Contudo, os valores mostram-se pouco sensíveis às alterações nas complicações agudas, já que sua meia-vida é relativamente longa (± 20 dias), podendo transcorrer várias semanas para que ocorra uma resposta às variações na ingestão dietético-proteica.^(44–46)^ Durante o TCTH, recomenda-se dosagem semanal de albumina. Valores menores do que 2,5mg/dL, neste momento, podem estar associados à sobrecarga de volume intravascular, síndrome de extravasamento capilar sistêmico, diarreia ou doenças hepáticas.^(47)^
- Pré-albumina: no soro, a pré-albumina transporta cerca de 70% da tiroxina, além de ser o transportador da proteína ligadora do retinol (RBP), as quais encontram-se diminuídas na desnutrição energético-proteica, assim como nas infecções, na cirrose, nas hepatites, nas inflamações, no estresse e nas doenças crônicas. Como marcador de estresse metabólico, pode ajudar a determinar a necessidade de intervenção nutricional. Tanto a pré-albumina quanto a RBP apresentam meia-vida curta, sendo consideradas importantes na avaliação do estado proteico visceral. A RBP apresenta meia-vida de apenas algumas horas (±12 horas) e a pressão arterial (PA), de 2 dias. Devido à sua pequena meia-vida, são consideradas um dos parâmetros mais sensíveis às alterações nutricionais.^(48)^ Consideram-se alterados valores de <10mg/dL nas crianças.^(47)^
- Transferrina: beta-globulina sintetizada no fígado e responsável pelo transporte de alguns nutrientes, especialmente o ferro. Possui vida média de 4 a 8 dias, fazendo com que seus níveis se encontrem alterados bem antes da albumina, nos casos de depleção proteica, tornando-se ferramenta importante para o diagnóstico da desnutrição subclínica. Todavia, apresenta baixa sensibilidade e especificidade quando analisada de forma individual, estando seus níveis aumentados na anemia ferropriva e diminuídos nas doenças hepáticas, na sepse, em casos de má absorção e em alterações inflamatórias.^(47,48)^ No paciente candidato ao TCTH, as múltiplas transfusões sanguíneas fazem com que a transferrina deixe de ser um bom parâmetro para avaliação. Consideram-se normais valores entre 200 a 400mg/dL. Valores inferiores a 200mg/dL são considerados indicativos de deficiência proteica leve a moderada, enquanto níveis inferiores a 100mg/dL correspondem à deficiência grave. Deve-se evitar usar a transferrina para avaliação da depleção proteica em pacientes com anemia ferropriva, uma vez que, nesses casos, seu valor encontra-se aumentado, refletindo o aumento do transporte de ferro.^(49)^

Os valores de referência das proteínas hepáticas (albumina, pré-albumina e transferrina) estão indicados na tabela 6.

**Tabela 6:** Valores de referência das proteínas hepáticas

Proteína sérica	Valores de referência	Função
Albumina, g/dL	Normal: >3,5	Principal responsável pela pressão coloidosmótica
	Depleção leve: 3,0-3,5	
	Depleção moderada: 2,5-2,9	
	Depleção grave: <2,5	
Pré-albumina, mg/dL	Normal: 20	Transportar hormônios da tireoide (tiroxina)
	Depleção leve: 10-15	
	Depleção moderada: 5-10	
	Depleção grave: <5	
Transferrina, mg/dL	Normal: 200-400	Transporte de alguns nutrientes, especialmente o ferro
	Depleção leve: 150-120	
	Depleção moderada: 100-150	
	Depleção grave: <100	

Fonte: Pedrón Giner C, Cuervas-Mons Vendrell M, Galera Martínez R, Gómez López L, Gomis Muñoz P, Irastorza Terradillos I, Martínez Costa C, Moreno Villares JM, Pérez-Portabella Maristany C, Pozas Del Río MᵃT, Redecillas Ferreiro SE, Prieto Bozano G; Grupo de Estandarización de la Senpe S. Pediatric parenteral nutrition: clinical practice guidelines from the Spanish Society of Parenteral and Enteral Nutrition (SENPE), the Spanish Society of Pediatric Gastroenterology, Hepatology and Nutrition (SEGHNP) and the Spanish Society of Hospital Pharmacy (SEFH). Nutr Hosp. 2017;34(3):745-58;^(50)^ Bottoni A, Oliveira GP, Ferrini MT, Waitzberg DL. Avaliação nutricional: exames laboratoriais. In: Waitzberg DL. Nutrição oral, enteral e parenteral na prática clínica. 3a ed. São Paulo: Atheneu; 2001. v. 1. p. 279-94.^(51)^

Contudo, é importante ter em mente que essas proteínas apresentam função limitada no paciente grave, devido à presença da hemodiluição e à modificação da atividade hepática na produção de proteínas de fase aguda, resultando em diminuição das proteínas viscerais. Em decorrência dessas alterações, valores diminuídos nesses pacientes refletem mais a gravidade da doença do que o estado nutricional.^(52)^ Importante notar que alguns autores correlacionaram alterações da transferrina e da pré-albumina à DECH.^(53)^

- Índice creatinina e altura (ICA): parâmetro adequado para avaliar a massa muscular corpórea, baseando-se no fato de que 98% da creatinina está armazenada nos músculos.^(54)^ É utilizado para avaliar a massa muscular e, indiretamente, o balanço nitrogenado. Pacientes desnutridos apresentam degradação intensa do músculo esquelético, a qual pode ser estimada a partir da dosagem da creatinina urinária, metabólito derivado da hidrólise da creatinina, cuja síntese é constante. Avalia, assim, o catabolismo muscular.^(42)^ O ICA é calculado como uma percentagem do padrão, por meio da divisão da excreção da creatinina urinária, por um valor padrão para a altura do paciente. O ICA normal aproxima-se de 1. Valores menores que 60% do padrão detectam pacientes com risco aumentado para desnutrição.^(46)^ Esse exame apresenta algumas desvantagens, como ser fidedigno somente em pacientes com função renal normal, necessidade da coleta da urina de 24 horas, interferência da idade e do conteúdo proteico da dieta, entre outras.^(55)^
- Balanço nitrogenado: é um método útil para avaliar o estado proteico. Permite determinar o grau de equilíbrio entre a ingestão e a excreção urinária de nitrogênio. Quando a ingestão é suficiente para cobrir as perdas, obtém-se um balanço positivo. Se, ao contrário, as perdas superam os ganhos, verifica-se um balanço negativo. É um bom exame para estimar a ingestão e a degradação proteica, tornando-se, dessa forma, importante ferramenta no seguimento e na monitorização do tratamento dos pacientes transplantados.^(56,57)^ Alguns autores consideram que o balanço nitrogenado não deve ser usado como parâmetro de desnutrição nas fases iniciais do tratamento, pela impossibilidade de se conseguir um balanço positivo nessa fase, mesmo em pacientes com suporte nutricional adequado.^(44)^
- Colesterol: a hipocolesterolemia (abaixo de 160mg/dL) tem sido associada a quadros de desnutrição e, consequentemente, ao aumento da mortalidade em pacientes críticos. Porém, níveis séricos baixos de colesterol também são observados na insuficiência renal, hepática e na má absorção. Sabidamente, níveis aumentados de colesterol são fator de risco para doença coronariana.^(44,57)^

### Monitoramento da terapia nutricional

Alguns exames rotineiramente colhidos de pacientes em TCTH auxiliam a nutrição na tomada de decisão sobre a melhor terapia nutricional. Apesar desses parâmetros apresentarem alterações devido à adaptação da medula, eles são utilizados de maneira complementar na elaboração da conduta nutricional. A aplicabilidade de seu conhecimento durante o TCTH se encontra na tabela 7.^(20,22)^

**Tabela 7:** Parâmetros laboratoriais e aplicação na terapia nutricional no período do transplante de células-tronco hematopoiéticas

Parâmetro laboratorial		Aplicação no TCTH
Hemograma completo	Hemoglobina	Importante na avaliação de anemia
		Apresenta-se alterada devido ao TCTH
		Não é um bom parâmetro bioquímico para crianças no transplante, se avaliada isoladamente
		Deve ser acompanhada para otimizar oferta de alimentos proteicos
	Leucócitos	Células de defesa do organismo
		Alterados até recuperação total da medula
		Indicadores de introdução de restrição dietética para alimentos potencialmente contaminantes (por exemplo: dieta sem crus) com valores abaixo de 1.500/mm³
	Neutrófilos	Células de defesa primária, principalmente em infecções bacterianas
		Apresentam-se alterados até recuperação total da medula ou devido ao uso de medicação
		Indicadores de introdução de restrição dietética para alimentos potencialmente contaminantes (exemplo: dieta sem crus) com valores abaixo de 500/mm³
	Plaquetas	Participa do processo de coagulação sanguínea
		Apresenta-se alterada até recuperação total da medula
		Quando está abaixo do valor de referência, deve-se ter cautela na indicação de passagem de sonda, pelo risco de sangramento, e também na avaliação da composição corporal
Função hepática	Transaminases	Parâmetros de teste de função hepática
		Geralmente alteradas durante o TCTH
		Acompanhar variações e, se necessário, controlar o consumo de alimentos e preparações ricas em gordura
	Bilirrubinas	Avaliam alterações hepáticas
		Se alteradas para valores superiores, podem identificar dano hepatocelular
		Acompanhar variações e, se necessário, controlar o consumo de alimentos e preparações ricas em gordura
Função renal	Creatinina	Indicadora de alteração de função renal
		Pode estar aumentada em situações de privação de hidratação prolongada
		Acompanhar exames e otimizar oferta hídrica
	Ureia	Pode indicar estado de hidratação
		Aumentada em casos de desidratação ou catabolismo proteico excessivo e diminuído em hiper-hidratação ou desnutrição
		Acompanhar exames e oferta hídrica
	Sódio	Se aumentado, indica baixa ingestão hídrica ou desidratação e, se diminuído, pode indicar edema
		Acompanhar exames hidratação e realizar avaliação antropométrica
	Potássio	Auxilia na avaliação da função renal
		Valores alterados podem otimizar ou restringir o consumo de alimentos ricos em potássio
Perfil lipídico	Colesterol total	Pode ser alterado ao uso de medicações ou alterações de consumo alimentar
		Acompanhar exames e adaptar dieta
	Triglicérides	Podem sofrer mudança com alteração de hábitos alimentares ou uso de medicações. Em uso de parenteral, pode ser utilizado para avaliar oferta de lipídios, para avaliar o início e a progressão da taxa de infusão
Glicemia		Pode sofrer alteração ao uso de corticosteroides, podendo ocasionar hiperglicemia
		Pacientes em longos períodos sem se alimentar podem apresentar hipoglicemia
		Realizar acompanhamento longitudinal e restrição de açúcar simples da dieta

TCTH: transplante de células-tronco hematopoiéticas.

A avaliação do estado nutricional utilizando parâmetros bioquímicos pode diagnosticar precocemente alterações nutricionais, antes mesmo de alterações antropométricas ou do surgimento de sinais e sintomas clínicos, constituindo importante ferramenta no acompanhamento desses pacientes, em conjunto com outros parâmetros de avaliação.^(52)^

### Exame físico

A realização do exame físico em pediatria é um indicador de diversas doenças pediátricas. Mesmo sendo uma avaliação com possíveis vieses devido à toxicidade ocasionada pelo TCTH, a realização do exame físico é recomendada na avaliação nutricional, a fim de identificar sinais clínicos de desnutrição prévios ou consequente ao tratamento.^(23)^

A tabela 8 apresenta os principais sinais clínicos que devem ser observados no exame físico e quais diagnóstico podem estar relacionados na faixa etária pediátrica.^(23)^

**Tabela 8:** Sinais clínicos e diagnósticos na faixa etária pediátrica

Área	Sinais clínicos	Diagnóstico
Cabelo	Perda de brilho natural	Kwashiorkor e, menos frequentemente, marasmo
	Fino e esparso	
	Quebradiço	
	Despigmentado	
	Fácil de arrancar	
	Sinal de bandeira	
Face	Seborreia nasolabial (pele seca ao redor das narinas)	Carência riboflavina
	Face edemaciada (lua cheia)	Kwashiorkor
	Palidez	Carência de ferro
Olhos	Conjuntiva pálida	Anemia
	Membranas vermelhas	
	Mancha de Bitot	Carência de vitamina A
	Xerose conjuntival	
	Xerose de córnea	
	Queratomalácia	
	Vermelhidão e fissuras de epicanto	Riboflaxina e piridoxina
	Arco córneo (anel branco ao redor dos olhos)	Hiperlipidemia
	Xantelasma (bolsas pequenas amareladas ao redor dos olhos)	
Lábios	Estomatite angular (lesões rosas e brancas ao redor da boca)	Riboflavina
	Escaras do ângulo	
	Queilose (avermelhamento ou edema de lábios)	
Língua	Língua escarlate e inflamada	Ácido nicotínico
	Língua magenta (púrpura)	Riboflavina
	Língua edematosa	Niacina
	Papila filiforme atrofiada e hipertrofiada	Ácido fólico e vitamina B12
Dentes	Esmalte manchado	Flúor
Gengivas	Esponjosas: sangrando e vazantes	Vitamina C
Glândulas	Aumento da tireoide	Iodo
	Aumento da paratireoide	Inanição
Pele	Xerose	Vitamina A
	Hiperqueratose folicular (pele em papel de areia)	
	Petéquias	Vitamina C
	Dermatose, pelagra	
	Equimoses em excesso	Vitamina K
	Dermatose cosmética descamativa	Kwashiorkor
	Dermatose vulvar e escrotal	Riboflavina
	Xantomas	Hiperlipidemia
Unhas	Em forma de colher, quebradiças e rugosas	Ferro
	Manchas pequenas brancas	Zinco

Fonte: Sociedade Brasileira de Pediatria (SBP). Avaliação nutricional da criança e do adolescente: manual de orientação. Departamento de Nutrologia. Rio de Janeiro:SBP; 2009. Capítulo 5. Avaliação da composição corporal. p.46-51.^(23)^

### Consumo alimentar

A assistência nutricional no TCTH deve levar em conta as demandas aumentadas de energia e nutrientes, assim como as restrições dietéticas a um paciente fragilizado que pode, muitas vezes, passar a ter uma dieta não saudável do ponto de vista de prevenção das doenças crônicas.^(58)^

Após o TCTH, a dieta passa a ter características particulares, uma vez que o aspecto sanitário é prioridade nessa fase, em detrimento dos aspectos nutricionais.^(59,60)^ O não consumo de alimentos crus e outras restrições nas escolhas alimentares resulta em deficiências dietéticas que devem ser compensadas com uso de suplementação dietética e/ou medicamentosa. Na fase pós-TCTH, é comum o aumento do consumo de alimentos pouco nutritivos, como guloseimas e frituras. Nesse sentido, a dieta não atende às recomendações qualitativas para prevenção de doenças crônicas.^(58)^ Tendo em vista que as recomendações nutricionais após o transplante aumentam, o paciente, muitas vezes, opta por alimentos não tão saudáveis, mas que cumprem o papel de preservação de peso.^(58)^

O consumo alimentar é avaliado por meio do recordatório alimentar de 24 horas para crianças maiores de 8 anos e, para as menores de 8 anos, é realizado com seus pais ou responsáveis. Tem o intuito de avaliar o consumo qualitativo e quantitativo de energia, micronutrientes e de macronutrientes,^(58,61)^ adequados à faixa etária, conforme as tabelas 9 a 11.

**Tabela 9:** Esquema alimentar para lactentes (zero a 12 meses) em aleitamento materno

Refeição	Idade (meses)			
	6-7	7-8	8-10	12
Manhã	Leite materno	Leite materno	Leite materno	Leite materno ou fórmula infantil
				Pão ou biscoito
Intervalo	Papa de fruta	Papa de fruta	Papa de fruta	Papa de fruta
Almoço	Leite materno	Papa salgada	Papa salgada	Papa ou refeição da família
Lanche	Papa de fruta	Papa de fruta	Papa de fruta	Papa de fruta
	Leite materno	Leite materno	Leite materno	Leite materno ou fórmula indicada para idade Pão ou biscoito
Jantar	Leite materno	Leite materno	Papa salgada	Papa ou refeição da família
Noite	Leite materno	Leite materno	Leite materno	Leite materno ou fórmula indicada para idade

Fonte: Sociedade Brasileira de Pediatria (SBP). Avaliação nutricional da criança e do adolescente: manual de orientação. Departamento de Nutrologia. Rio de Janeiro: SBP; 2009. Capítulo 5. Avaliação da composição corporal. p. 46-51;^(23)^ Silva AP, Nascimento AG. Zamberlan P. Manual de dietas e condutas nutricionais em pediatria. São Paulo: Atheneu; 2014. p. 21-6.^(24)^

**Tabela 10:** Esquema alimentar para lactentes (zero a 12 meses) em aleitamento artificial (fórmula infantil/láctea adequada para idade)

Refeição	Idade (meses)			
	6-7	7-8	8-10	12
Manhã	Fórmula infantil adequada para idade	Fórmula infantil adequada para idade	Fórmula infantil adequada para idade	Fórmula infantil/láctea adequada para idade
				Pão ou biscoito
Intervalo	Papa de fruta	Papa de fruta	Papa de fruta	Papa de fruta
Almoço	Fórmula infantil adequada para idade	Papa salgada	Papa salgada	Papa ou refeição da família
Lanche	Papa de fruta	Fórmula infantil adequada para idade	Papa de fruta	Papa de fruta
	Fórmula infantil adequada para idade		Fórmula infantil adequada para idade	Fórmula infantil/láctea adequada para idade
				Pão ou biscoito
Jantar	Fórmula infantil adequada para idade	Fórmula infantil adequada para idade	Papa salgada	Papa ou refeição da família
Noite	Fórmula infantil adequada para idade	Fórmula infantil adequada para idade	Fórmula infantil adequada para idade	Fórmula infantil/láctea adequada para idade

Fonte: Sociedade Brasileira de Pediatria (SBP). Avaliação nutricional da criança e do adolescente: manual de orientação. Departamento de Nutrologia. Rio de Janeiro: SBP; 2009. Capítulo 5. Avaliação da composição corporal. p.46-51;(23) Silva AP, Nascimento AG. Zamberlan P. Manual de dietas e condutas nutricionais em pediatria. São Paulo: Atheneu; 2014. p. 21-6.^(24)^

**Tabela 11:** Esquema alimentar para pré-escolar (1 a 6 anos), escolar (7 a 12 anos) e adolescentes (maiores de 12 anos)

Grupo alimentar	Porção	
	Pré-escolar e escolar	Adolescentes
Cereais, pães, tubérculos, raízes e massas (de preferência integrais)	5	5-9
Verduras e legumes	3	4-5
Frutas	3	4-5
Leites e derivados	3	3
Carnes ou ovos	2	1-2
Leguminosas (feijões, lentilha, grão de bico e ervilha)	1	1
Óleos e gorduras	1	1-2
Açúcar e doces	1	1-2

Fonte: Sociedade Brasileira de Pediatria (SBP). Avaliação nutricional da criança e do adolescente: manual de orientação. Departamento de Nutrologia. Rio de Janeiro: SBP; 2009. Capítulo 5. Avaliação da composição corporal. p.46-51;(23) Silva AP, Nascimento AG. Zamberlan P. Manual de dietas e condutas nutricionais em pediatria. São Paulo: Atheneu; 2014. p. 21-6.^(24)^

### Necessidades nutricionais

As recomendações energéticas e de nutrientes específicos para crianças alteram em função da idade. Como a energia deve ser direcionada para a manutenção das necessidades metabólicas e para o crescimento e desenvolvimento, a necessidade energética e de nutrientes em crianças e adolescentes é aumentada, tornando este um grupo de risco nutricional.^(62–66)^

Durante o período de transplante, muitos fatores podem alterar as necessidades nutricionais, como, por exemplo, regime de condicionamento com quimioterapia intensa, combinada ou não pela TBI, mucosite, inapetência, náuseas, vômitos, diarreia ou anorexia. Essas manifestações determinam implicações negativas no aporte calórico-proteico e prejuízo na absorção de nutrientes, além de levar ao aumento das necessidades calóricas ou ao catabolismo proteico.^(63,64,67–72)^ No geral, pacientes submetidos ao TCTH alogênico costumam ter imunidade mais comprometida e maior risco de complicações gerais do que os que realizam TCTH autólogo.^(63)^ Essas alterações muito agressivas provocam alterações metabólicas e nutricionais.^(71,73)^

A redução da ingestão calórica e proteica pode influenciar na função imune durante o estresse metabólico. Por isso, é de extrema importância a adequação das necessidades energéticas e proteicas, para manter um balanço nitrogenado adequado.^(74)^ Crescimento e desenvolvimento adequado de crianças e adolescentes estão relacionados a uma alimentação equilibrada, com oferta satisfatória de energia, macro e micronutrientes.^(69,74)^

Após a alta, os pacientes podem ter vários problemas relacionados à nutrição, como desnutrição devido à ingestão oral insuficiente ou distúrbios metabólicos. O suporte nutricional desempenha papel importante nos diferentes tipos de TCTH.^(75)^

Em suma, o apoio nutricional adequado antes e durante o transplante é uma medida de suporte potencialmente importante.^(76)^ As alterações nesses pacientes afetam principalmente o metabolismo de proteínas, energia e micronutrientes. Durante o TCTH, as necessidades energéticas e proteicas das crianças e dos adolescentes devem ser previstas para assegurar que o suporte nutricional seja adaptado e adequado às necessidades individuais da criança.^(77)^

### Necessidades energéticas

As necessidades energéticas dependem de fatores como o estado nutricional basal, a idade, o peso e o grau de estresse metabólico.^(74)^ Embora tenha sido demonstrado que o consumo de energia gasto por um paciente durante o TCTH possa diferir entre os pacientes alogênicos e os autólogos, existem estudos que identificam que as necessidades energéticas dos receptores de TCTH podem atingir 130% a 150% do gasto energético basal predito.^(70,73,78,79)^

Faz-se necessário conhecer os requisitos de energia de crianças durante o TCTH para assegurar um apoio nutricional adequado.^(77)^

As recomendações energéticas podem ser determinadas por equação-padrão de estimação e pelo uso de calorimetria indireta, sendo esta uma medida mais exata das necessidades energéticas.^(64,65,74)^

Bechard et al.,^(80)^ avaliaram dados antropométricos, ingestão alimentar, determinação de necessidades calóricas por equação de Schofield e gasto energético de repouso por meio da calorimetria entre o D0 do TCTH e D+30. Nesse período, foram observados redução do gasto energético de repouso, principalmente entre D0 e D+21, e consumo alimentar reduzido. Apesar de estudos controversos, os autores sugeriram, quando impossibilitado o uso da calorimetria indireta, utilizar a taxa metabólica basal e manter de 100% a 140% como meta calórica, evitando a oferta excessiva.

As necessidades energéticas devem ser estimadas com fórmulas-padrão (Tabela 12) e, em seguida, ajustadas de acordo com a evolução clínica.^(65)^

**Tabela 12:** Resumo das necessidades energéticas do paciente pediátrico submetido ao transplante de células-tronco hematopoiéticas.

Pré-TCTH		Condicionamento, aplasia medular, pega de medula (aproximadamente até D+35)		Pós-TCTH, alta hospitalar, acompanhamento ambulatorial	
1. Holliday e Segar (1957)		1. Scholfield (Murphy, 2004)		1. Holliday e Segar (1957)	
	Crianças de 0-10kg: 100kcal/kg Crianças de 10-20kg: 1.000kcal + 50kcal/kg para cada kg acima de 10kg Crianças com mais de 20kg: 1.500kcal + 20kcal/kg para cada kg acima de 20kg	Meninos			Crianças de 0-10kg: 100kcal/kg Crianças de 10-20kg: 1.000kcal + 50kcal/kg para cada kg acima de 10kg Crianças com mais de 20kg: 1.500kcal + 20kcal/kg para cadakg acima de 20kg
			<3 anos: (0,240 x peso (kg) – 0,127) x 239 3-10 anos: (0,095 x peso (kg) + 2,110) x 239 10-18 anos: (0,074 x peso (kg) + 2,7540) x 239 18-30 anos: (0,063 x peso (kg) + 2,896) x 239		
		Meninas			
			<3 anos: (0,244 x peso (kg) – 0,130) x 239 3-10 anos: (0,085 x peso (kg) + 2,033) x 239 10-18 anos: (0,056 x peso (kg) + 2,898) x 239 18-30 anos: (0,062 x peso (kg) + 2,036) x 239		
2. Aspen (2002)				2. Aspen (2002)	
	0-1 ano: 90-120kcal/kg 1-7 anos: 75-90kcal/kg 7-12 anos: 60-75kcal/kg 12-18 anos: 30-60kcal/kg 18-25 anos: 25-30kcal/kg				0-1 ano: 90-120kcal/kg 1-7 anos: 75-90kcal/kg 7-12 anos: 60-75kcal/kg 12-18 anos: 30-60kcal/kg 18-25 anos: 25-30kcal/kg
3. DRIs 2006				3. DRIs 2006	
	0-3 meses: (89 x peso (kg) -100) + 175 4-6 meses: (89 x peso (kg) -100) + 56 De 7-12 meses: (89 x peso (kg) -100) + 22 13-35 meses: (89 x peso (kg) -100) + 20				0-3 meses: (89 x peso (kg) -100) + 175 4-6 meses: (89 x peso (kg) -100) + 56 7-12 meses: (89 x peso (kg) -100) + 22 13-35 meses: (89 x peso (kg) -100) + 20
Meninos				Meninos	
	3-8 anos: 88,5 - 61,9 x idade + fator atividade x (26,7 x peso (kg) + 903 x altura) + 20 9-18 anos: 88,5 - 61,9 x idade + fator atividade x (26,7 x peso (kg) + 903 x altura) + 25				3-8 anos: 88,5 - 61,9 x idade + fator atividade x (26,7 x peso (kg) + 903 x altura) + 20 9-18 anos: 88,5 - 61,9 x idade + fator atividade x (26,7 x peso (kg) + 903 x altura) + 25
Meninas				Meninas	
	3-8 anos: 135,3 - 30,8 x idade + fator atividade x (10 x peso (kg) + 934 x altura) + 20 9-18 anos: 135,3 - 30,8 x idade + fator atividade x (10 x peso (kg) + 934 x altura) + 25				3-8 anos: 135,3 - 30,8 x idade + fator atividade x (10 x peso (kg) + 934 x altura) + 20 9-18 anos: 135,3 - 30,8 x idade + fator atividade x (10 x peso (kg) + 934 x altura) + 25
Fator atividade				Fator atividade	
	1 = atividades do dia a dia Meninos = 1,16; meninas = 1,13 – atividades do dia a dia + de 30 a 60 minutos de atividade moderada Meninos = 1,31; meninas = 1,26 – atividades do dia a dia + 60 minutos de atividade moderada Para crianças com baixo peso, utilizar P/E do percentil 50 ou do escore z = 0,00 Para crianças eutróficas, utilizar peso atual Para crianças com sobrepeso ou obesas, utilizar P/E no percentil 90 ou o escore z = +2,00 Esse ajuste em relação ao peso atual não deve ultrapassar 20%				1 = atividades do dia a dia Meninos = 1,16; meninas = 1,13 – atividades do dia a dia + de 30 a 60 minutos de atividade moderada Meninos = 1,31; meninas = 1,26 – atividades do dia a dia + 60 minutos de atividade moderada Para crianças com baixo peso, utilizar P/E do percentil 50 ou do escore z = 0,00 Para crianças eutróficas, utilizar peso atual Para crianças com sobrepeso ou obesas, utilizar P/E no percentil 90 ou o escore z = +2,00. Esse ajuste em relação ao peso atual não deve ultrapassar 20%

Fonte: Pine M, Wang L, Harrell FE Jr, Calder C, Manes B, Evans M, et al. The effect of obesity on outcome of unrelated cord blood transplant in children with malignant diseases. Bone Marrow Transplant. 2011;46(10):1309-13;^(19)^ White M, Murphy AJ, Hastings Y, Shergold J, Young J, Montgomery C, et al. Nutritional status and energy expenditure in children pre-bone-marrow-transplant. Bone Marrow Transplant. 2005;35(8):775-9;^(77)^ Bechard LJ, Feldmann HA, Venick R, Gura K, Gordon C, Sonis A, et al. Attenuation of resting energy expenditure following hematopoietic STC in chlidren. Bone Marrow Transplant. 2012;47(10):1301-6.^(80)^

TCTH: transplante de células-tronco hematopoiéticas; DRI: *Dietary Reference Intake* P/E: peso/ estatura.

Durante a nutrição parenteral, recomenda-se que as necessidades energéticas sejam 80% a 90% das recomendações para a nutrição enteral.^(65)^

### Necessidades proteicas

As necessidades de proteína na pediatria também são aumentadas^(59,74)^ e devem ser adequadas de maneira qualitativa e quantitativa.^(65)^ Variam de acordo com idade e peso.^(57)^ Essas quantidades adicionais de proteínas se destinam a restaurar ou preservar a massa magra corporal e fornecer substrato para o estado hipercatabólico após o TCTH. É importante evidenciar que, na vigência de alterações da função renal ou hepática, essas necessidades devem ser modificadas e ajustadas individualmente.^(74)^

A oferta proteica reduzida pode influenciar negativamente na função imunológica durante o período de estresse metabólico. Por isso, é importante adequar as necessidades de proteína, para um balanço nitrogenado igual a zero.^(63)^ O apoio nutricional que é adequado para prevenir a perda de proteínas, mas que não fornece calorias excessivas e não provoca acúmulo de gordura, é essencial para a sobrevivência dos pacientes submetidos a transplante.^(64)^

As necessidades proteicas são estimadas (Tabela 13), a fim de promover substrato para o reparo dos tecidos após a citorredução e para diminuir a perda de massa magra após o TCTH. No geral, seus valores variam entre 1,4g/kg a 3,0g/kg de peso.^(71,73)^

**Tabela 13:** Resumo das necessidades proteicas do paciente pediátrico submetido a transplante de células-tronco hematopoiéticas

Pré-TCTH, pós-TCTH, alta hospitalar e acompanhamento ambulatorial		Condicionamento, aplasia medular, pega de medula (aproximadamente até D+35)	
Conforme idade		Conforme idade*	
	De neonatos até 2 anos: 2,5-3,0g/kg/dia		De 0-6 anos: 2,5-3,0g/kg de peso atual
	Crianças de 2 a 11 anos: 2,0g/kg/dia		De 7-10 anos: 2,4g/kg de peso atual
	Adolescentes (acima de 12 anos): 1,5-2,0g/kg/dia		De 11-14 anos: 2,0g/kg de peso atual
	Em casos de perda de peso e desnutrição, sugere-se um incremento de 15% a 50% das recomendações de proteína		De 15-18 anos: 1,8g/kg de peso atual
	Para crianças com baixo peso, utilizar P/E no percentil 50 ou o escore-z = 0,00		
	Para crianças eutróficas, utilizar peso atual		
	Para crianças com sobrepeso ou obesas, utilizar P/E no percentil 90 ou o escore-z = +2,00		
	Esse ajuste em relação ao peso atual não deve ultrapassar 20%		

Fonte: Brasil. Ministério da Saúde. Instituto Nacional de Câncer José de Alencar Gomes da Silva (INCA). Consenso nacional de nutrição oncológica. 2a ed. rev. ampl. atual. Rio de Janeiro: INCA; 2015. p.182 [citado 2020 Jul 4]. Disponível em: https://www.inca.gov.br/sites/ufu.sti.inca.local/files/media/document/consenso-nacional-de-nutricao-oncologica-2-edicao-2015.pdf;^(20)^ Mendes TG, Benedetti FJ. Fatores nutricionais associados ao câncer em crianças e adolescentes. Disciplinarum Scientia. 2013;14(2):265-72;^(61)^ Sociedade Brasileira de Nutrição Parenteral e Enteral. Associação Brasileira de Cirurgia Pediátrica. Sociedade Brasileira de Clínica Médica. Associação Brasileira de Nutrologia. Projeto Diretrizes. Recomendações nutricionais para crianças em terapia nutricional enteral e parenteral. São Paulo: AMBCFM; 2011. p. 1-16 [citado 2019 Dez 10]. Disponível em: https://diretrizes.amb.org.br/_BibliotecaAntiga/recomendacoes_nutricionais_para_criancas_em_terapia_nutricional_enteral_e_parenteral.pdf.^(65)^

* Em casos de alterações da função renal ou hepática, essas necessidades devem ser modificadas e ajustadas individualmente.

TCTH: transplante de células-tronco hematopoiéticas; P/E: peso/estatura.

### Necessidades de micronutrientes

As vitaminas são consideradas importantes para o crescimento e variam de acordo com a idade.^(65)^

Os níveis de vitaminas, tanto as hidrossolúveis quanto as lipossolúveis, estão alterados nos pacientes submetidos ao TCTH, como resultado da baixa ingestão ou da má absorção. Outros fatores interferem também, como o uso da ciclosporina e a radioterapia, que provocam aumento das necessidades de vitaminas.^(71,75)^

Para todos os pacientes que não recebem nutrição parenteral, é prudente fornecer um multivitamínico oral e um suplemento mineral por causa da ingestão restrita de uma dieta normal, muitas vezes por um período prolongado.^(81)^

Alguns micronutrientes merecem destaque no TCTH:

- Vitamina D ou colecalciferol: as deficiências de vitamina D são prevalentes em pacientes com DECH. Essa vitamina tem função endócrina como um regulador-chave da absorção de cálcio e homeostase óssea e também de regulação do sistema imunológico. Ela é necessária para o desenvolvimento e manutenção do tecido ósseo e a manutenção da homeostase do cálcio e do fósforo. Sem a ação da vitamina D, apenas 10% a 15% do cálcio ingerido e aproximadamente 60% do fósforo seriam absorvidos.^(71,73)^ Pacientes submetidos ao TCTH apresentam insuficiência ou deficiência de vitamina D, possivelmente pela redução alimentar pós-transplante e por evitar a exposição solar.^(71,73)^
- Vitamina K: a deficiência dessa vitamina é importante no TCTH, pois aumenta o risco de hemorragia grave. A etiologia de deficiência de vitamina K é, na maioria das vezes, multifatorial e geralmente resulta do antagonismo da droga, da disfunção hepática, da má absorção, da anorexia e/ou da ingestão inadequada de nutrientes.^(82)^
- Zinco: a deficiência de zinco é prevalente em pacientes após o TCTH alogênico, especialmente naqueles com diarreia. O zinco é necessário para o bom funcionamento genético, a imunidade, a formação de glóbulos vermelhos, os órgãos, a função muscular e óssea e a estabilidade de membrana celular, além do crescimento das células, sua divisão e diferenciação genética. Ele também tem papel importante no contexto da resposta metabólica a lesões e à cicatrização de feridas.^(83)^ O sulfato de zinco aparece como agente promissor para a prevenção de mucosite, que ocorre durante o TCTH, por ser um cofator essencial em vários processos celulares, como a síntese de DNA, a polimerase de RNA e a transcriptase reversa, possuindo efeito sobre a cicatrização de feridas, o crescimento e a imunidade.^(83)^ As manifestações clínicas da deficiência de zinco, como anorexia, erupção cutânea, diarreia e infecções, também são comuns.^(69,71)^
- Ferritina: é uma proteína intracelular de armazenamento e transporte do ferro, ou seja, está diretamente relacionada à ingestão adequada deste micronutriente, sendo encontrada em praticamente todas as células do organismo, mas, principalmente, em hepatócitos e fluidos orgânicos. No plasma, ela está presente em pequenas concentrações, correlacionando-se com estoques de ferro do organismo. Sua função primária é acumular o ferro intracelular, que está em sua forma biologicamente disponível para os processos celulares vitais, protegendo proteínas, lipídios e DNA, além de exercer papel importante em condições inflamatórias.^(71)^

As necessidades de micronutrientes para pacientes do TCTH não estão totalmente estabelecidas, e, de maneira geral, devem-se seguir as recomendações para população saudáveis da *Dietary Reference Intake* (DRI), porém alguns itens merecem destaque, conforme demonstrado na tabela 14.

**Tabela 14:** Ingestão diferencial recomendada de alguns micronutrientes para crianças durante o transplante de células-tronco hematopoiéticas

Grupo	Vitamina D		Vitamina K	Zinco			Ferro		
	Ingestão adequada	Nível máximo de ingestão tolerada	Ingestão adequada	Necessidade média estimada	Ingestão adequada	Nível máximo de ingestão tolerada	Necessidade média estimada	Ingestão adequada	Nível máximo de ingestão tolerada
0-6 meses	5	25	2,0		2	4	ND	0,27	40
7-12 meses	5	25	2,5	2,2	3	5	6,9	11	40
1-3 anos	5	50	30	2,2	3	7	3	7	40
4-8 anos	5	50	55	4,0	5	12	4,1	10	40
9-13 anos	5	50	60	7,0	8	23	5,9	8	40
14-18 anos	5	50	75	8,5	11	34	7,7	11	45
19-30 anos	5	50	120				6	8	45

Fonte: Sociedade Brasileira de Nutrição Parenteral e Enteral. Associação Brasileira de Cirurgia Pediátrica. Sociedade Brasileira de Clínica Médica. Associação Brasileira de Nutrologia. Projeto Diretrizes. Recomendações nutricionais para crianças em terapia nutricional enteral e parenteral. São Paulo; AMBCFM; 2011. p. 1-16 [citado 2019 Dez 10]. Disponível em: https://diretrizes.amb.org.br/_BibliotecaAntiga/recomendacoes_nutricionais_para_criancas_em_terapia_nutricional_enteral_e_parenteral.pdf;^(65)^ Padovani RM, Amaya-Farfán J, Colugnati FA, Domene SM. Dietary reference intakes: application of tables in nutritional studies. Rev Nutr. 2006;19(6):741-60.^(84)^

ND: não determinado.

### Complicações nutricionais mais comuns

A terapia imunossupressora utilizada é agressiva e com alta toxicidade, o que gera sinais e sintomas clínicos, como náuseas, vômitos, dor abdominal, alteração do paladar, mucosite orofaríngea, odinofagia, xerostomia, esofagite, diarreia, infecções, sangramentos e anemia.^(85–89)^ Essas alterações podem permanecer por semanas após o transplante, trazendo consequências negativas ao paciente, principalmente no aporte calórico, proteico e de nutrientes, gerando perda progressiva do estado nutricional.^(20)^

Além das necessidades energéticas aumentadas nesses pacientes, com o objetivo de manter um adequado desenvolvimento nessa faixa etária, os profissionais devem estar atentos aos efeitos catabólicos provenientes da enfermidade de base, assim como aos sinais e sintomas referentes aos tratamentos, que podem levar o paciente a uma menor ingestão calórica.^(90)^

A rotina diária desses pacientes torna-se profundamente afetada por esses sintomas, o que justifica a necessidade de seu manejo eficaz por parte do profissional de saúde.^(91)^

### Náuseas e vômitos

O diagnóstico da náusea é clínico e baseado na história relatada pelo paciente e seus familiares, uma vez que se trata de uma sensação subjetiva. É importante questionar o paciente a respeito de seu desejo de se alimentar, sensação de fraqueza, eventual dificuldade mecânica para deglutir, presença de sede e/ou fome, reação a determinados alimentos e odores e fatores desencadeantes do sintoma. A avaliação do estado nutricional do paciente e dos sinais de desidratação pode contribuir para o diagnóstico de náuseas crônicas e/ou intensas. O diagnóstico de vômito também se baseia na história clínica, sendo importante a quantificação dos episódios e do volume em cada um deles.^(92)^

Durante o TCTH, as náuseas e vômitos podem ser associadas ao protocolo de condicionamento (quimioterapia ou radioterapia), bem como a outras causas, como uso de medicamentos (antimicrobianos e opioides); infecções sistêmicas; alterações metabólicas, como hipercalemia e hiponatremia; insuficiência adrenocortical; aumento da pressão intracraniana; irritações e/ou ulcerações gástricas; constipação; obstrução intestinal, como refluxo gastresofágico e estase gástrica, além de alterações psicológicas, como ansiedade e estresse emocional. Muitos pacientes vivenciam sofrimento devido a náuseas e vômitos, o que pode causar desconforto importante, como dor, desidratação, soluço, azia e anorexia.^(92)^ A atenção e o manejo adequado desses sintomas são fundamentais para proporcionar conforto e qualidade de vida às crianças ou aos adolescentes e a suas famílias durante todas as fases do tratamento.^(92)^

Para que se obtenha sucesso no manejo e no controle de náuseas e vômitos na população pediátrica, faz-se necessária uma avaliação completa, considerando-se as possíveis causas, como o relato das crianças ou dos adolescentes e de seus cuidadores. A história pregressa detalhada da náusea e vômito, a frequência desses sintomas, os períodos em quem estão mais presentes, sua descrição e intensidade, como quantidade e característica do conteúdo expelido, os horários dos episódios, os fatores associados e/ou predisponentes são de extrema importância para a eficácia do tratamento a ser escolhido.^(93)^

As medidas dietéticas devem ser adequadas às necessidades do indivíduo, às suas preferências e a seus hábitos alimentares,^(94)^ e, ao serem usadas juntamente dos medicamentos antieméticos, podem ajudar a reduzir a frequência e a dose deles.^(93)^ Algumas medidas simples podem auxiliar no controle das náuseas e vômitos, como o fracionamento da dieta em pequenas refeições em intervalos menores, a realização das refeições em ambientes tranquilos e arejados, a manutenção de horários estabelecidos para as refeições e a oferta de pequenas quantidades de carboidratos e de alimentos que sejam da preferência do paciente.^(91,92)^ Além disso, é importante que o paciente mantenha sua cabeça elevada por até 1 a 2 horas após a ingestão de alimentos; evitae preparações a temperaturas extremas, preferindo aquelas em temperatura ambiente ou alimentos frios; evite que o paciente fique próximo à cozinha na hora de preparo da refeição, impedindo, assim, que os cheiros dos alimentos durante a cocção acentuem as náuseas, e evite frituras, alimentos gordurosos, condimentados, salgados, ácidos, açucarados e com odor forte.^(91)^

### Doença do enxerto contra o hospedeiro na criança

A DECH é uma das principais complicações pós-transplante de TMO. Ela pode ser classificada em aguda (DECHa) e DECHc. A DECHa classicamente ocorre nos primeiros 100 dias pós-TMO e afeta principalmente a pele, o fígado e o trato gastrintestinal (TGI). A DECHc classicamente se inicia após os primeiros 100 dias do TMO e pode acometer um ou mais órgãos. Os sítios principais de acometimento da DECHc são pele, fígado, boca, olhos, sistema musculoesquelético, pulmões e genitais. Ela pode durar de vários meses a anos e se assemelha a uma doença autoimune, caracterizada pela presença de inflamação e, posteriormente, de fibrose dos tecidos e órgãos acometidos.^(95,96)^

No TGI, as principais manifestações clínicas da DECH, tanto aguda quanto crônica, são náuseas, vômitos, anorexia, diarreia, dor abdominal e disfagia. Dependendo da intensidade dos sintomas, os pacientes podem desenvolver formas leves, moderadas e graves. Esses quadros podem ainda ser agravados pelo tratamento imunossupressor, que consiste, na maioria dos casos, no uso de altas doses de corticosteroides, associados ou não ao uso dos inibidores de calcineurina (ciclosporina e tacrolimus). Esses medicamentos podem exacerbar os sintomas, seja por efeito direto, seja por favorecerem a ocorrência de infecções. Como consequência, os pacientes desenvolvem quadros de intensa desnutrição proteico-calórica geralmente multifatorial, pela redução da ingestão alimentar, por défice de absorção e pelo aumento do gasto enérgico. Além disso, como consequência da DECH e de seu tratamento, ocorrem alterações do metabolismo dos carboidratos, dos lipídios e das proteínas. Nesse contexto, a terapia nutricional é de extrema importância como suporte ao tratamento para combater os efeitos deletérios da DECH e contornar os efeitos adversos dos medicamentos.^(76,83,95,96)^

Os pacientes com DECH têm dificuldade para ingerir alimentos por várias razões, dependendo do órgão envolvido. Frequentemente, necessitam de modificações na dieta, de suplementos orais e de terapia de suporte nutricional (TSN) para prevenir ou tratar a desnutrição.^(95)^ Segundo Bassim et al.,^(97)^ as principais indicações para o início da TSN são náuseas e vômitos não controlados, diarreia volumosa, dor na mucosa oral e esofágica, disfagia, disgeusia, xerostomia, anorexia, saciedade precoce e perda de peso. Em especial, a DECHa de TGI e a DECHc oral, gastrintestinal e pulmonar produzem desnutrição grave e levam a um comprometimento da capacidade funcional e da qualidade de vida dos pacientes – daí a necessidade do início precoce de TSN.

De acordo com o Consenso de Nutrição Oncológica do Instituto Nacional de Câncer José de Alencar,^(20)^ algumas intervenções nutricionais podem ser orientadas para melhorar e controlar os sintomas gastrintestinais.

Independentemente do tipo e do grau da DECH, quando o paciente apresenta ingestão alimentar abaixo de 70% das necessidades energéticas nos últimos 3 dias e sintomas que prejudiquem a alimentação adequada, é importante intervir com o uso de suplementos nutricionais hipercalóricos e hiperproteicos.^(20)^

Caso a ingesta alimentar esteja abaixo de 60% das necessidades energéticas nos últimos 3 dias ou a via oral estiver contraindicada, a terapia nutricional enteral (TNE) pode ser prescrita.^(20)^ A via enteral, se tolerável e clinicamente possível, pode ser escolhida, por manter a função digestiva e a integridade da barreira mucosa, prevenindo a translocação bacteriana no trato digestivo.^(83)^

Segundo revisão sistemática de Baumgartner et al.,^(76)^ vários estudos têm comparado a TNE com terapia nutricional parenteral (TNP), mostrando resultados superiores para a via enteral e moderada a alta tolerância à sonda, sendo a TNP recomendada somente em casos de insuficiência gastrintestinal. A TNE é contraindicada na presença de instabilidade hemodinâmica e/ou piora da dor abdominal, distensão abdominal, mucosite, diarreia, vômitos incoercíveis, íleo paralítico e sangramento intestinal.^(20)^

Contudo, especialmente entre os pacientes com DECHa de TGI, na maioria das vezes, não há tolerância à nutrição oral ou enteral. Na presença de diarreia grave (>1L ao dia), o jejum de alimentos por via oral durante dias a semanas é imprescindível para aliviar as queixas gastrintestinais. O suporte nutricional consiste em TNP.^(82)^ Estudos demonstram que pacientes com DECH graus III a IV recebem mais TNP que pacientes com DECH graus zero a II, não estando isentos de apresentarem complicações clínicas, relacionadas com a progressão do número de dias que recebem a TNP.^(74)^

Após o volume da diarreia reduzir durante o DECH intestinal (<500mL ao dia), a alimentação via oral é reiniciada, mas certos alimentos podem ser mais bem tolerados do que outros.^(83)^ Muitos centros baseiam a terapia nutricional em uma dieta de fases, na qual, de acordo com os sintomas e a tolerância do paciente, evolui-se ou regride-se a dieta. Segundo as diretrizes gerais da *Seattle Cancer Care Alliance*,^(98)^ é recomendado iniciar a evolução de dietas específicas, com quantidades limitadas de gorduras, fibras, lactose, alimentos ácidos e irritantes do TGI, que devem ser reintroduzidos de forma gradual, observando a tolerância do paciente, conforme tabela 15.^(99)^

**Tabela 15:** Progressão da terapia nutricional para doença do enxerto contra o hospedeiro de trato gastrintestinal baseado no protocolo da *Seattle Cancer Care Alliance*

Fase	Sintomas	Terapia nutricional
Primeira fase: repouso intestinal	Grande volume de diarreia aquosa; cólicas intestinais; depleção da albumina sérica; diminuição do trânsito intestinal; obstrução intestinal; náusea e vômito	Somente nutrição parenteral
Segunda fase: introdução de alimentação oral	Volume de diarreia menor que 500mL/dia; diminuição das cólicas intestinais; melhora do tempo de trânsito intestinal; diminuição das náuseas e vômitos	Nutrição parenteral + dieta oral líquida isosmótica, sem resíduos, sem lactose, sem ácidos e hipogordurosa
Terceira fase: introdução de alimentos sólidos	Ausência ou diminuição das cólicas e fezes mais consistentes	Dieta oral: introdução de alimentos sólidos, sem resíduos, sem lactose, hipogordurosa e sem ácidos e irritantes gástricos
Quarta fase: expansão da dieta	Ausência ou diminuição das cólicas e fezes mais consistentes	Pobre em fibras, lactose, ácidos, irritantes gástricos e gordura, de acordo com a tolerância do paciente
Quinta fase: introdução de dieta regular	Ausência de cólicas e fezes em consistência normal	Introdução gradual e de acordo com a tolerância do paciente de alimentos ácidos, irritantes gástricos, com fibras, lactose e gordura

Fonte: Flowers ME, McDonald G, Carpenter P, Boeckh M, Sanders J, Deeg J, et al. Long-term follow-up after hematopoietic stem cell transplant general guidelines for referring physicians. Seattle (WA): Fred Hutchinson Cancer Research Center/ Seattle Cancer Care Alliance; 2019. p.88 [cited 2019 Nov 26]. Available from: https://www.fredhutch.org/content/dam/public/Treatment-Suport/Long-Term-Follow-Up/physician.pdf;^(98)^ Viani K, Leao AC, Mantovani LF. Transplante de células tronco-hematopoieticas. In: Viani K, Oliveira V, Nabarrete J, Silva AP, Feferbaum R. Nutrição e câncer infanto-juvenil. 1a ed. São Paulo: Manole; 2017. p. 188-201.^(99)^

A imunossupressão com corticosteroides constitui a base da terapia de primeira linha na DECHa e na DECHc.^(100)^ O uso dos inibidores de calcineurina também é comum na prevenção e no tratamento desses dois tipos de DECH.^(83)^

Durante o tratamento com altas doses de glicocorticoides e/ou inibidores de calcineurina, é importante a orientação adequada ao paciente. São recomendadas refeições frequentes e fracionadas, alimentação rica em fibras solúveis e insolúveis, dieta hiperproteica, com redução de carboidratos simples e de alto índice glicêmico, redução de sódio, boa ingestão hídrica e consumo adequado de alimentos que são fontes de vitamina D, cálcio, magnésio e zinco e, se necessária, a suplementação desses elementos.^(99,101)^

A monitorização dos níveis de vitaminas e minerais é de suma importância na DECH de TGI, pela presença de quadros de diarreia e má absorção dos nutrientes que esses pacientes apresentam.^(102)^ Devem-se observar com mais atenção os níveis de vitamina B_12_, devido à redução do fator intrínseco dos pacientes com DECH de estômago. Da mesma forma, devem ser notados os níveis de zinco, por seu papel na manutenção da integridade da mucosa intestinal e na acuidade do paladar, atuando na melhora da disgeusia.^(83)^

A suplementação de outros nutrientes, como ômega 3, glutamina, arginina e nucleotídeos, parece ser benéfica nos pacientes com DECH, porém os estudos são escassos e com pequenas populações, sendo necessárias mais evidências científicas para segurança de prescrição.^(83)^

A DECHa e a DECHc, complicações que cursam com alterações nutricionais graves, tornam os pacientes suscetíveis à desnutrição. Se não tratadas adequadamente, reduzem a sobrevida e a qualidade de vida dos pacientes, devendo ser feito o acompanhamento do indivíduo acometido, bem como a intervenção nutricional.

### Mucosite

A mucosite oral é uma alteração inflamatória^(103–105)^ e representa uma das principais complicações associadas ao TCTH, ocorrendo em 60% a 80% dos casos submetidos a regimes de condicionamento mieloablativo.^(106,107)^

Apesar de poder ocorrer em todo o trato digestório, a mucosite em cavidade oral associa-se a uma das experiências mais desconfortáveis e dolorosas do tratamento,^(103,108)^ sendo, muitas vezes, a principal responsável pelo desconforto no período inicial do TCTH. Nessa fase, é comum ocorrer redução na ingesta alimentar, prejuízos nutricionais, perda ponderal, complicações infecciosas, necessidade de nutrição parenteral, uso de opioides e, por vezes, o prolongamento do período de internação.^(106,109)^

Os critérios para classificação da mucosite mais comumente utilizados são os definidos pela OMS e pelo *National Cancer Institute* (NCI),^(110)^ que levam em consideração sinais e sintomas como dor, eritema, ulcerações, função e capacidade de alimentação (Tabela 16).

**Tabela 16:** Classificação da mucosite oral por escala de graduação

Escala de graduação	Grau					
	0	1	2	3	4	5
NCI CTCAE	Ausência	Assintomático ou sintomas leves; sem indicação de intervenções	Dor moderada; tolerância à ingesta oral	Dor severa; impossibilidade à ingesta oral	Lesões com risco iminente de morte; necessidade de intervenções urgentes	Morte
OMS	Ausência	Dor e eritema	Úlceras; mantêm ingesta de alimentos sólidos	Úlceras; mantêm apenas ingesta de líquidos	Impossibilidade de ingestão oral	

Fonte: Chaudhry HM, Bruce AJ, Wolf RC, Litzow MR, Hogan WJ, Patnaik MS, et al. The incidence and severity of oral mucositis among allogeneic hematopoietic stem cell transplantation patients: a systematic review. Biol Blood Marrow Transplant. 2016;22(4):605-16. Review.^(109)^

NCI: *National Cancer Institute*; CTCAE: *Common Terminology Criteria for Adverse Events*; OMS: Organização Mundial da Saúde.

A mucosite é clinicamente caracterizada por dor, edema, eritema, ulcerações e formação de pseudomembranas. Essas alterações estão associadas à limitação das funções da cavidade oral, da fala e da deglutição, impactando na qualidade de vida do paciente.^(103–105,111)^

A dor na mucosite oral apresenta etiologia tanto de origem nociceptiva, quanto neuropática. A nocicepção mecânica resulta na disfunção oral dos pacientes com mucosite oral. A dor neuropática é causada pela sensibilização neuronal adquirida pela ação dos quimioterápicos e mediadores inflamatórios (glutamato, neuropeptídeos e citocinas pró-inflamatórias, como, por exemplo: interleucina – IL-1, IL-6 e fator de necrose tumoral alfa – TNF-α), resultando na piora da sensibilização neuronal.^(103)^

O curso clínico da mucosite é normalmente previsível, com os primeiros sinais aparecendo entre 3 a 4 dias após o início do condicionamento e com o surgimento das ulcerações logo em seguida, apresentando maior intensidade entre os dias 7 a 14, geralmente com resolução espontânea na semana seguinte ou concomitante à enxertia.^(106,111)^

A incidência e a severidade dos casos de mucosite estão associadas a fatores relacionados ao tratamento,^(112)^ como condicionamentos mieloablativos,^(113)^ em especial regimes de condicionamento contendo TBI, altas doses de melfalano e bulssufano e utilização de metotrexato na profilaxia para DECH.^(105,109,114)^

A patogênese da mucosite é multifatorial, e os modelos atuais incluem eventos biológicos diferentes durante a apresentação da mucosite, representados por um processo cronológico de cinco diferentes fases, porém interdependentes, que envolvem, na ordem, iniciação/morte celular; geração de agentes oxidativos/ativação de citocinas inflamatórias; amplificação e sinalização; ulceração e recuperação/cura, conforme figura 1.^(104,106,113,115)^

**Figura 1:** Fases do desenvolvimento da mucosite

O manejo da mucosite recai sobre o cuidado de sintomas e a prevenção de complicações, que incluem o controle da dor, o suporte nutricional, a profilaxia e o tratamento de infecções secundárias, conforme tabela 17.^(105,116)^

**Tabela 17:** Cuidados gerais para profilaxia da mucosite

Cuidados	Ações
Recomendações de higiene oral e cuidados orais básicos	Escovar os dentes 2 a 3 vezes ao dia com escovas
	de cerdas macias ou ultramacias, com pastas
	de dentes fluoradas (em caso de queimação,
	utilizar pastas infantis, minimamente aromatizadas)
	Utilizar fio dental após cada refeição
	Realizar bochechos com soluções suaves por 4 a 6 vezes ao dia
	(água estéril ou bicarbonato de sódio)
	e considerar uso de hidratante oral
	Evitar enxuguadores bucais que contenham álcool ou peróxido e pastas de dente com lauril sulfato de sódio
Avaliação oral por especialista	Deve ser feita antes do início do tratamento
	quimioterápico ou radioterápico,
	com avaliação perodontal e pré-operatório,
	além de tratamento, conforme apropriado
	Devem ser retirados possíveis fatores traumáticos
	(origem dentária ou protética)
	e deve ser mantido regular acompanhamento,
	durante e após o tratamento
Propostas terapêuticas	Fatores de crescimento e citocinas
	Agentes de revestimentos e analgésicos
	Terapia com laser
	Crioterapia

Fonte: Bensinger W, Schubert M, Ang KK, Brizel D, Brown E, Eilers JG, et al. NCCN task force report. prevention and management of mucositis in cancer care. J Natl Comp Canc Netw. 2008;6 (Suppl 1):S1-21; quiz S22-4. Review;^(111)^ Cutler C, Li S, Kim HT, Laglenne P, Szeto KC, Hoffmeister L, et al. Mucositis after allogeneic hematopoietic stem cell transplantation: a cohort study of methotrexate- and non-methotrexate-containing graft-versus-host disease prophylaxis regimens. Biol Blood Marrow Transplant. 2005;11(5):383-8;^(115)^ Lalla RV, Sonis ST, Peterson DE. Management of oral mucositis in patients who have cancer. Dent Clin North Am. 2008;52(1):61-77, viii. Review;^(116)^ Vigarios E, Epstein JB, Sibaud V. Oral mucosal changes induced by anticancer targeted therapies and immune checkpoint inhibitors. Support Care Cancer. 2017;25(5):1713-39. Review;^(117)^ Lopes NN, Plapler H, Lalla RV, Chavantes MC, Yoshimura EM, da Silva MA, et al. Effects of low-level laser therapy on collagen expression and neutrophil infiltrate in 5-fluorouracil-induced oral mucositis in hamsters. Lasers Surg Med. 2010;42(6):546-52;^(118)^ Oberoi S, Zamperlini-Netto G, Beyene J, Treister NS, Sung L. Effect of prophylactic low level laser therapy on oral mucositis: a systematic review and meta-analysis. PLoS One. 2014;9(9):e107418. Review;^(119)^ Saadeh CE. Chemotherapy- and radiotherapy-induced oral mucositis: review of preventive strategies and treatment. Pharmacotherapy. 2005;25(4):540-54. Review.^(120)^

Assim, após a mucosite oral instalada, o tratamento deve ser apenas paliativo. A glutamina (L-glutamina ou L-alanil-L-glutamina) é utilizada em altas doses por células de divisão rápida, por isso tem se estudado seu uso na prevenção e no tratamento da mucosite oral, embora sua relevância ainda não tenha sido comprovada.^(99)^

A crioterapia é amplamente utilizada na prevenção da mucosite provocada por quimioterápicos que possuem curto tempo de meia-vida, como as doses em bólus de 5-fluorouracil ou a administração de melfalano em dose elevada em regimes de condicionamento de TCTH. Ela é geralmente realizada com água gelada ou raspas de gelo. Na pediatria, é possível incentivar tal prática com a oferta de sorvetes e picolés, ou utilizar a chupeta ou o bico da mamadeira congelados, molhando-os em água potável e, depois, acondicionando em *freezer*, ou até mesmo congelar leite materno ou fórmula infantil em forma de picolés e ofertar para a criança 10 minutos antes do início e até o término da infusão do medicamento.^(99)^

Após a mucosite oral instalada, a terapia nutricional oral consiste em adaptações na dieta, conforme a tabela 18, e/ou no uso de nutrição por via parenteral. Se o paciente já possuir uma sonda nasoenteral, pode-se utilizar a dieta por via enteral. Caso contrário, não é indicada a passagem de sonda nasoenteral em pacientes com mucosite oral grave instalada.

**Tabela 18:** Modificações alimentares na dieta oral para pacientes com mucosite oral

Modificações na dieta para a mucosite oral
Alimentos e líquidos na temperatura ambiente, morna ou fria
Preparações em consistência pastosa e/ou macia
Evitar alimentos ácidos, bebidas alcoólicas e/ou gaseificadas
Evitar temperos fortes e picantes e o excesso do sal
Consumo de alimentos com molhos

### Anorexia e caquexia

Anorexia (perda de apetite) é um concomitante comum em pessoas com câncer. A anorexia em câncer tem muitas causas, mas a primária é, muitas vezes, o aumento de citocinas pró-inflamatórias ou de lactato. Esses dois fatores, então, modulam as cascatas do neurotransmissor do sistema nervoso central.^(121)^

A caquexia do câncer é uma síndrome multifatorial que abrange um espectro desde a perda de peso inicial (pré-caquexia) até um estado de incapacidade grave incompatível com a vida. As principais características definidoras da caquexia em seres humanos são perda de peso, ingestão reduzida de alimentos e inflamação sistêmica. O suporte nutricional na caquexia pode estabilizar e melhorar o estado nutricional, a função e a qualidade de vida.^(121)^

Existe um grande estudo prospectivo que mostrou que pacientes pós-TMO foram acompanhados por até 6 anos e apresentavam índice de massa magra inferior ao Grupo Controle de indivíduos saudáveis. Aqueles portadores de DECHc e em uso de corticoide foram os mais afetados.^(122)^

Fatores como vômitos e náuseas persistentes, constipação, diarreia (relacionada à quimioterapia, infecção e DECH), mucosite (relacionada à quimioterapia, infecção e DECH), mudanças e perdas no paladar, assim como alterações olfativas e distúrbios metabólicos, podem levar o paciente, no momento do TCTH, à baixa ingestão alimentar e perda de peso.^(123)^

Mais que qualquer outro sintoma, a anorexia é vista como o sintoma principal do início dos sinais clínicos e metabólicos que precedem a caquexia, podendo causar perda de peso importante e involuntária.^(124)^

No paciente em TCTH, fatores socioeconômicos, médicos e físicos podem levar à síndrome anorexia-caquexia, mesmo após a alta hospitalar.^(121)^ Dessa forma, sinais e sintomas, como anorexia, perda de peso, alterações de exames bioquímicos e alterações de composição corporais, devem ser acompanhados frequentemente, para que ocorra intervenção nutricional o mais precoce possível, evitando interferência no *perfomance status* e na qualidade de vida do paciente, bem como no que se refere ao risco aumentado de complicações, como DECH.^(125–127)^

Assim, é necessário pensar em todos os sintomas presentes e em como tratá-los. O trabalho da equipe de nutrição é mostrar ao paciente e aos seus familiares a importância de se alimentar, porém, sem cobranças e sem nunca forçar o paciente a comer. O objetivo é aumentar a quantidade de alimentos ingerida, além de normalizar o apetite e a aceitação alimentar, para alcançar as necessidades nutricionais ou chegar o mais próximo possível delas.^(124)^

Podem ser tomadas algumas recomendações nutricionais antes que esses sintomas se apresentem, como^(124)^ aumentar o fracionamento das refeições; diminuir o volume da refeição, usando porções menores (por exemplo, *finger food*); substituir as refeições por lanches completos; modificar a consistência do alimento, se necessário; evitar preparações gordurosas ou muito ricas em molhos; melhorar a apresentação dos pratos, utilizando diversos utensílios, como caçarolas e pratos coloridos; realizar refeições em ambientes agradáveis; possibilitar a escolha das refeições conforme a melhor aceitação, em diferentes momentos do tratamento, como estratégia para aumentar o consumo; evitar a cobrança excessiva de ingestão alimentar; evitar o consumo abundante de bebidas, especialmente durante as refeições; aumentar a densidade calórica das preparações, utilizando suplementos nutricionais, se necessário e usar dieta enteral e/ou parenteral, caso precise, conforme a condição nutricional e clínica do paciente.

### Nefrotoxicidade de drogas

Complicações renais são muito frequentes e contribuem para a morbidade e a mortalidade relacionadas ao procedimento. Podem ter como causas a quimioterapia do condicionamento, a ICT, os agentes nefrotóxicos (por exemplo, inibidores da calcineurina), as infecções, a SOS hepática (anteriormente denominada doença veno-oclusiva – DVO – do fígado), a microangiopatia trombótica associada ao transplante e a DECH.^(128)^

Sabemos que a incidência da lesão renal aguda (LRA) é menos frequente no transplante autológo e mais prevalente principalmente no transplante alogênico mieloablativo. O início (dentro dos primeiros 30 dias do transplante) e a gravidade da piora renal estão associados ao aumento gradual do risco de morte e à diminuição da sobrevida global. Nos pacientes que requerem diálise, a mortalidade pode variar entre 55% a 100% dos casos.^(128)^ A incidência de doença renal crônica (DRC) varia amplamente, entre 7% a 48% dos casos, e pode desenvolver-se a partir de 6 meses até 10 anos após o transplante.^(129)^

Na avaliação inicial do paciente submetido ao TCTH e que apresentam sinais de sofrimento renal, deve-se tentar, inicialmente, estabelecer a causa dessa piora. É importante obter a análise de urina completa, a relação albumina-urina-creatinina urinária, um hemograma completo com avaliação do esfregaço de sangue e os níveis de lactato desidrogenase sérica, haptoglobina e inibidores da calcineurina. Deve-se realizar também pesquisa viral, principalmente do vírus BK e do adenovírus. Em casos individualizados, a biópsia renal pode ser ponderada, podendo identificar um possível DECH, a lesão endotelial e a inflamação crônica.^(128,129)^

Os agentes nefrotóxicos amplamente utilizados no transplante podem ser divididos em três grandes classes: os quimioterápicos (por exemplo, ciclofosfamida, carboplatina e fludarabina); os antimicrobianos, antivirais e antifúngicos (por exemplo, vancomicina, aciclovir e anfotericina) e os imunossupressores (por exemplo, inibidores de calcineurina, como a ciclosporina e o tacrolimus).^(128)^

Até 70% das crianças e adultos que receberam TCTH têm hipertensão durante os primeiros 2 anos após o procedimento. A hipertensão arterial sistêmica em crianças e adolescentes é definida como PA sistólica ou diastólica acima do percentil 95 para sexo, idade e altura, medida em, pelo menos, três ocasiões. Os fatores predisponentes incluem tratamento com ciclosporina, lesão renal aguda, TBI, obesidade e diabetes. A hipertensão tem se associado à maior probabilidade de desenvolvimento de DRC. Seu tratamento deve incluir modificações dietéticas e de estilo de vida, além de terapia medicamentosa, quando julgar necessário.^(129,130)^

Na instalação da LRA, não apenas o metabolismo hídrico, eletrolítico e ácido-básico é afetado, mas também há interferência no metabolismo de todos os macronutrientes, propiciando situações pró-inflamatórias, pró-oxidativas e de hipercatabolismo. Além das consequências relacionadas à própria LRA, somam-se as contribuições da doença de base e do TCTH e suas complicações, que podem interferir na depleção nutricional do paciente, caracterizando a síndrome *protein-energy wasting* (PEW).^(131)^

Entre as abordagens terapêuticas que visam retardar a progressão da lesão renal, está a redução de ingestão proteica, levando principalmente ao controle da taxa de filtração glomerular (TFG). A perda progressiva da função renal leva à DRC, classificada em estágios de 1 a 5 pela alteração da TFG, determinando o início de terapia dialítica.^(132)^

As quantidades recomendadas de proteínas seguem as recomendações do *The National Kidney Foundation and Kidney Disease Outcomes Quality Initiative* (NFK KDOQUI)™, conforme tabela 19.^(132)^

**Tabela 19:** Recomendação de proteínas nos estágios 3 a 5 na doença renal crônica

Idade	DRI (g/kg/dia)	Recomendação DRC 3 (g/kg/dia) 100%-140% DRI	Recomendação DRC 4 a 5 (g/kg/dia) 100%-120% DRI
0-6 meses	1,5	1,5-2,1	1,5-1,8
7-12 meses	1,2	1,2-1,7	1,2-1,5
1-3 anos	1,05	1,05-1,5	1,05-1,25
4-13 anos	0,95	0,95-1,35	0,95-1,15
14-18 anos	0,85	0,85-1,2	0,85-1,05

Fonte: Takakura CY, Murakami DK. Nefropatias. In: da Silva AP, Nascimento AG, Zamberlan P. Manual de dietas e condutas nutricionais em pediatria. 1a ed. São Paulo: Atheneu; 2014. p. 383-92.^(132)^

DRI: *dietary reference intakes*; DRC: doença renal crônica.

Quando há necessidade de terapia dialítica, seja ela hemodiálise ou diálise peritonial, o aporte proteico deve se manter em 100% da DRI para o peso ideal mais a quantidade referente às perdas de proteínas e aminoácidos no processo (Tabela 20).^(132)^

**Tabela 20:** Recomendação de proteínas na terapia dialítica

Idade	Hemodiálise (g/kg/dia)DRI + 0,1g/kg/dia	Diálise peritonial (g/kg/dia) DRI + 0,15 a 0,3g/kg/dia
0-6 meses	1,6	1,8
7-12 meses	1,3	1,5
1-3 anos	1,15	1,3
4-13 anos	1,05	1,1
14-18 anos	0,95	1,0

Fonte: Takakura CY, Murakami DK. Nefropatias. In: da Silva AP, Nascimento AG, Zamberlan P. Manual de dietas e condutas nutricionais em pediatria. São Paulo: Atheneu; 2014. p. 383-92.^(132)^

DRI: *Dietary reference intakes*.

Carboidratos, lipídios, sódio, potássio, cálcio, fósforo e líquidos variam de acordo com o nível de alteração da função renal e seguem as recomendações conforme as tabelas 21 e 22.^(132)^

**Tabela 21:** Recomendação de macronutrientes

Macronutriente	1-3 anos % do valor energético total	4-18 anos % do valor energético total
Carboidrato	45-65	45-65
Lipídio	30-40	25-35
Proteina	5-20	10-30

Fonte: Dalle JH, Giralt SA. Hepatic veno-occlusive disease after hematopoietic stem cell transplantation: risk factors and stratification, prophylaxis, and treatment. Biol Blood Marrow Transplant. 2016;22(3):400-9. Review.^(133)^

**Tabela 22:** Recomendação de micronutrientes e líquidos

Idade	Sódio (mg/dia)	Potássio (mg/dia)	Cálcio (mg/dia)	Fósforo (mg/dia)	Líquidos (mL/dia)	Vitaminas e demais minerais
0-6 meses	120	40-120	100%-200% DRI (ingestão diária + medicação)	Hiperfofatemia 80% das DRI	Perdas insensíveis conforme idade + função renal residual + outras perdas	100% DRI se comprovada deficiência, suplementação específica
7-12 meses	370					
1-3 anos	1.000					
4-13 anos	1.200 - 1.500					
14-18 anos	1.500					

Fonte: Takakura CY, Murakami DK. Nefropatias. In: da Silva AP, Nascimento AG, Zamberlan P. Manual de dietas e condutas nutricionais em pediatria. São Paulo: Atheneu; 2014. p. 383-92.^(132)^

DRI: *dietary reference intakes.*

### Síndrome de obstrução sinusoidal

A SOS, anteriormente chamada de DVO, pode ser uma grave complicação após o TCTH. Ela foi inicialmente descrita em pacientes que ingeriram chá contendo alcaloides de pirrolizidina. Embora possa ocorrer em vários outros cenários, como após irradiação hepática, exposição a quimioterápicos hepatotóxicos, uso de azatioprina, ou, mais recentemente, gemtuzumabe ozogamicina, a SOS é mais comumente vista no contexto da quimioterapia em altas doses com TCTH, tendo sido descrita pela primeira vez em 1979.^(133)^

Ela geralmente se desenvolve nos primeiros 30 dias após o TCTH, embora possa ocorrer mais tarde. Historicamente, sua incidência varia de aproximadamente 5% a 60% em adultos, e essa variação não está relacionada apenas com a intensidade do regime de condicionamento, o tipo de transplante e a presença de fatores de risco, mas também com os critérios clínicos utilizados para o diagnóstico. De maneira geral, a frequência e a gravidade da SOS têm sido reduzidas nos últimos anos, em decorrência de mudanças nos regimes preparatórios. A severidade varia bastante, indo desde formas leves, que se resolvem dentro de poucas semanas, a uma síndrome grave, definida pela presença de insuficiência de múltiplos órgãos e associada à alta taxa de mortalidade.^(134)^

A incidência da SOS em pacientes pediátricos submetidos ao TCTH varia de 11% a 31%, com taxa de mortalidade associada de até 50%.^(135)^

A SOS ocorre devido ao dano causado pelo regime de condicionamento às células endoteliais dos sinusoides hepáticos e nos hepatócitos, com deposição de fibrinogênio, fator VIII e congestão de eritrócitos, resultando em alargamento dos sinusoides e oclusão, podendo progredir para alteração da arquitetura hepática normal e necrose centrolobular. Nas fases tardias da doença, ocorrem fibrose e oclusão das vênulas terminais, levando à insuficiência hepática e, possivelmente, à morte. Paralelo ao dano físico, existe um estado pró-coagulante com níveis aumentados de inibidor do ativador do plasminogênio 1 e baixos níveis de antitrombina, proteína C e fator VII.^(136)^

A compreensão dos fatores de risco associados ao desenvolvimento da SOS é importante para o início precoce do tratamento ou profilaxia. Em geral, os fatores de risco podem ser divididos em duas categorias: características do paciente pré-transplante e fatores relacionados ao transplante. Os principais fatores de risco relacionados ao transplante relacionados para a SOS são^(134)^ TCTH alo, doador não aparentado, doador HLA-incompatível, regime de condicionamento mieloablativo, regime de condicionamento baseado em bussulfano ou TBI, enxerto sem depleção de células T e segundo TCTH. Já os fatores relacionados ao paciente são os seguintes: pacientes mais velhos (dentro da população adulta); mulheres em uso de noretisterona; pontuação de Karnofsky abaixo de 90%; polimorfismo genético (GSTM1, GSMTT1 e heparanase); doenças avançadas (após a segunda remissão ou recidiva); síndromes metabólicas; deficiência de antitrombina III e fator ativador de plasminogênio; resistência à proteína C ativada e talassemia. Em relação ao fígado, os fatores são transaminases > 2,5 vezes acima do limite superior da normalidade; bilirrubina sérica > 1,5 vez acima do limite superior da normalidade; cirrose; fibrose hepática; hepatite viral ativa; irradiação hepática; uso prévio de gemtuzumabe ozogamicina; uso de drogas hepatotóxicas e sobrecarga de ferro. Dentre os fatores específicos da população pediátrica, estão: linfo-histiocitose hemofagocítica, adrenoleucodistrofia e osteopetrose; TCTH autólogo com altas doses de quimioterapia em neuroblastoma; baixa idade (inferior a 1 a 2 anos); baixo peso e leucemia mielomonocítica juvenil.

A SOS é clinicamente caracterizada por retenção de líquidos e ascite, icterícia, ganho de peso e hepatomegalia dolorosa, na ausência de outras causas identificáveis de doença hepática. Existem dois critérios clínicos clássicos para a definição diagnóstica da SOS. Um deles é o critério de Baltimore, segundo o qual, nos primeiros 21 dias após o TCTH, o paciente deve apresentar bilirrubinas ≥2mg/dL e mais dois dos seguintes achados: hepatomegalia dolorosa, ascite e ganho de peso (≥5% do peso basal). O outro critério é o de Seattle, que prediz, nos primeiros 20 dias após o TCTH, dois ou mais dos seguintes achados: bilirrubinas ≥2mg/dL, hepatomegalia ou dor no quadrante superior direito do abdome e ganho de peso (≥2% do peso basal).

A tríade formada por ganho de peso, hepatomegalia dolorosa e bilirrubina elevada é variável em casos menos graves e pode ser incompleta ou atrasada em pacientes pediátricos, em comparação com adultos. Esses critérios clínicos podem ser complementados por exames não invasivos, como a ultrassonografia, para identificar ascite, hepatomegalia, espessamento da parede da vesícula biliar e fluxo venoso hepático atenuado ou reverso. O uso de testes invasivos (por exemplo, biópsia hepática percutânea ou transjugular) deve ser pesado contra o risco de sangramento associado a tais procedimentos. Além da hiperbilirrubinemia, outros achados laboratoriais associados com SOS incluem elevação de transaminases, tempo prolongado de protrombina e sinais de diminuição da função sintética (por exemplo, albumina baixa).^(133)^

Embora a icterícia esteja geralmente presente em adultos, pode estar ausente na SOS que se desenvolve tardiamente após o TCTH e, muitas vezes, está ausente em crianças. Dessa forma, não deve ser um pré-requisito para o diagnóstico da SOS.^(137,138)^

A SOS tem sido classificada retrospectivamente como leve, moderada ou grave, com base na gravidade da doença, incluindo o grau de disfunção hepática e a necessidade de terapia. Entretanto, esses critérios são vagamente definidos e não podem ser usados para prever o risco ou orientar o tratamento.^(133)^

Considerando-se a alta taxa de mortalidade associada à SOS severa, o monitoramento diário rigoroso para detectar precocemente os sinais e sintomas da síndrome deve ser realizado desde o início do condicionamento. Devem-se monitorar diariamente ganho de peso, retenção de líquidos, edema manifesto e ascite, hepatomegalia e icterícia. Os pacientes que apresentarem um desses fatores de risco necessitam de maior atenção.

Outros achados podem estar presentes, como sintomas relacionados à retenção de líquidos (derrame pleural, infiltrado pulmonar e hipóxia). O início precoce de plaquetopenia refratária à transfusão, não explicado por condições concomitantes, como sepse, pode ser um sinal inicial de SOS, refletindo a natureza endotelial da fisiopatologia da síndrome. A presença de disfunção renal ou pulmonar (ou, menos frequentemente, disfunção do sistema nervoso central) define uma insuficiência multiorgânica e a SOS grave.^(134)^

A adoção de medidas preventivas que possam reduzir a incidência e/ou a severidade da SOS é indispensável, especialmente porque não existem medidas terapêuticas com 100% de eficácia para essa patologia. As medidas preventivas combinam duas abordagens: reversão dos fatores de risco e prevenção farmacológica. O uso de heparina ainda é bastante controverso. Os dados sobre a utilidade do ácido ursodesoxicólico não são conclusivos: alguns ensaios randomizados sugerem que seu uso diminui a incidência de SOS, enquanto outros não demonstraram tal vantagem. No entanto, pacientes que receberam essa profilaxia tiveram menor toxicidade hepática, menos DECHa e melhor sobrevida, sugerindo fortemente um efeito benéfico. Além disso, tem sido demonstrado que o uso do ácido ursodesoxicólico está associado à diminuição da mortalidade não relacionada à recidiva.^(134)^

Considerando-se especificamente a população pediátrica, várias estratégias para a prevenção de SOS vêm sendo investigadas, incluindo lipoprostaglandina E1, profilaxia com danaparoide e um regime profilático combinado de heparina, glutamina e ursodiol. Estudo coreano retrospectivo com 374 crianças submetidas ao TCTH demonstrou que a profilaxia com lipoprostaglandina E1 pode ter efeito protetor contra SOS.^(136)^ Já revisão retrospectiva de 188 crianças que receberam um regime profilático combinado de heparina endovenosa, glutamina oral e ursodiol antes do TCTH demonstrou baixa incidência de SOS com essa abordagem (um caso em 188 pacientes).^(139)^ Muscaritoli et al.^(73)^ relataram benefícios da glutamina administrada durante o TCTH como possível protetora da função hepática para SOS. Entretanto, estudos prospectivos são necessários para melhor avaliação do impacto dessas estratégias. Finalmente, estudo prospectivo, randomizado, fase III, recentemente demonstrou incidência reduzida de SOS em pacientes pediátricos submetidos ao TCTH que receberam profilaxia com defibrotide.^(140)^ Alguns outros estudos que investigaram o uso de defibrotide profilático em criança também relataram incidência reduzida de SOS, bem como perfil de toxicidade favorável.^(141)^

O primeiro passo no tratamento da SOS é sintomático. Considerando se tratar de uma doença potencialmente fatal, a terapia deve ser iniciada o mais rapidamente possível. O manuseio do equilíbrio salino e hídrico e o uso cuidadoso de diuréticos devem ser introduzidos na primeira suspeita, quando a SOS ainda é apenas provável. O objetivo dos cuidados de suporte deve ser minimizar a sobrecarga de fluido extracelular, sem agravamento da função renal. Medidas sintomáticas podem ser usadas para reduzir o desconforto produzido por ascite maciça ou derrames pleurais. Particularmente em lactentes, quando a ascite maciça ameaça a respiração por meio de restrição pulmonar, a paracentese precoce pode ser extremamente útil para evitar complicações associadas com ventilação mecânica. Quando o acúmulo de líquido e a insuficiência renal não podem ser controlados, a hemodiálise/hemofiltração é necessária. O tratamento da SOS grave requer transferência para unidade de terapia intensiva.^(133,134)^

Vários estudos clínicos têm demonstrado a eficácia do defibrotide para o tratamento da SOS. Na Europa, ele está indicado para o tratamento da SOS grave em adultos, adolescentes e crianças com idade superior a 1 mês. Nos Estados Unidos, defibrotide foi recentemente aprovado pela *US Food and Drug Administration* para tratamento de pacientes adultos e pediátricos com SOS com disfunção renal ou pulmonar após TCTH.^(141,142)^

Quando instalada a SOS, ocorrem alterações no estado nutricional, caracterizadas pela redução do anabolismo. A principal consequência é o ganho de peso decorrente da retenção renal de sódio e água. Nesse caso, a terapia nutricional tem como objetivo favorecer a reversão dos fluidos intravasculares e a perda de eletrólitos.^(143,144)^ Deve-se ter em mente que, no período de maior incidência para ocorrência de SOS, o ganho de peso deve ser avaliado com cautela, pois ele se mostra um dos sinais iniciais para diagnóstico da SOS.

Não há recomendação nutricional específica para a SOS. Alguns autores citavam a restrição da dieta a aminoácidos de cadeia ramificada com a finalidade de se evitar a progressão para a encefalopatia hepática.^(73,145)^ Porém não foram encontrados estudos que testaram tal restrição nesses pacientes, assegurando sua indicação.

No ano de 2009, a *European Society for Clinical Nutrition and Metabolism* (ESPEN)^(146)^ recomendava a restrição da dieta a aminoácidos de cadeia ramificada apenas nos casos de encefalopatia grave, nos graus 3 e 4. Na última publicação da sociedade americana, foi recomendada somente nos casos de encefalopatia refratária em pacientes críticos.^(147)^

Desta forma, sugere-se que seja realizada a terapia nutricional de acordo com as recomendações gerais do tratamento do TCTH.

### Complicações endócrinas e metabólicas

Complicações endócrinas e metabólicas podem ocorrer em crianças, mesmo naquelas submetidas a condicionamento de intensidade reduzida e sem radioterapia. Isso porque o sistema endócrino é extremamente suscetível a danos pelo regime de condicionamento. As complicações endócrinas mais comuns são o hipotireoidismo, a falência gonadal, a redução na densidade mineral óssea e a baixa estatura devido à deficiência no hormônio do crescimento ou hipopituitarismo. Esse é um assunto que vem sendo cada vez mais explorado na literatura científica e constitui preocupação importante, principalmente em crianças e adolescentes pré-púberes, que ainda se encontram em fase de crescimento e desenvolvimento.^(148)^

Prejuízos no crescimento em crianças submetidas ao TCTH ocorrem devido a uma complexidade de fatores.^(149)^ Estudo realizado com 181 pacientes submetidos ao TCTH durante a infância e que atingiram sua estatura final mostrou que 80% deles atingiram altura dentro dos parâmetros de normalidade. Maior comprometimento foi observado em meninos do que em meninas, nas crianças transplantadas com menor idade ou que receberam TBI.^(10)^ A TBI também leva a alterações na função tiroidiana. O hipotireoidismo pode ser uma complicação do pós-TCTH imediato e também tardia, identificada anos após o procedimento. Aproximadamente 15% dos pacientes desenvolvem hipotireoidismo primário, que deve ser adequadamente tratado, evitando maiores repercussões clínicas.^(10)^

Reduções na densidade mineral óssea são comuns tanto alguns meses após o TCTH como também após vários anos (mais de 10), sendo a prevalência de osteopenia e osteoporose nessa população entre 24% e 57%. Presença de DECHc, uso de corticoides e baixos níveis séricos de vitamina D são fatores de risco para redução na densidade mineral óssea em crianças pós-TCTH.^(17)^ Vários estudos têm demonstrado a alta prevalência de deficiência de vitamina D, alertando para a associação entre baixos níveis dessa vitamina e presença ou progressão da DECH, uma vez que a vitamina D pode estar envolvida no controle da resposta imune, atuando na inibição da proliferação de células T e na produção de citocinas, ficando recomendadas a dosagem e a suplementação de vitamina D antes e após o TCTH,^(16,150)^ conforme DRI. A avaliação do consumo alimentar de cálcio é importante, particularmente nos pacientes com DECH ou sob uso de corticoides, sendo a recomendação de ingestão desse mineral aumentada. A suplementação deve ser prescrita para complementar a ingestão alimentar quando ela não atingir as recomendações da *Recommended Dietary Allowance* (RDA) (Tabela 23). O consumo de cálcio além dessas recomendações pode ser prejudicial devido à interferência na absorção de outros nutrientes.

**Tabela 23:** Recomendações para ingestão diária de cálcio e vitamina D

Idade (anos)	Ingestão diária recomendada		Ingestão diária recomendada durante uso de corticoides	
	Vitamina D (UI)	Cálcio (mg)	Vitamina D (UI)	Cálcio (mg)
1-3	600	700	600-1.000	800
4-8	600	1.000	600-1.000	1.200
9-13	600	1.300	600-1.000	1.500
14-18	600	1.300	600-1.000	1.500

Fonte: Campos DJ, Boguszewski CL, Funke VA, Bonfim CM, Kulak CA, Pasquini R, et al. Bone mineral density, vitamin D, and nutritional status of children submitted to hematopoietic stem cell transplantation. Nutrition. 2014;30(6):654-9;^(16)^ Shalitin S, Phillip M, Stein J, Goshen Y, Carmi D, Yaniv I. Endocrine dysfunction and parameters of the metabolic syndrome after bone marrow transplantation during childhood and adolescence. Bone Marrow Transplant. 2006;37(12):1109-17.^(151)^

O diabetes tipo 2 é outra complicação que pode ocorrer após o TCTH, mas em menor prevalência do que as demais complicações endócrinas, sendo os fatores de risco história familiar de diabetes, raça não caucasiana e diagnóstico de leucemia.^(149,151)^ O uso de corticoides também pode induzir ao diabetes durante o período de uso dessa medicação.^(151)^ Crianças submetidas ao TCTH também estão em risco para o desenvolvimento de resistência à insulina e o acúmulo de gordura abdominal.^(152)^ A síndrome metabólica, que é caracterizada por hipertensão, dislipidemia, obesidade e alteração no metabolismo da glicose, leva ao aumento no risco de doença cardiovascular precoce e diabetes tipo 2, estando presente em 7% a 32% das crianças submetidas ao TCTH.^(58,153,154)^

Como monitoramento e prevenção das alterações endócrinas e metabólicas, o consenso internacional em efeitos tardios após o TCTH em pediatria recomenda^(131)^ avaliar: função tiroidiana anualmente; velocidade de crescimento anualmente, com acréscimo de determinação da idade óssea naqueles com crescimento abaixo do ideal; densidade mineral óssea por densitometria corporal antes do TCTH, com repetição após 1 e 5 anos, com exceção nos pacientes com reduzida densidade mineral óssea, que devem ser avaliados anualmente e perfil lipídico e glicemia de jejum pelo menos a cada 5 anos e anualmente quando existirem alterações. Também deve ser realizado o monitoramento dos níveis séricos de cálcio, magnésio e vitamina D (25OHD) após o TCTH, principalmente nos pacientes com alterações na densidade mineral óssea. Os pacientes devem ser orientados a seguir uma alimentação saudável, com consumo adequado de cálcio e vitamina D. Também devem ser alertados sobre os efeitos negativos do fumo, do consumo de bebidas alcóolicas e da cafeína e estimulados à prática de atividades físicas.

No pós-TCTH, é recomendado seguir uma alimentação saudável, objetivando não somente a prevenção das complicações endócrinas e metabólicas, mas também das demais doenças crônicas e dos cânceres. Toda a família do paciente transplantado deve ser estimulada a seguir um padrão alimentar adequado, para que a compreensão e a aderência pelo paciente sejam otimizadas, promovendo a educação em saúde e a prevenção de doenças em toda a família. Dois dos principais pontos a serem trabalhados são: promover adequado consumo de frutas, verduras e legumes, e restringir consumo de alimentos processados. O nutricionista deve basear essas orientações nos materiais do Ministério da Saúde.^(155,156)^

### Disgeusia

A disgeusia é definida como a distorção ou diminuição persistente do paladar.^(157,158)^ Vários mecanismos podem estar envolvidos na causa dos distúrbios de paladar e olfato frequentemente observados em pacientes portadores de doenças onco-hematológicas, como alterações na atividade neuronal (sensibilização anormal de alguns ramos do nervo facial), desequilíbrio nos níveis de oxidantes e antioxidantes (devido à peroxidação de células epiteliais da cavidade oral) e destruição direta das células dos receptores olfativos e gustativos.^(158)^

Os protocolos de quimioterapia utilizam drogas que interferem na atividade mitótica, com o objetivo de destruir células de rápida proliferação (células cancerígenas). Como as células das papilas gustativas apresentam rápida proliferação, a renovação dessas células também é interrompida durante o período de tratamento.^(159)^ Pode ocorrer também dano no epitélio da mucosa oral causada pela ação direta da radiação sobre as papilas gustativas, no caso de pacientes submetidos à radioterapia em região de cabeça e pescoço.^(160,161)^ Estima-se que essas alterações, variem de 40 a 75%.^(157,158,162–167)^

A disgeusia encontra-se frequentemente associada a alterações olfatória, já que os dois sentidos estão intimamente envolvidos na obtenção da sensação de sabor.^(168)^ Vários estudos demonstram associação entre disgeusia e redução da ingesta oral, aumento no tempo de nutrição parenteral, perda de peso significativa e redução da capacidade funcional dos pacientes durante o tratamento,^(169–171)^ apresentando impacto negativo na qualidade de vida do paciente e sendo uma das principais causas de recusa alimentar em crianças.^(161,172,173)^

No contexto do TCTH pediátrico, alguns tipos de condicionamento, como os que utilizam altas doses de melfalan, e a presença de mucosite oral são fatores de risco independentes para disgeusia, enquanto o uso da crioterapia parece ser fator independente protetor.^(172)^ A crioterapia oral leva à vasoconstricção e à diminuição do fluxo sanguíneo na cavidade oral, reduzindo a exposição da mucosa oral ao quimioterápico e diminuindo a incidência de mucosite oral.^(174–176)^ O procedimento consiste em chupar pequenos cubos de gelo 30 minutos antes, durante os 60 minutos de infusão do quimioterápico (exemplo: melfalan) e 30 minutos após término da infusão.^(177)^

Diversos tratamentos medicamentosos têm sido descritos, porém ainda sem eficácia comprovada:^(157,178,179)^

- Suplementação com zinco (cofator da fosfatase alcalina): demonstrou eficácia em melhorar a função gustatória e os escores de qualidade de vida em geral em pacientes com disgeusia idiopática.^(157,168)^ Estudos realizados posteriormente em pacientes portadores de câncer de cabeça e pescoço submetidos à radioterapia e/ou quimioterapia demonstraram discreta melhora nos sintomas de disgeusia de pacientes submetidos à quimioterapia e/ou radioterapia.^(157,179,180)^
- Amifostine (agente citoprotetor): apesar do efeito comprovado na diminuição da incidência e gravidade da toxicidade pela quimioterapia e/ou radioterapia devido ao seu efeito antioxidante, estudos não comprovaram benefício na prevenção e/ou tratamento da disgeusia.^(158,181,182)^
- Orientação nutricional individualizada: por meio de orientação oral direta, folhetos e/ou audioaulas, parece ter efeito benéfico na diminuição dos sintomas, apresentando melhor efeito na disgeusia de longa duração do que na de início precoce. As principais orientações estão descritivas na tabela 24.^(157,183,184)^

**Tabela 24:** Resumo da orientação nutricional para diminuir os sintomas de disgeusia

Orientações nutricionais para disgeusia
Conscientizar o paciente da necessidade de comer apesar dos sintomas de disgeusia e/ou disosmia
Estimular a ingesta de alimentos mais prazerosos
Modificar a consistência dos alimentos conforme aceitação
Aumentar o fracionamento da dieta (6 a 8 refeições ao dia)
Avaliar associação de suplemento oral hipercalórico/hiperproteico (2 a 3 vezes ao dia)
Orientar o paciente a preparar pratos visualmente agradáveis, dando preferência a alimentos de sabor mais forte, utilizar gotas de limão nas saladas e bebidas, ervas aromáticas e condimentos nas preparações para realçar o sabor e se lembrar do sabor dos alimentos antes de ingeri-los

Fonte: Brasil. Ministério da Saúde. Instituto Nacional de Câncer José de Alencar Gomes da Silva (INCA). Consenso nacional de nutrição oncológica. 2a. ed. rev. ampl. atual. Rio de Janeiro: INCA; 2015. p.182 [citado 2020 Jul 4]. Disponível em: https://www.inca.gov.br/sites/ufu.sti.inca.local/files/media/document/consenso-nacional-de-nutricao-oncologica-2-edicao-2015.pdf.^(20)^

Também foram citados como adjuvantes na melhora dos sintomas o uso de suco de limão, a ingesta de balas e sucos doces antes das refeições, o uso de utensílios de plástico e canudo e bochechos com sal ou enxaguantes bucais, embora com pouca evidência científica.^(185)^ Alguns estudos sugerem que o uso de temperos como sal, glutamato de sódio ou potássio e açúcar possam ser benéficos no manejo da disgeusia.^(186)^ A acupuntura não demonstrou benefícios.^(187)^

Outras terapias empíricas já foram sugeridas, porém sem benefício comprovado em estudos, como o uso de corticoides, vitamina A, *Ginkgo biloba*, glutamina e gabapentina.^(188,189)^ Novas perspectivas incluem o uso de *Canabbis* (Dronabinol) e do ácido alfa lipóico.^(190–193)^

Alguns estudos, a maioria realizada em adultos, demonstraram que a disgeusia poderia durar de 1 a 3 anos após o término do tratamento. Recente estudo realizado em pacientes pediátricos submetidos ao TCTH demonstrou resolução do quadro de disgeusia em uma média de 6 meses após a enxertia. A discrepância nos resultados pode ser explicada pela maior velocidade de regeneração dos receptores em crianças do que em adultos.^(194)^

### Diarreia

A doença diarreica é uma das complicações gastrintestinais mais frequentes dos TCTH alogênicos, podendo ocorrer em outros tipos de transplantes.^(195)^ A doença diarreica ocorre geralmente entre o sexto e o 11º dia após transplante, mas pode estar presente em todas as fases do TCTH. Observou-se redução da quantidade de energia ingerida nesses períodos devido a náusea, vômito, diarreia e perda do apetite.^(196)^ A doença diarreica grave pode ser definida por apresentar mais de seis evacuações – segundo a escala de Bristol, seis ou sete.^(197)^ Outra definição de perdas graves fecais seria perda de volume maior que 30mL/kg, correspondendo às perdas fecais acarretadas pela cólera.^(198)^

As causas de doença diarreica no TCTH podem ser classificadas em quatro grandes grupos, conforme tabela 25.

**Tabela 25:** Causas da doença diarreica em transplante de células-tronco hematopoiéticas pediátrico

Causa	Mecanismo
Tratamento/ condicionamento	Antibióticos: azatioprina
	Inibidores de calcineurina: ciclosporina, metotrexato, tacrolimus, sirolimus e tiotepa
	Agentes alcalinizantes: ciclofosfamida, melfalano e bussulfano
	Irradiação corporal total
Infecciosas – vírus, bactérias, fungos e parasitas	Bactérias: *Clostridioides difficile, Campylobacter jejuni/coli*, *Listeria monocytogenes*, *Escherichia coli* 0157:H7, *Salmonella species*, *Shigella species*, *Vibrio species* ou *Yersinia species* e nocarioses
	Vírus: adenovírus, Rotavírus, citomegalovírus e noravírus
	Parasitas: giardíase, fusariose, *Microsporidium*, histoplasmose e *Candida species*
Síndrome da colite de cordão umbilical	Não possui associação com infecções ou doença do enxerto contra hospedeiro, caracterizando-se por uma doença granulomatosa que responde à antibioticoterapia (ciprofloxacina e metronidazol)
Doença enxerto contra hospedeiro	Uma das causas de doença diarreica no transplante alogênico, sendo acompanhada de icterícia, *rash* de pele e outras manifestações clínicas do trato gastrintestinal (dor abdominal e presença de íleo paralítico). Pode ocorrer após o 15^º^ dia do transplante, sendo um diagnóstico muito provável após terceira semana do transplante. Orienta-se sempre afastar outras causas de doenças diarreicas antes de iniciar drogas imunossupressoras

Fonte: Robak K, Zambonelli J, Bilinski J, Basak GW. Diarrhea after allogeneic stem cell transplantation: beyond graft-versus-host disease. Eur J Gastroenterol Hepatol. 2017;29(5):495-502. Review;^(199)^ Morishita T, Tsushita N, Imai K, Sakai T, Miyao K, Sakemura R, et al. The efficacy of an oral elemental diet in patients undergoing hematopoietic stem cell transplantation. Intern Med. 2016;55(24):3561-9;^(200)^ Goldberg RJ, Nakagawa T, Johnson RJ, Thurman JM. The role of endothelial cell injury in thrombotic microangiopathy. Am J Kidney Dis. 2010;56(6):1168-74;^(201)^ Shah NN, McClellan W, Flowers CR, Lonial S, Khoury H, Waller EK, et al. Evaluating risk factors for clostridium difficile infection in stem cell transplant recipients: a national study. Infect Control Hosp Epidemiol. 2017;38(6):651-7;^(202)^ Webb BJ, Brunner A, Ford CD, Gazdik MA, Petersen FB, Hoda D. Fecal microbiota transplantation for recurrent Clostridium difficile infection in hematopoietic stem cell transplant recipients. Transpl Infect Dis. 2016;18(4):628-33;^(203)^ Kwak KS, Zhou X, Solomon V, Baracos VE, Davis J, Bannon AW, et al. Regulation of protein catabolism by muscle-specific and cytokine-inducible ubiquitin ligase E3alpha-II during cancer cachexia. Cancer Res. 2004;64(22):8193-8;^(204)^ Lee JL, Leong LP, Lim SL. Nutrition intervention approaches to reduce malnutrition in oncology patients: a systematic review. Support Care Cancer. 2016;24(1):469-80. Review;^(205)^ Watson T, MacDonald D, Song X, Bromwich K, Campos J, Sande J, et al. Risk factors for molecular detection of adenovirus in pediatric hematopoietic stem cell transplantation recipients. Biol Blood Marrow Transplant. 2012;18(8):1227-34;^(206)^ Srinivasan A, Klepper C, Sunkara A, Kang G, Carr J, Gu Z, et al. Impact of adenoviral stool load on adenoviremia in pediatric hematopoietic stem cell transplant recipients. Pediatr Infect Dis J. 2015;34(6):562-5;^(207)^ César Pereira Santos H, Nunes Vieira Almeida T, Souza Fiaccadori F, das Dôres de Paula Cardoso D, de Moraes Arantes A, Delleon da Silva H, et al. Adenovirus infection among allogeneic stem cell transplant recipients. J Med Virol. 2017;89(2):298-303;^(208)^ Williams D. Treatment of rotavirus-associated diarrhea using enteral immunoglobulins for pediatric stem cell transplant patients. J Oncol Pharm Pract. 2015;21(3):238-40;^(209)^ Patel NC, Hertel PM, Hanson IC, Krance RA, Crawford SE, Estes M, et al. Chronic rotavirus infection in an infant with severe combined immunodeficiency: successful treatment by hematopoietic stem cell transplantation. Clin Immunol. 2012;142(3):399-401;^(210)^ Liu A, Meyer E, Johnston L, Brown J, Gerson LB. Prevalence of graft versus host disease and cytomegalovirus infection in patients post-haematopoietic cell transplantation presenting with gastrointestinal symptoms. Aliment Pharmacol Ther. 2013;38(8):955-66;^(211)^ Ye X, Van JN, Munoz FM, Revell PA, Kozinetz CA, Krance RA, et al. Noroviruses as a cause of diarrhea in immunocompromised pediatric hematopoietic stem cell and solid organ transplant recipients. Am J Transplant. 2015;15(7):1874-81;^(212)^ Ueda R, Fuji S, Mori S, Hiramoto N, Hashimoto H, Tanaka T, et al. Characteristics and outcomes of patients diagnosed with norovirus gastroenteritis after allogeneic hematopoietic stem cell transplantation based on immunochromatography. Int J Hematol. 2015;102(1):121-8;^(213)^ Troeger H, Loddenkemper C, Schneider T, Schreier E, Epple HJ, Zeitz M, et al. Structural and functional changes of the duodenum in human norovirus infection. Gut. 2009;58(8):1070-7;^(214)^ Saif MA, Bonney DK, Bigger B, Forsythe L, Williams N, Page J, et al. Chronic norovirus infection in pediatric hematopoietic stem cell transplant recipients: a cause of prolonged intestinal failure requiring intensive nutritional support. Pediatr Transplant. 2011;15(5):505-9;^(215)^ Ajumobi AB, Daniels JA, Sostre CF, Trevino HH. Giardiasis in a hematopoietic stem cell transplant patient. Transpl Infect Dis. 2014;16(6):984-7;^(216)^ Bouanani N, Lamchahab M, Quachouh M, Soussi M, Quessar A, Benchekroun S. [Disseminated fusariosis during autologous stem cells transplant]. J Mycol Med. 2013;23(2):119-22. French;^(217)^ Krones E, Högenauer C. Diarrhea in the immunocompromised patient. Gastroenterol Clin North Am. 2012;41(3):677-701. Review;^(218)^ Milano F, Shulman HM, Guthrie KA, Riffkin I, McDonald GB, Delaney C. Late-onset colitis after cord blood transplantation is consistent with graft-versus-host disease: results of a blinded histopathological review. Biol Blood Marrow Transplant. 2014;20(7):1008-13;^(219)^ Gupta NK, Masia R. Cord colitis syndrome: a cause of granulomatous inflammation in the upper and lower gastrointestinal tract. Am J Surg Pathol. 2013;37(7):1199-13.^(220)^ Ball LM, Egeler RM; EBMT Paediatric Working Party. Acute GvHD: pathogenesis and classification. Bone Marrow Transplant. 2008;41(Suppl 2):S58-64. Review.^(221)^Kambham N, Higgins JP, Sundram U, Troxell ML. Hematopoietic stem cell transplantation: graft versus host disease and pathology of gastrointestinal tract, liver, and lung. Adv Anat Pathol. 2014;21(5):301-20. Review. ^(222)^

O comprometimento das estruturas intestinais, a disfunção de órgãos responsáveis pela digestão dos nutrientes e o uso de medicações modificando a microbiota intestinal corroboram para a gênese da doença diarreica. A lesão das vilosidades intestinais favorece o comprometimento da atividade da lactase, que acarreta intolerância à lactose em diversos graus. Além disso, a ruptura da barreira intestinal facilita a exposição de macroproteínas, que pode gerar alergias múltiplas, como ao leite de vaca, à soja e ao glúten.^(199)^

A etiologia da doença diarreia devido a agentes infecciosos deve ser sempre afastada, pois é muito frequente em pacientes imunossuprimidos e no TCTH.

Um algoritmo para avaliar as causas de doença diarreica nos TCTH alogênicos foi proposto por Robak K et al.^(199)^ Deve-se primeiro afastar as causas infecciosas; depois, afastar DECH e, por último, as outras causas.

Nos pacientes com aceitação da dieta via oral, que alcance 70-80% das necessidades energética e de micronutriente, recomendam-se monitorar a aceitação e orientar a suplementação alimentar.^(223)^ A dieta ofertada deve ter pouco resíduos. Além disso, deve-se orientar o fracionamento de refeições, oferecendo de seis a oito porções por dia, e garantir uma hidratação adequada para a faixa etária. A aceitação de alguns alimentos irritantes ao TGI, como frituras ou doces, e de nutrientes específicos, como lactose, sacarose, glúten e cafeína, deve ser avaliada individualmente, e, se necessário, a ingestão pode ser restrita ao aparecimento da diarreia.^(124)^

A nutrição enteral, com utilização de sondas nasogástricas ou pós-pilóricas, torna-se necessária nos pacientes que não ingerem por via oral 70% a 80% das necessidades de energia e micronutrientes com comprometimento nutricional, avaliando a administração da dieta e o tipo de proteína utilizada (dieta polimérica ou extensivamente hidrolisada). Deve-se proceder à passagem da sonda no início do condicionamento até a primeira semana pós-transplante, quando a ingestão oral se torna comprometida. A infusão de dieta enteral deve ser sempre realizada por meio de uma bomba de infusão, que tem como objetivo controlar o volume infundido.^(224)^ Se não houver progressão do volume da nutrição enteral suficiente para atingir 70% da taxa metabólica basal ou 3 dias de jejum, considera-se a indicação de nutrição parenteral.

### Intervenção nutricional

De acordo com as diretrizes para a terapia nutricional durante o TCTH, todos os pacientes submetidos a esse procedimento com regimes de condicionamento mieloablativo são de risco nutricional.^(76,95,225)^

É importante o acompanhamento nutricional em todas as fases do tratamento, sendo possível identificar adequadamente o risco nutricional e implementar terapia nutricional o mais precocemente possível, de acordo com as condições e as necessidades da criança e do adolescente, dentro de critérios que assegurem a melhor decisão, aumentando os benefícios desse suporte e evitando os riscos de uma indicação inadequada.^(76,95,225)^

O objetivo da terapia nutricional é, de forma geral, melhorar a resposta ao tratamento, diminuir os riscos de complicações e otimizar a sobrevida e a qualidade de vida.

As metas da terapia nutricional podem ser separadas de acordo com as fases do tratamento, conforme o tabela 26.

**Tabela 26:** Metas nutricionais de acordo com as fases do tratamento

Fase do tratamento	Meta nutricional
Pré-TCTH	Corrigir ou manter o estado nutricional adequado, corrigindo as reservas de macro e de micronutrientes para melhorar a tolerância ao tratamento
	Diminuir riscos de infecção
	Melhorar a imunidade e a resposta à inflamação durante o tratamento
Internação para a realização do TCTH (condicionamento, infusão celular e pega medular)	Minimizar o agravo nutricional
	Controlar os sintomas gastrintestinais
	Melhorar a resposta ao tratamento
	Diminuir complicações gerais: atenção especial ao ganho de peso que pode indicar complicações, como SOS ou nefrotoxicidade
	Minimizar o *deficit* de crescimento e de desenvolvimento
	Controlar a resposta pró-inflamatória
	Abreviar o tempo de internação
Pós-TCTH	Manter a curva adequada de crescimento e de desenvolvimento
	Corrigir o estado nutricional
	Controlar as repercussões nutricionais e metabólicas

Fonte: Garófolo A, Lopez FA, Petrilli AS. High prevalence of malnutrition among patients with solid non-hematological tumors as found by using skinfold and circumference measurements. Sao Paulo Med J. 2005;123(6):277-81;^(42)^ Papadopoulou A, Williams MD, Darbyshire PJ, Booth IW. Nutritional support in children undergoing bone marrow transplantation. Clin Nutr. 1998;17(2):57-63;^(69)^ Christensen ML, Hancock ML, Gattuso J, Hurwitz CA, Smith C, McCormick J, et al. Parenteral nutrition associated with increased infection rate in children with cancer. Cancer. 1993;72(9):2732-8;^(226)^ Dioguardi J, Bryson E, Ahmed-Winston S, Vaughn G, Slater S, Driscoll J, et al. A multi-institutional retrospective study suggests that optimal enteral nutrition (EN) influences outcomes after hematopoietic stem cell transplantation in children and adults. Biol Blood Marrow Transplant. 2015;21(2):S248-9;^(227)^ Bechard LJ, Guinan EC, Feldman HA, Tang V, Duggan C. Prognostic factors in the resumption of oral dietary intake after allogeneic hematopoietic stem cell transplantation (HSCT) in children. JPEN J Parenter Enteral Nutr. 2007;31(4):295-301;^(228)^ Carr SE, Halliday V. Investigating the use of the neutropenic diet: a survey of U.K. dietitians. J Hum Nutr Diet. 2015;28(5):510-5.^(229)^

TCTH: transplante de células-tronco hematopoiéticas; SOS: síndrome de obstrução sinusoidal.

### Terapia nutricional oral

A aceitação alimentar pode estar alterada pelos inúmeros efeitos colaterais do TCTH. Características da dieta ofertada devem seguir as necessidades do momento atual do paciente. Nesse contexto, dois tópicos relacionados à terapia nutricional oral merecem destaque: a dieta para neutropenia e a suplementação via oral.

### Dieta para neutropenia

Após o condicionamento para o TCTH, a imunidade é afetada, havendo um período de neutropenia com grande suscetibilidade a infecções. Historicamente, as restrições dietéticas conhecidas como dietas “limpas”, “baixas bactérias”, “microbianos” ou “neutropênicas”, têm sido prescritas para reduzir o risco de infecção alimentar.^(229)^

A neutropenia é definida como a contagem total de neutrófilos inferior a 500/mm^3^ ou expectativa de diminuição a níveis inferiores a esse nas 48 horas seguintes.^(230)^ O período de neutropenia varia segundo o tipo de TCTH e a presença ou não de complicações, como DECH e falha de pega medular.^(231)^

Após o regime preparativo, durante a fase de neutropenia, o paciente pode ter danos na mucosa de todo TGI, causados pelo condicionamento, que pode ser uma porta de entrada para patógenos *Gram*-negativos, tais como *Pseudomonas aeruginosa*, *Escherichia coli*, *Klebsiella* e *Proteus*, em diferentes alimentos. Quando os alimentos são cozidos, assados, fritos ou aquecidos, pode-se reduzir o número de microrganismos de forma considerável, sendo forte a recomendação para utilização de dietas com alimentos cozidos no momento da aplasia medular.^(232)^

A dieta para TCTH baseia-se na segurança alimentar e tem o objetivo de evitar infecções de origem alimentar e translocação bacteriana, na fase de aplasia e de fragilidade medular. A orientação nutricional deve ser focada nas boas práticas de higiene e preparo dos alimentos, tanto no treinamento de funcionários que irão manipular as refeições do paciente durante a internação quanto na orientação para os cuidadores da criança após a alta. Além disso, alguns alimentos devem ficar de fora da dieta por apresentarem risco aumentado de conter patógenos.^(99)^

Os alimentos que apresentam maior risco para os pacientes são ovos crus, produtos lácteos não pasteurizados, frutas, verduras e legumes com higienização inadequada, entre outros (Tabela 27).^(99)^

**Tabela 27:** Orientações alimentares de dieta para transplante de células-tronco hematopoiéticas

Restringir consumo
Água e gelo de procedência duvidosa, não filtrada ou fervida
Carnes cruas ou malcozidas (bovina, suína, de frango e cordeiro)
Ovo cru ou malcozido, ou preparações que os contenham
Leite e produtos lácteos frescos não pasteurizados (queijo, manteiga e iogurte)
Alimentos contendo probióticos
Hortaliças cruas sem higienização adequada
Cereais crus
Mel caseiro cru isento do selo de inspeção federal
Conservas caseiras isentas do selo de inspeção federal (por exemplo, palmito em conserva, azeitonas em conserva etc.)
Tofu cru
Caldo de cana
Açaí *in natura*
Alimentos não processados prontos para consumo comprados em restaurantes, lanchonetes, padarias etc.

Consumir de acordo com as orientações de preparo e higienização
Frutas cruas de casca fina e/ou de difícil higienização (por exemplo: amora, morango, uva, cereja, jabuticaba e mirtilo)
Frutas cruas com casca média e grossa não íntegras ou sem higienização adequada (por exemplo, abacate, maçã, manga, mamão, caqui, melancia, abacaxi, melão, banana, mexerica, laranja, maracujá, limão, pera, goiaba e ameixa)
Frutas secas e oleaginosas cruas
Queijos que não passaram por processo de cocção
Embutidos e frios que não passaram por processo de cocção (por exemplo, salsicha, salame, presunto etc.)
Ervas e temperos secos que não passaram por processo de cocção

Estudo que realizou questionário com vários profissionais que utilizavam essa dieta verificou que existe uma variedade de alimentos restritos, havendo, às vezes contradições. Os alimentos mais comumente restritos foram os frutos do mar e o peixe.^(229)^

A dieta para TCTH, ou para neutropenia, inicia-se no condicionamento e se estende até a retirada dos imunossupressores, nos TCTH alogênicos, ou até a adequada contagem neutrofílica, nos TCTH autólogos.^(99)^ Alguns autores brasileiros recomendam que a dieta para neutropenia seja estendida até 100 dias após o TCTH alogênico ou 60 dias no caso do autólogo. No entanto, outros autores sugerem que, para o TCTH alogênico, a dieta deve continuar até o final de todas as terapêuticas imunossupressoras e, na TCTH autóloga, até 1 mês após a descontinuação dos corticosteroides ou 3 meses após ausência de lesões gastrintestinais.^(233)^ Esse momento de suspensão da dieta ainda não é um consenso entre os centros especializados.

As diferenças na prática podem ser atribuídas à falta de evidência robusta de alta qualidade.^(229)^ Talvez forma de preparo, acondicionamento e qualidade da matéria-prima sejam mais importantes do que o tipo de alimento restrito, adaptando a realidade do hospital e população atendida.

Dessa forma, o papel da dieta neutropênica no desenvolvimento de infecções no TCTH ainda é controverso.^(234)^ Existem poucos estudos, tanto com população pediátrica quanto com a adulta. Outro viés é que são poucos os estudos que retratem a realidade da nossa população. Dessa forma, são necessários estudos não somente de qualidade, mas que retratem a realidade da nossa população.

### Suplementação via oral (artesanal e industrializada)

A indicação da suplementação nutricional oral deve ser avaliada de forma individualizada e ocorrer quando a avaliação da ingestão alimentar for <70% a 80% das recomendações nutricionais por de 3 a 5 dias consecutivos, considerando o estado nutricional de risco; o tempo de expectativa de melhora da ingestão alimentar e aquele previsto para enxertia; o TGI e o condicionamento.^(235–237)^ Para tanto, sugere-se que sejam realizados cálculos de ingestão alimentar diariamente.

Os suplementos podem ser industrializados ou artesanais.

Para a decisão de iniciar a suplementação via oral, alguns critérios devem ser considerados: redução na ingestão alimentar, independentemente de outros indicadores; risco de desnutrição, que significa qualquer perda de peso ou desaceleração da curva de crescimento; redução de reservas adiposas ou de massa muscular; alterações gastrintestinais, independentemente de outros indicadores e paciente submetido ao TCTH, independentemente de outras condições.

Para a escolha de qual suplemento utilizar, também alguns critérios devem ser incluídos, tais como faixa etária; sabor, apresentação e quantidade; estado nutricional e catabólico (pode ser necessário suplemento normo ou hipercalórico e/ou proteico); TGI (pode haver necessidade de suplementos hidrolisados ou com outras alterações); condição metabólica (alterações de glicose ou lipídios séricos; inflamação sistêmica); comorbidades (alterações hepáticas, renal, pâncreas, entre outras) e situação socioeconômica.

Os suplementos industrializados podem auxiliar na terapia nutricional, mas o alto custo dificulta sua aquisição, limitando o uso generalizado. Além disso, não existem muitas opções de suplementos orais para a faixa etária pediátrica, o que leva à necessidade de desenvolver alternativas artesanais, com a utilização de preparações atrativas, com ingredientes que possam fornecer maior densidade energético-proteica e micronutrientes.

Desse modo, os suplementos artesanais, obtidos pela modulação artesanal de ingredientes dietéticos, podem ser uma opção menos onerosa que os industrializados. Apesar disso, existem algumas desvantagens, quando comparados os suplementos artesanais aos industrializados, como o menor controle microbiológico pela maior manipulação, a maior complexidade na preparação e dificuldade em garantir valor nutricional equiparável.^(238,239)^

Maia et al.^(240)^ demonstram que a suplementação oral, quer seja com suplemento industrializado ou artesanal, pode prevenir o agravo do estado nutricional, principalmente quando o paciente é assíduo às consultas nutricionais e tem compromisso com as orientações. Entretanto, a aplicação do industrializado parece propiciar uma resposta favorável em uma percentagem maior de pacientes.

Em geral, a presença de desnutrição mais grave requer outras medidas de terapia nutricional, pois pacientes nessas circunstâncias em TCTH têm mais dificuldade para atingir sua necessidade por via oral.^(238,239)^

Desse modo, o uso de suplementos orais pode ser uma alternativa viável para prevenir a perda nutricional, evitando-se graus acentuados de magreza e sarcopenia. Os suplementos industrializados garantem uma oferta homogênea de nutrientes e demonstram ser mais práticos para preparação na rotina. Porém, podem apresentar-se monótonos para crianças em relação ao sabor. A combinação de receitas artesanais com suplementos industrializados é uma estratégia importante na prevenção da desnutrição. Quando não há recursos para a utilização de industrializados, suplementos artesanais devem ser orientados.

Frequentemente, o consumo proteico pode estar prejudicado, o que tende a intensificar o catabolismo e a sarcopenia. As necessidades proteicas nesses pacientes são elevadas, sendo necessário o uso de suplementos proteicos industrializados ou de estratégias alimentares de suplementação caseira para alcançar o aporte necessário.

A prescrição inicial dessa terapia deve representar cerca de 45% a 50% das necessidades nutricionais do paciente para que possa haver contribuição nutricional. A programação de retirada do suplemento oral pode ser realizada quando a ingestão oral começar a subir ≥70% a 80% das necessidades nutricionais calculadas por 2 ou 3 dias, devendo atingir cerca 100% das necessidades sem o suplemento oral.^(240)^

### Nutrição enteral

Para a realização de TNE, deve-se avaliar a capacidade funcional do TGI. Situações que modifiquem os sistemas de digestão e absorção, como mucosite, infecções, DECH, entre outras, podem comprometer o adequado aproveitamento dos nutrientes e ser ineficientes, caso sejam mal indicadas.^(241,242)^

Com base nos conceitos de que o jejum prolongado causa atrofia da mucosa intestinal, rompendo a integridade imunológica do TGI e aumentando o risco de translocação bacteriana, o alimento constitui importante estímulo para manter a função e a estrutura da mucosa intestinal, liberando secreções pancreáticas, biliares e fatores hormonais.^(241,242)^

A escolha dessa via deve ser priorizada em pacientes com TGI funcionante ou parcialmente funcionante, antes da indicação de nutrição parenteral, pois preserva o trofismo da mucosa intestinal.^(243)^

Langdana et al.^(244)^ observaram ser factível a terapia nutricional por meio de um programa intensivo de nutrição enteral na população pediátrica submetida a TCTH, incluindo pacientes que receberam condicionamento com TBI.

Outro estudo brasileiro observou que o uso de nutrição por sonda em crianças e adolescentes com câncer durante o TCTH é factível, não demonstrando complicações graves associadas à terapia. Complicações de menor gravidade ocorreram em 55% dos pacientes, as quais foram: intensificação dos episódios de vômitos ou diarreia com a progressão do volume de dieta (16%), deslocamento da sonda (19%), infecção fúngica na cavidade oral (9,7%) e obstrução da sonda (6,5%).^(242)^

Alguns ensaios consideraram a nutrição enteral tão eficaz quanto a parenteral, porém com menores taxas de complicações. Além disso, a enteral foi associada com melhor sobrevida, menor incidência de DECHa e DECHc e taxa de recuperação de neutrófilos mais acelerada associada a menor risco de infecção.^(227,243,245,246)^

Os dados de outro estudo sugerem que a terapia nutricional no período pré e durante o TCTH se correlacionou com melhor recuperação nutricional após o TCTH.^(227)^

A TNE tem sido amplamente recomendada para pacientes pediátricos submetidos ao TCTH, sendo a nutrição enteral por meio de sonda de alimentação a via preferencial, na ausência de toxicidade grave do TGI.^(244,247,248)^

A indicação da TNE por meio de sondas e ostomias ocorre na impossibilidade de utilização da via oral (mucosite graus 1 e 2), quando a ingestão alimentar for insuficiente (ingestão oral <70-80% das recomendações) por 3 a 5 dias consecutivos e insuficiente de alimentos e suplementos (abaixo de 100% das necessidades energéticas basais), associada com desnutrição ou perda de peso, considerando estado nutricional de risco, tempo de expectativa de melhora e tempo previsto para enxertia, desnutrição grave ou impedimento para o uso da alimentação oral.^(41,240)^ Para tanto, sugere-se que sejam realizados cálculos de ingestão alimentar diariamente, para que seja avaliada a indicação da TNE.

O atraso na indicação de terapia nutricional pode dificultar o uso da alimentação por sonda e predispor ao aumento do risco de complicações. Assim, a indicação precoce da alimentação por sonda pode beneficiar um número maior de pacientes, reduzindo a necessidade de nutrição parenteral, ou pelo menos seu tempo de uso e os riscos a ela relacionados.

É necessário considerar a fase de tratamento, a presença de toxicidade gastrintestinal, a condição clínica e o estado nutricional atual para desmame de nutrição enteral. A programação de retirada da terapia nutricional por sondas pode ser iniciada quando a ingestão oral atingir ≥70% a 80% das necessidades nutricionais calculadas por 2 ou 3 dias.

### Nutrição parenteral

Historicamente, a nutrição parenteral total foi o método mais comumente utilizado para fornecer nutrientes durante o TCTH. A importância da nutrição, em especial da parenteral, ficou bem evidenciada após a publicação dos resultados do estudo randomizado de Weisdorf et al.,^(249)^ demonstrando que a administração de nutrição parenteral profilática, durante o período do TCTH, aumentou a sobrevida no grupo que a recebeu, após 3 anos de acompanhamento.

No entanto, o uso da nutrição parenteral também está associado ao risco aumentado de complicações, especialmente de ordem infecciosa e metabólica, em particular entre pacientes com imunossupressão grave, como é o caso daqueles em TCTH.

Apesar de evidências de resultados nutricionais positivos com a oferta de nutrição parenteral total em crianças e adolescentes durante o TCTH,^(250)^ existem poucas pesquisas nesse contexto, e as informações sobre os efeitos da nutrição parenteral total nessa população são escassas. As recomendações são baseadas nos resultados dos estudos já discutidos aqui, incluindo os dados em adultos, que também fundamentam os princípios da terapia nutricional em crianças e adolescentes com câncer.

Em oncologia pediátrica, alguns diagnósticos e agentes neoplásicos, como a quimioterapia com tiotepa, melfalanoe cisplatina, e a radioterapia de corpo total tornam o paciente muito mais propensos a necessitar da via parenteral para nutrição do que outros.

Aspectos importantes relativos ao uso da nutrição parenteral são o monitoramento e o controle metabólico da oferta e o tipo de cateter utilizado. Como há um risco grande de alterações metabólicas, devido ao quadro inflamatório e de infecções por conta da manipulação dos cateteres, atenção especial deve ser dada ao manejo dessa terapia.

As principais indicações da nutrição parenteral ocorrem na impossibilidade total ou parcial de uso do TGI; na plaquetopenia grave não resolvida após infusão de plaquetas em pacientes que se encontrem em terapia enteral e na dificuldade de alcançar as necessidades nutricionais pela terapia de nutrição enteral plena em até 5 dias, considerando o estado nutricional e o tempo previsto para enxertia.

Por fim, o uso rotineiro da nutrição parenteral não é recomendado, a menos que a toxicidade ou as complicações graves do TGI impeçam a oferta plena pela via enteral.

### Algoritmos de terapia nutricional

Definir critérios para a tomada de decisão de terapia nutricional, melhorando os processos e garantindo que a terapia adequada traga mais benefícios do que complicações, é fundamental para o planejamento terapêutico dos pacientes.

De acordo com levantamento realizado sobre as vias de terapia nutricional, sugerem-se os seguintes algoritmos, separados de acordo com a fase do TCTH: algoritmo de terapia nutricional em pacientes pediátricos na fase pré-TCTH (Figura 2), algoritmo de terapia nutricional em pacientes pediátricos em TCTH (Figura 3) e algoritmo de terapia nutricional em pacientes pediátricos pós-TCTH (Figura 4).

**Figura 2:** Algoritmo de terapia nutricional em pacientes pediátricos na fase pré-transplante de células-tronco hematopoiéticas

**Figura 3:** Algoritmo de terapia nutricional em pacientes pediátricos em transplante de células-tronco hematopoiéticas

**Figura 4:** Algoritmo de terapia nutricional em pacientes pediátricos pós-transplante de células-tronco hematopoiéticas

### Uso de probióticos

Recentemente, a trajetória da microbiota intestinal vem sendo estudada em crianças durante o TCTH, bem como seu papel na DECHa, tendo sido já demonstrado que a diversidade microbiana intestinal pode predizer a sobrevida desses pacientes. O TCTH causa alteração estrutural e funcional temporária no ecossistema da microbiota intestinal, que mostra sinais de recuperação a partir de 100 dias após o transplante e agrava-se quando o DECH agudo se instala.^(251–253)^ Autores italianos reportaram a associação de DECH agudo a bactérias específicas, tanto durante a reconstrução da flora intestinal imediatamente após o TCTH, quanto, curiosamente, antes mesmo da realização do transplante. Em estudo publicado em 2015, amostras pré-transplante de pacientes que não tiveram DECH agudo mostraram maior abundância de um tipo de bactéria produtora de propionato, que persistiu à perturbação do ecossistema gerada pelo transplante. Tais resultados proporcionam uma perspectiva de manipulação da configuração da microbiota intestinal pré-TCTH, de modo a favorecer o sucesso do procedimento.^(252)^

O uso de probióticos durante e imediatamente após o TCTH é ainda questionado na prática clínica, devido à escassez de estudos que demonstrem sua segurança, especialmente para a pediatria, sendo supostamente alto o risco de translocação bacteriana devido ao enfraquecimento estrutural e imunológico da barreira intestinal.^(254–256)^ Em estudo americano publicado em 2016,^(257)^ foi demonstrado que o uso de *Lactobacillus plantarum* é seguro e viável durante o período de neutropenia em crianças e adolescentes em TCTH mieloablativo, sendo o primeiro estudo clínico a utilizar o probiótico nessa população, o que sugere promissores resultados com a citada cepa bacteriana nessa população.

Atualmente e enquanto não há mais estudos demonstrando segurança e benefícios do uso de probióticos para crianças e adolescentes submetidas ao TCTH, esse consenso contraindica a conduta nessa situação.

### Uso de glutamina

A glutamina é importante para funções fisiológicas, servindo como precursora na síntese de outros aminoácidos, como a arginina, atuando como importante antioxidante e combustível para a rápida proliferação celular do TGI, sistema imune, reticulócitos e fibroblastos, principalmente na formação e na manutenção da barreira da mucosa intestinal.^(258)^

É naturalmente encontrada na proteína proveniente da dieta, considerada não essencial; porém, em situações de estresse, torna-se um nutriente condicionalmente essencial, ou seja, em condições de aumento da demanda metabólica, como catabolismo, doença grave e prematuridade, o organismo não consegue produzir o suficiente.^(259)^

A administração em forma de suplemento pode ser realizada de forma oral ou endovenosa.

Quanto à administração de glutamina endovenosa, até o momento não existem evidências suficientes para recomendar seu uso; pelo contrário, relatos de toxicidade o tornam não recomendado.^(260)^ Desde a publicação dos últimos posicionamentos sobre o tema, dois novos estudos randomizados, controlados, porém com pequeno número de pacientes, foram publicados.^(261,262)^ Eles mostraram efeitos positivos da glutamina endovenosa sobre a permeabilidade intestinal e a lesão da mucosa pós-quimioterapia. Mais avaliações sobre o tema são aguardadas antes de uma mudança futura nas recomendações.^(263)^

Poucos são os estudos que analisam o uso da glutamina oral/enteral em crianças. Ward et al.,^(264)^ acompanharam 76 pacientes pediátricos em quimioterapia divididos em dois grupos, um deles recebendo 0,65g/kg de glutamina diluída em água uma vez ao dia por via oral ou enteral, iniciando no primeiro dia de quimioterapia. O estudo concluiu que a glutamina não reduziu a incidência e a gravidade da mucosite oral, mas mostrou redução no número de pacientes em nutrição parenteral.^(264)^ Revisão sistemática de 2016,^(265)^ com o objetivo de avaliar a função da glutamina oral na prevenção da mucosite em pacientes adultos submetidos à radioterapia e/ou quimioterapia, observou redução significativa na gravidade das lesões, com resolução do quadro em menor tempo e menos perda de peso nos indivíduos que fizeram uso do aminoácido. Doses máximas de 30g ao dia foram utilizadas, sem diferença dos efeitos colaterais em relação ao Grupo Controle sem glutamina. Para maior eficácia, a glutamina oral deveria ser iniciada zero a 7 dias antes da radio/quimioterapia, sendo dividida em três tomadas. Os autores chamaram a atenção para algumas limitações quanto à metodologia dos trabalhos analisados, como poucos pacientes, amostra não homogênea e trabalhos retrospectivos. Tal fato dificultou a identificação de quais são os pacientes que poderiam se beneficiar da glutamina oral e em qual fase do tratamento.^(263,266)^ Mais estudos podem definir se seu uso seria efetivo para todos os tipos de câncer e como coadjuvante para os esquemas medicamentosos oncológicos.

Não há consenso sobre a dose e a forma de administração do aminoácido. As doses variaram entre 7,5g a 30g ao dia antes do tratamento ou fracionada em três vezes ao dia. O tempo do uso também variou entre os estudos, podendo ser antes, durante ou depois do ciclo de quimioterápicos.^(267)^

De acordo com os levantamentos bibliográficos realizados, seguem, na tabela 28, as indicações para uso de glutamina em casos de mucosite e/ou trofismo intestinal.

**Tabela 28:** Resumo das indicações sobre o uso da glutamina e forma de administração

Glutamina	Mucosite	Trofismo intestinal
Endovenosa	Recomenda-se a não utilização de glutamina intravenosa na prevenção da mucosite oral em doentes que efetuaram quimioterapia em altas doses, com ou sem TBI, para o TCTH	Embora conflitantes, estudos recentes trazem resultados promissores. Necessidade de mais avaliações. Até o momento, não recomendada
Oral/enteral	Pode ser opção viável e de baixa toxicidade. A falta de um consenso em relação à dose, à forma de administração e ao tempo dificulta a padronização do seu uso	Sem evidências

Fonte: Arends J, Bachmann P, Baracos V, Barthelemy N, Bertz H, Bozzetti F, et al. ESPEN guidelines on nutrition in cancer patients. Clin Nutr. 2017;36(1):11-48;^(225)^ Lalla RV, Bowen J, Barasch A, Elting L, Epstein J, Keefe DM, McGuire DB, Migliorati C, Nicolatou-Galitis O, Peterson DE, Raber-Durlacher JE, Sonis ST, Elad S; Mucositis Guidelines Leadership Group of the Multinational Association of Supportive Care in Cancer and International Society of Oral Oncology (MASCC/ISOO). MASCC/ISOO clinical practice guidelines for the management of mucositis secondary to cancer therapy. Cancer. 2014;120(10):1453-61;^(260)^ Ward E, Smith M, Henderson M, Reid U, Lewis I, Kinsey S, et al. The effect of high-dose enteral glutamine on the incidence and severity of mucositis in paediatric oncology patients. Eur J Clin Nutr. 2009;63(1):134-40^(264)^ Storey B. The role of oral glutamine in pediatric bone marrow transplant. J Pediatr Oncol Nurs. 2007;24(1):41-5;^(268)^

TBI: irradiação corporal total; TCTH: transplante de células-tronco hematopoiéticas.

### Suplementação micronutrientes

Para a ótima utilização dos nutrientes, outras substâncias, chamadas micronutrientes, são necessárias. Essas substâncias são requeridas em mínimas quantidades, proporcionalmente baixas quando comparadas com sua grande influência no metabolismo e na saúde. Os micronutrientes são de dois tipos: elementos-traços e vitaminas. Os primeiros são elementos inorgânicos, enquanto as vitaminas são moléculas orgânicas complexas. Elementos-traços são essenciais e parte integrante das enzimas, sendo sete deles essenciais para a saúde humana: ferro, zinco, cobre, cromo, selênio, iodo e cobalto.^(269)^ As vitaminas são geralmente convertidas no organismo em moléculas complexas que funcionam como coenzimas, tendo várias funções no metabolismo, não podem ser sintetizadas e necessitam ser providas pela dieta.^(269)^

A suplementação vitamínica e mineral é importante durante todas as fases do TCTH, uma vez que os pacientes apresentam dificuldades na ingestão alimentar em longo prazo, agravadas pelas restrições dietéticas impostas pela dieta para neutropenia, sendo que as necessidades de algumas vitaminas e alguns minerais estão aumentadas.^(58)^ Além disso, devem-se considerar os hábitos alimentares das crianças, que, se inadequados em longo prazo, podem levar à deficiência de micronutrientes. Nas últimas décadas, tem-se observado, na população brasileira, um maior consumo de alimentos processados com alta densidade calórica e pobres em nutrientes essenciais, em substituição ao consumo de alimentos mais nutritivos, como frutas, verduras e legumes. Em decorrência das mudanças no padrão alimentar, os suplementos vitamínicos e/ou os alimentos enriquecidos tornam-se veículos práticos de vitaminas.^(223)^

Existem poucos estudos avaliando as concentrações séricas de vitaminas e minerais em crianças submetidas aos TCTH. Pesquisa recente em adultos encontrou deficiência de vitamina B1 na fase de pré-TCTH, além de deficiência de vitamina C e zinco na fase após o TCTH.^(270)^ Alguns nutrientes com propriedades antioxidantes, como as vitaminas C e E e o betacaroteno, estão depletados após o TCTH, assim como a vitamina B1 e o zinco,^(59,270)^ sendo que os pacientes apresentaram estresse oxidativo durante o condicionamento, especialmente no regime ciclofosfamida + TBI.^(271)^

Pacientes submetidos ao TCTH alogênico também estão sob o risco aumentado de deficiência de tiamina (vitamina B1), que leva a alterações metabólicas, como a acidose láctica,^(272)^ e neurológicas, como a encefalopatia de Wernicke, devendo ser suplementados em doses superiores à RDA durante o período imediato após o TCTH. A absorção da vitamina B12 também está reduzida devido aos efeitos do condicionamento ou em consequência da DECH.^(82,273,274)^ Estudo brasileiro envolvendo crianças e adolescentes submetidos ao TCTH alogênico encontrou alta prevalência de deficiência de vitamina D já no pré-TCTH, com redução dos níveis séricos após 6 meses.^(152,275)^ Estudos em adultos têm sugerido que as propriedades imunomoduladoras dessa vitamina poderiam ter papel importante na prevenção e no tratamento da DECH.^(276)^

A oxidação lipídica pode ser inibida com suplementação de vitamina C associada à vitamina E (800UI a 1.000UI) e ao carotenoide (45mg).^(59)^ Outra vantagem da suplementação da vitamina E seria a prevenção à DVO hepática.^(59)^ A vitamina C deve ser suplementada visando também à promoção da recuperação tecidual pela biossíntese de colágeno pós-condicionamento, sendo a recomendação diária de 250mg para pacientes abaixo de 31kg e 500mg para pacientes com mais de 31kg, com contraindicação em casos de ferritina sérica maior de 1.000mg/L.^(277)^ A suplementação de vitamina K também é necessária; durante o processo de TCTH, sua deficiência está relacionada ao uso de algumas medicações. No período de condicionamento, muitas crianças recebem fenitoína como profilaxia de convulsões, e essa medicação é antagonista da vitamina K.^(277)^ Além disso, alguns antibióticos, como as cefalosporinas, podem inibir diretamente a enzima epóxido-redutase hepática, causando antagonismo a essa vitamina.^(278)^

A recomendação para pacientes submetidos ao TCTH consiste em uso de suplementos contendo todas as vitaminas e alguns minerais, como cálcio, zinco, selênio e isento de ferro, respeitando-se a quantidade da DRI por, no mínimo, 1 ano após o TCTH.^(276)^ A suplementação de ferro geralmente não é recomendada, porque grande parte dos pacientes possuei concentrações elevadas de ferro devido a frequentes transfusões sanguíneas.^(277)^ Existem poucas opções de suplementos polivitamínicos específicos para pediatria no mercado brasileiro, sendo uma alternativa o uso de fórmulas manipuladas individualizadas.

Em algumas situações especiais, as suplementações de micronutrientes devem ser revistas e individualizadas. Pacientes com DECHa de pele graus 3 e 4 devem receber doses extras de algumas vitaminas e minerais para promoção da cicatrização e recuperação tecidual,^(277)^ e a necessidade de nutrientes pode ser comparada às de um grande queimado.

Em casos de diarreia volumosa, a suplementação adicional de zinco é necessária para repor as perdas aumentadas, na dose de 10mg a 12mg por litro de fezes.^(223,269,277)^ Devemos lembrar que o zinco é um elemento-traço essencial, importante para o crescimento, a cicatrização de feridas, a manutenção do sistema imunológico e outras funções vitais, devendo ser adicionado a toda nutrição parenteral total na dose de 100mcg/kg para crianças e de 3mg a 4mg para adolescentes.^(269)^ Em pacientes com disfunção hepática (com bilirrubina >10mg/dL), é necessária cautela na adição de cobre e magnésio na nutrição parenteral total.^(278,279)^

## CONCLUSÃO

O paciente pediátrico submetido ao transplante de células-tronco hematopoiéticas pode apresentar inúmeras complicações no decorrer e após o procedimento. Este documento pretende empoderar equipes de suporte na tomada de decisão da terapia nutricional adequada ao indivíduo, considerando necessidades básicas da faixa etária, bem como específicas nesse momento.

A presença da avaliação nutricional completa (anamnese de consumo alimentar, avaliação antropométrica, parâmetros laboratoriais e exame físico) no pré (identificando carências nutricionais e programando a terapia nutricional inicial), durante (acompanhando complicações e programando estratégias de intervenção nutricional) e no pós-alta hospitalar no acompanhamento ambulatorial (acompanhamento ingestão alimentar, sintomas e prezando uma alimentação adequada para idade) cria uma rede de vigilância para possíveis complicações nutricionais.
